# Orthogonally aligned cyclic BODIPY arrays with long-lived triplet excited states as efficient heavy-atom-free photosensitizers†

**DOI:** 10.1039/d1sc04893g

**Published:** 2021-10-29

**Authors:** Zhaoyang Zhu, Xue Zhang, Xing Guo, Qinghua Wu, Zhongxin Li, Changjiang Yu, Erhong Hao, Lijuan Jiao, Jianzhang Zhao

**Affiliations:** Laboratory of Functionalized Molecular Solids, Ministry of Education, College of Chemistry and Materials Science, Anhui Normal University Wuhu 241002 China jiao421@ahnu.edu.cn haoehong@ahnu.edu.cn; State Key Laboratory of Fine Chemicals, School of Chemical Engineering, Dalian University of Technology Dalian 116024 China zhaojzh@dlut.edu.cn

## Abstract

In photosensitizers, long triplet excited state lifetimes are key to their efficient electron transfer or energy transfer processes. Herein, we report a novel class of cyclic trimeric BODIPY arrays which were efficiently generated from easily accessible *meso*-mesityldipyrrinone and arylboronic acids in one pot. Arylboronic acid, for the first time, was used to provide a boron source for BODIPY derivatives. Due to the well-defined and orthogonally aligned BODIPY cores as verified by X-ray crystallography, these BODIPY arrays show strong exciton coupling effects and efficient intersystem crossings, and are novel heavy-atom-free photosensitizers with a long-lived triplet excited state (lifetime up to 257.5 μs) and good reactive oxygen species generation efficiency (up to 0.72) contributed by both ^1^O_2_ and O_2_^−^˙ under light irradiation.

## Abstract 2

Cyclic BODIPY trimers showed strong exciton coupling in singlet excited states and long-lived triplet excited states, and generated both singlet oxygen and superoxide radicals under light irradiation, giving good reactive oxygen quantum yields and promising PDT results *in vitro*.

## Graphical abstract

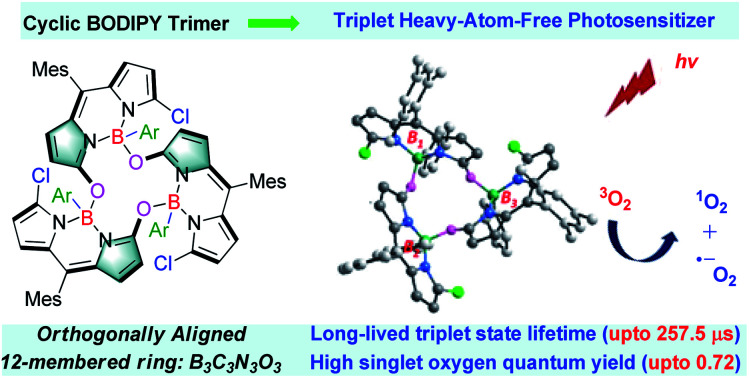

## Introduction

**Fig. 1:**
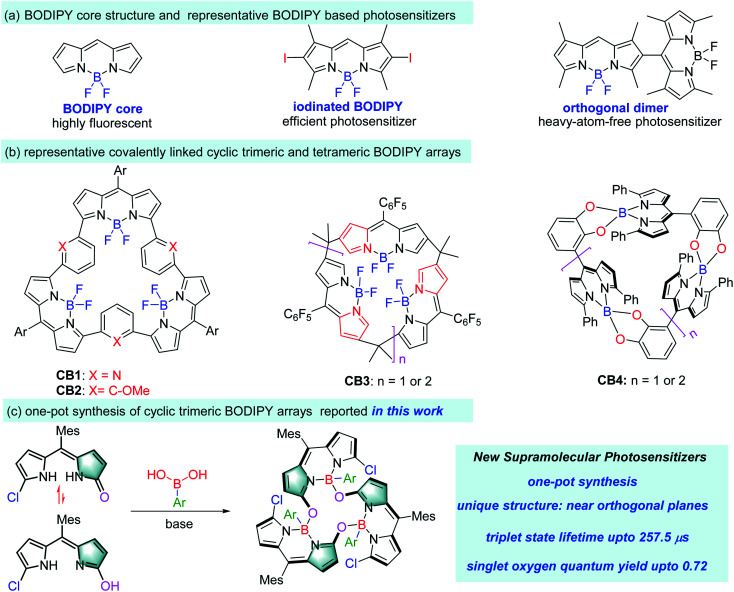
(a) Chemical structures of BODIPY, a representative iodinated BODIPY and an orthogonal BODIPY dimer, (b) reported cyclic trimeric and tetrameric BODIPY arrays **CB1–CB4**, and (c) one-pot synthesis of cyclic trimeric BODIPY arrays from *meso*-mesityldipyrrinone and arylboronic acids reported in this work.

Chromophore arrays with well-defined architectures possess unique photophysical properties and have attracted wide research interest in a variety of research fields.^1^ Among those, porphyrinoid-based chromophore arrays,^2^ especially the porphyrin-based dimers and trimers (Fig. 1a), have been extensively investigated as mimics of natural light-harvesting antennae, related energy transfer cassettes and photosensitizers.^3^ BODIPY^4^ (boron dipyrromethene, Fig. 1a) as “the little sister of porphyrin” can be viewed as a boron complex of a half porphyrin framework. It features a rigid structure, easy accessibility, reasonably good stability and excellent photophysical properties, including strong photon capture ability which is easily tuneable from the visible to near infrared range.^4*c*–*g*^ It also has rich chemistry and multiple sites (six pyrrolic, one *meso*- and one boron) for the facile incorporation of various functionalities at different positions of the BODIPY platform to meet a set of specific requirements for different applications, from fluorescence probes (for metal ions,^5^ biomolecules,^6^ viscosity,^7^ polarity^8^ and temperature^9^ within living cells) to photosensitizers for photodynamic therapy (PDT).^10^ Therefore, BODIPY is an appealing platform for the construction of chromophore arrays.

However, unlike porphyrin arrays, currently BODIPY arrays are relatively unexplored, and the most reported ones are linear BODIPY arrays with low structural rigidity.^11,12^ Covalently linked cyclic BODIPY arrays can fix the rigidity issue since each of the BODIPY subunits is locked by their adjacent ones. Due to the synthetic challenge, there are only a handful of cyclic BODIPY arrays (for example, **CB1** to **CB4**, Fig. 1b)^13–16^ available. Their synthesis is mainly based on the boron complexation of premade porphyrin analogues. **CB1** and **CB2** with a bowl-shaped structure, both from BF_2_ complexation of expanded porphyrins,^13^ showed interesting exciton coupling between the BODIPY subunits and strong recognition of guest species. **CB3** was prepared from BF_2_ complexation of expanded *N*-confused calix[*n*]phyrins,^14^ and exhibited unique luminescence and lasing properties. Two types of cyclic BODIPY arrays were also synthesized from BODIPY monomers through self-coupling or condensation with pentafluorobenzaldehyde.^15^ A sole example of elegant cyclic BODIPY array **CB4** linked through boron atoms was reported by Nabeshima and co-workers^16^ using the reaction of catecholyldipyrrin with boron trichloride. Despite the low yields (less than 5%) of this one-pot reaction, the resultant **CB4** was a nice supramolecular host for alkali metal ions. In general, these reported cyclic BODIPY arrays typically showed strong excitonic coupling of the singlet excited state due to their closely packed structures.^11–17^ However, such systems with fine-tuned triplet excited states have not been developed.

Due to their promising photophysical properties, BODIPY dyes have been transformed to triplet photosensitizers through improving their intersystem crossing (ISC) from the singlet state to triplet state.^18,19^ The majority of these BODIPY based photosensitizers relied on the installation of heavy atoms to induce ISC and the consequent singlet oxygen formation (for example, iodinated BODIPY^18*h*^ in Fig. 1a). However, despite having good singlet oxygen quantum yields, these halogenated BODIPYs typically show short triplet state lifetimes due to the enhanced and undesired ISC from the T_1_ state to the S_0_ state.^19^ Long triplet state lifetimes of photosensitizers are key to efficient electron transfer or energy transfer processes for application in photodynamic therapy (PDT).

Herein, we report a series of calixarene-like cyclic trimeric BODIPY arrays based on the direct condensation of *meso*-mesityldipyrrinones with arylboronic acids, and they show efficient ISC due to the orthogonally aligned BODIPY cores, good singlet oxygen generation efficiency and long-lived triplet excited states (Fig. 1c). To the best of our knowledge, this is the first time that a cyclic BODIPY array has been used to fine-tune its triplet excited state to produce an efficient supramolecular photosensitizer with a long triplet state lifetime.

## Results and discussion

**Scheme 1:**
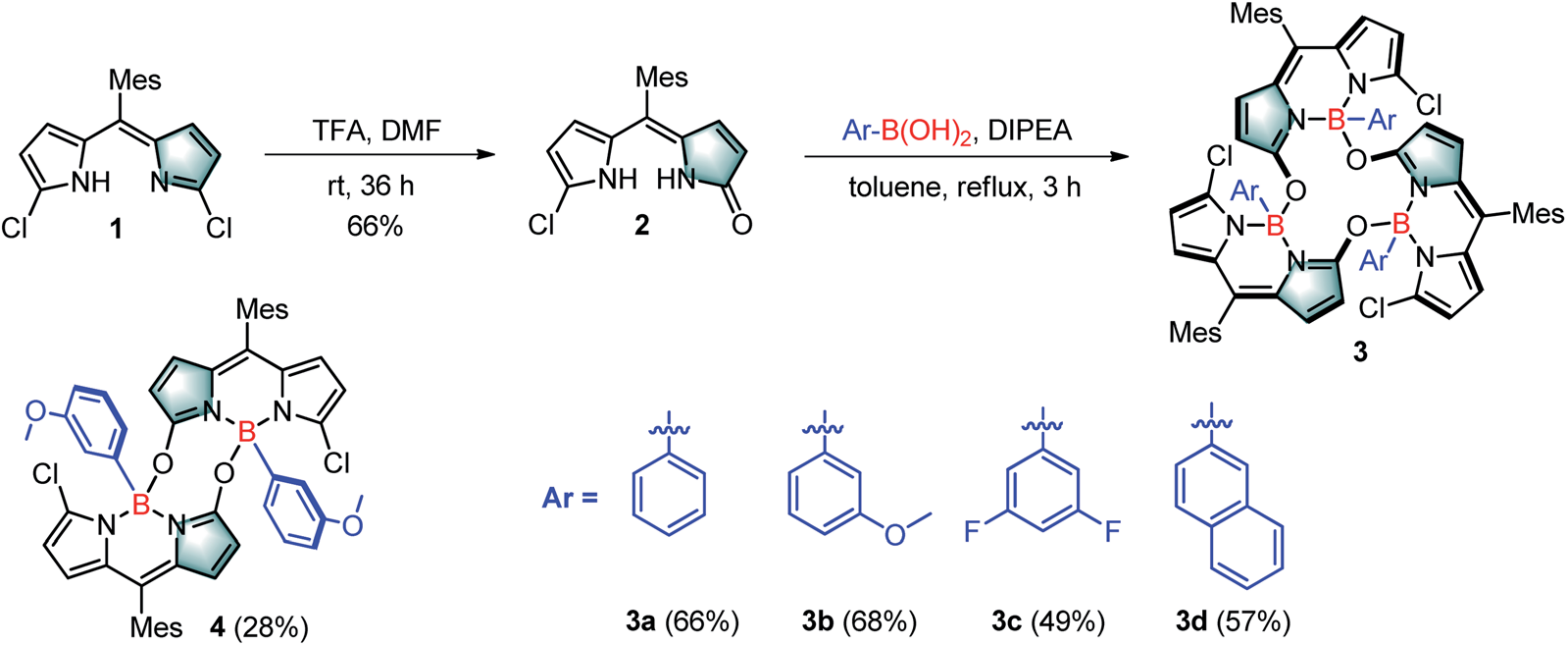
Synthesis of cyclic BODIPY trimers **3a–d** and dimer **4**.

Inspired by our recent synthesis of boron-functionalized BODIPYs through organotrifluoroborate salts,^20^ we rationalized that arylboronic acids may also be able to complex with α-hydroxydipyrrin (or its tautomer dipyrrinone) and undergo further condensation to generate novel cyclic BODIPYs with B–O linkages (Fig. 1c). Therefore, we chose its analogue, *meso*-mesityldipyrrinone **2**, as the starting material, which was smoothly generated as yellow powder in 66% yield within one step from the acid-catalyzed hydrolysis (using TFA, trifluoroacetic acid, as the acid) of readily available 1,9-dichlorodipyrrin **1**^21^ in DMF at room temperature (Scheme 1). Initially, commercial phenylboronic acid was chosen for the subsequent complexation with *meso*-mesityldipyrrinone **2**. It was performed in refluxing toluene in the presence of 1 equiv. of DIPEA (diisopropylethylamine), from which a major product was isolated as an orange solid in 66% yield, and was fully characterized by ^1^H NMR, ^13^C NMR and HRMS. The ^1^H NMR spectrum shows sharp and simple pattern signals, indicating a symmetric structural feature. In comparison with that of the starting *meso*-mesityldipyrrinone **2**, two NH signals (at 9.99 and 10.69 ppm) are missing and high-field shifted doublet signals are observed for the pyrrolic β-proton (Scheme 1, H_1_, 4.80–4.90 ppm with respect to the 6.58–6.07 ppm for **2**). These results indicate the successful complexation with phenylboronic acid. HRMS-ESI mass analysis gives a strong peak at *m*/*z* 1219.3995, which indicates the formation of cyclic trimeric BODIPY **3a** (calcd for C_72_H_60_B_3_Cl_3_N_6_O_3_Na^+^ [M + Na]^+^: 1219.3980). The structure of **3a** was further confirmed by X-ray analysis.

**Fig. 2:**
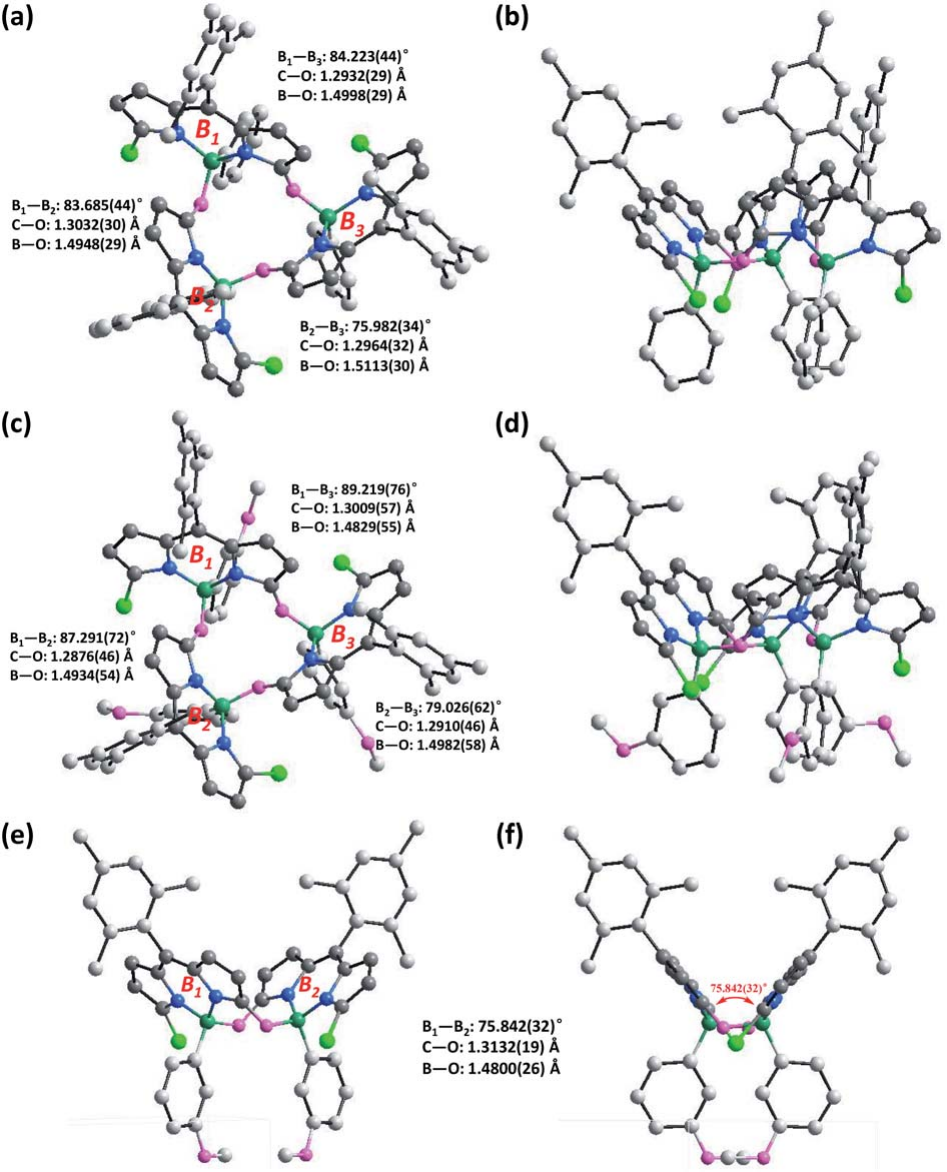
X-ray crystal structures of (a and b) BODIPY trimer **3a**, (c and d) BODIPY trimer **3b** and (e and f) BODIPY dimer **4**. C, light gray; N, blue; B, dark green; Cl, bright green; O, pink. Hydrogen atoms have been removed for clarity.

Crystals suitable for the X-ray analysis of **3a** were obtained by slow evaporation of its dichloromethane solution. X-ray analysis results clearly show that the α-hydroxyl moiety (derived from the ketone moiety of *meso*-mesityldipyrrinone **2**) on each BODIPY chromophore is bonded to the central boron atoms of two adjacent BODIPY chromophores (Fig. 2a and b). The dihedral angles between these BODIPY subunits approach orthogonality (76°–84.2°), similar to those of the reported orthogonal BODIPY dimers (Fig. 1a).^10*b*^ Meanwhile, the two β-pyrrolic protons (H_1_) are located in a ring system, which leads to a shielding effect from the diatropic ring currents residing on the adjacent BODIPY units.^3*a*^ This might explain the observed high-field-shift of pyrrolic protons in the ^1^H NMR spectra of **3a** with respect to that of *meso*-mesityldipyrrinone **2**.

To test the versatility of this strategy, we further applied some other commercial arylboronic acids, including phenylboronic acid derivatives with electron-rich methoxyl or electron-deficient fluoro-substituents, and more conjugated **2**–naphthaleneboronic acid for this reaction (Scheme 1). In all the cases, the complexation smoothly proceeded and generated the desired corresponding cyclic trimeric BODIPYs **3b–d** in 49–68% isolated yields (see the ESI† for their detailed synthesis). These target products were fully characterized by ^1^H NMR, ^13^C NMR and ESI. Similar to **3a**, these products all show sharp and simple spectrum patterns in their ^1^H NMR spectra, which indicates their symmetric structural features. In addition, the missing of two NH signals (at 9.99 and 10.69 ppm) and high-field shifted doublet proton signals for the pyrrolic β-protons in the range of 4.80–4.90 ppm are observed. HRMS-ESI mass analysis shows a strong [M + Na]^+^ peak at *m*/*z* 1307.4303 for **3b** (calculated 1307.4281), 1303.3583 for **3c** (calculated 1303.3579), and 1369.4454 for **3d** (calculated 1369.4455), respectively. Among those, the structure of **3b** is further confirmed by X-ray analysis (Fig. 2). Similar to **3a**, all the B–O bond lengths within **3b** are around 1.49(1) Å (Table S1†), and the two β-pyrrolic protons (H_1_) are located in a ring system. This might explain the observed high-field-shift of the pyrrolic protons in the ^1^H NMR spectra of **3b** with respect to that of dipyrrinone **2**. The dihedral angles between each of the BODIPY subunits are slightly larger (around 89.2°) than those observed in **3a** (84.2°) (Fig. 2c and d), which indicates that the substituents on the arylboronic acid can tune the dihedral angles between each of the BODIPY subunits.

**Fig. 3:**
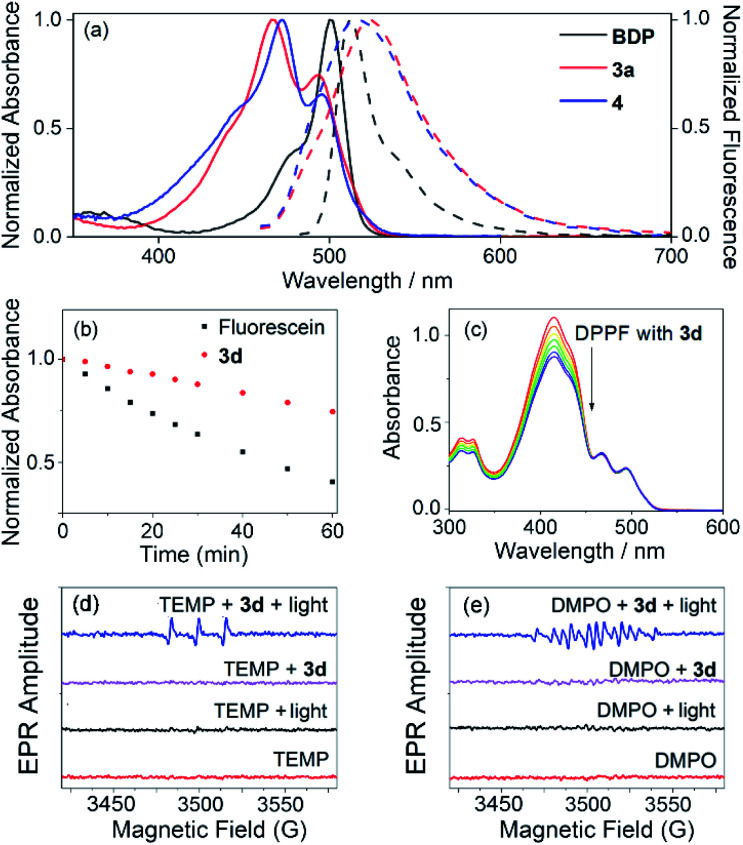
(a) Overlaid normalized absorption (solid lines) and emission (dashed lines) of compounds BDP, **3a**, and **4** recorded in CH_2_Cl_2_ at room temperature. (b) Absorbance changes at 491 nm of fluorescein (2 × 10^−5^ M) in 0.1 M NaOH, and **3d** (2 × 10^−5^ M) in toluene under strong continuous irradiation with a 50 W white LED lamp over 1 h. (c) Changes in the absorption spectrum of DPBF upon irradiation with a 490 nm laser in the presence of **3d** in CH_2_Cl_2_ at room temperature (recorded at 30 s intervals). (d and e) EPR spectra of **3d** (0.3 mM, in dichloromethane) for ^1^O_2_ characterization with 2,2,6,6-tetramethyl-4-piperidone (TEMP, 50 mM) or for O_2_˙^−^ characterization with 5,5-dimethyl-1-pyrroline-*N*-oxide (DMPO, 1 M) as the spin-trap agent under different conditions.

Interestingly, the reaction of 3-methoxyphenyl boronic acid with *meso*-mesityldipyrrinone **2** also generated a reasonably stable minor product **4** in 28% yield besides **3b** (68% isolated yield). The yield of this minor product **4** can be further tuned *via* simple variation of the amount of the DIPEA base in the system. It was characterized by ^1^H NMR, ESI mass and X-ray analysis. A strong peak at *m*/*z* 857.3004 in ESI mass indicates the formation of a dimeric BODIPY framework. Its dimeric BODIPY structure was further confirmed by X-ray analysis, in which the two BODIPY subunits stay far away from each other with few overlaps of the their BODIPY chromophores. In great contrast to the cyclic trimeric BODIPY **3b**, no high-field-shift of pyrrolic protons was observed in its ^1^H NMR spectra. In comparison with trimeric **3b**, dimeric **4** shows lower photostability which can be observed with the naked eye. Initially, **4** is nonfluorescent in organic solvent. Upon strong light irradiation under vacuum, there is a dramatic turn-on of fluorescence in its solution. By contrast, the continuous strong irradiation of **3d** with a 50 W white LED lamp in toluene for a period of 60 min leads to less than 25% degradation of this molecule (Fig. S12†). Similar to the photodegradation phenomena of classical BODIPY dyes,^22*a*^ we did not observe any change in the shape of the longest wavelength absorption band of **3d**, except for the decrease in peak heights, during the above photoexposure. It is worth noticing that **3d** is even more stable than the well-known commercial fluorescein, which gives around 60% degradation under the same irradiation conditions (Fig. 3b).

Cyclic trimeric BODIPYs **3** each shows a dual intense broad absorption in the common organic solvents studied (as summarized in Fig. 3, Table S4, and Fig. S7–S10†). For example, **3a** displays dual absorption bands centered at 467 and 493 nm, respectively (molar absorption coefficient *ε* = 8.71 × 10^4^ M^−1^ cm^−1^ at 467 nm; Table S4†). Similarly, **4** also shows dual absorption bands centered at 472 and 497 nm (Fig. 3a), respectively. However, the absorption efficiency is slightly lower (*ε* = 3.09 × 10^4^ M^−1^ cm^−1^ at 472 nm) with respect to that of **3a**. These results are in great contrast to those of their monomeric *meso*-mesityl BODIPY (**BDP**, Fig. 3a),^22*b*^ which shows only a maximum absorption peak at 498 nm in dichloromethane. These dual absorption bands indicate the existence of exciton coupling in these cyclic dimeric and trimeric BODIPYs.^11,12^

Cyclic trimeric BODIPYs **3** all show extremely low fluorescence (*ca. Φ* = 0.001) in the various common organic solvents studied (Table 1), which is also in agreement with the extremely low oscillator strengths for their electronic transitions from theoretical calculations (Table S7†). Inspired by the orthogonally aligned BODIPY cores observed in our cyclic trimeric BODIPYs, we envisaged that ISC would be possible for these molecules since the other nonradiative decay pathways of the S_1_ state would be less efficient for these rigid cyclic molecules. The calculated S_1_ energy for the parent trimeric BODIPY **3** (*meso*-mesityl group and the boron substituted aryl group were removed for simplicity) was 2.48 eV, while the calculated T_1_, T_2_, T_3_ and T_4_ energies were 1.53, 1.53, 1.53 and 2.48 eV, respectively. Since T_1_, T_2_ and T_3_ are below the S_1_ state, and T_4_ is the same as S_1_, S_1_ → T_1_, S_1_ → T_2_, S_1_ → T_3_ and S_1_ → T_4_ are considered to be the possible pathways of ISC. To investigate the underlying reason for the enhanced ISC in these trimeric BODIPYs, the spin–orbit coupling (SOC) matrix elements between the singlet state and the triplet excited states for **3** were further calculated as 0.74, 0.83 and 1.58 cm^−1^ for T_1_, T_2_ and T_3_ states, respectively (Table S10†), which were considered large enough to induce ISC.^10*b*–*e*^ Furthermore, the 〈S_1_|Ĥ_SO_|T_3_〉 value (Table S12†) found for **3** (1.58 cm^−1^) is almost 1 order of magnitude higher than the 〈S_1_|Ĥ_SO_|T_4_〉 value (0.19 cm^−1^). Then, natural transition orbital (NTO) analysis was conducted to further investigate the excited state properties. The spin–orbit charge transfer ISC (SOCT-ISC) mechanism^23^ is found to be preferred for **3**, and charge transfer occurs more efficiently from a singlet CT state (S_1_) to a triplet LE state (T_3_) due to a relatively large SOC value (Fig. S22–S24†).

**Table 1:** Photophysical properties of compounds **3** and **4** in dichloromethane at 20 °C

Dyes	*λ*_abs_ (nm)	*λ*_em_ (nm)	lg *ε*_max_^c^	Stokes shift (cm^−1^)	*Φ*_F_ d	*Φ*_Δ_ e	*τ*_T_ f
**3a**	467^a^/493^b^	523	4.94	1164	<0.001	0.35	257.5
**3b**	468^a^/494^b^	523	4.91	2247	<0.001	0.72	—g
**3c**	471^a^/496^b^	526	4.82	2220	<0.001	0.34	—
**3d**	468^a^/494^b^	521	5.16	1049	<0.001	0.41	188.9
**4**	472^a^/497^b^	515	4.49	1769	0.002	—	—

a Maximum absorption peak.

b Shoulder peak.

c Molar absorption coefficients correspond to the maximum absorption peak.

d Fluorescence quantum yields were obtained by using fluorescein (*Φ*_F_ = 0.90 in 0.1 M NaOH) as the reference. The standard errors are less than 10% based on three measurements.

e Singlet oxygen quantum yield with **RB** (*Φ*_Δ_ = 0.80 in methanol) as the standard; determination error = ±0.03.

f Triplet state lifetime in deaerated dichloromethane, *λ*_ex_ = 490 nm. **RB** was used as the standard compound, *Φ*_Δ_ = 80% in methanol.

g Not measured.

The singlet oxygen generation efficiency of these trimeric BODIPYs was tested under broadband light (*ca.* 490 nm) irradiation conditions by using 1,3-diphenylisobenzofuran (DPBF) as the trap molecule and Rose Bengal (**RB**, *Φ*_Δ_ = 0.80 under the investigated conditions) as the reference compound, respectively (Fig. 3c, and Table S5 in the ESI†). As expected, the cyclic trimeric BODIPYs **3** show reasonably good to high singlet oxygen generation efficiencies (*Φ*_Δ_ = 0.35, 0.72, 0.34 and 0.41 for **3a**, **3b**, **3c** and **3d** in dichloromethane, respectively). Among those, the singlet oxygen quantum yield for **3b** is much higher than those of nonhalogenated BODIPY dyes and many other organic chromophores and photosensitizers under comparable conditions. Notably, both HOMOs and LUMOs for **3a** and **3c** are distributed over only the BODIPY cores. However, for **3b**, only the LUMO is distributed on the BODIPY cores, while the HOMO is located on the 3-methoxylbenzene group (Fig. S25†), indicating a possible intramolecular charge transfer (ICT) process^24^ in this trimer. The efficient ICT in **3b** may facilitate the SOCT ISC process *via* efficient photoinduced charge separation,^23^ providing its high singlet oxygen generation efficiency. The singlet oxygen quantum yield values in toluene and THF solutions are also satisfactory for those trimeric BODIPYs (Table S6†).

Next, EPR spectra were used to track the reactive oxygen species formed during light irradiation of our cyclic trimer **3d**. TEMP, an ^1^O_2_ specific spin-trap agent, was added to the solution of **3d**, and a strong EPR signal was observed (Fig. 3d) after light irradiation of the solution mixture. Without light or addition of TEMP, no EPR signal was found. This characteristic EPR signal of the paramagnetic adduct from ^1^O_2_ and TEMP^25^ indicated that cyclic trimer **3d** generated ^1^O_2_ efficiently under light irradiation. Furthermore, when DMPO, an O_2_^−^˙ specific spin-trap agent, was added to the solution of **3d**, a characteristic EPR signal of the paramagnetic adduct from O_2_^−^˙ and DMPO^25^ was also observed upon light irradiation (Fig. 3e), indicating the production of O_2_^−^˙ by cyclic trimer **3d** under light irradiation. Thus, the *Φ*_Δ_ values of our cyclic trimers should be contributed by both ^1^O_2_ and O_2_^−^˙ through energy transfer and electron transfer pathways, respectively.

**Fig. 4:**
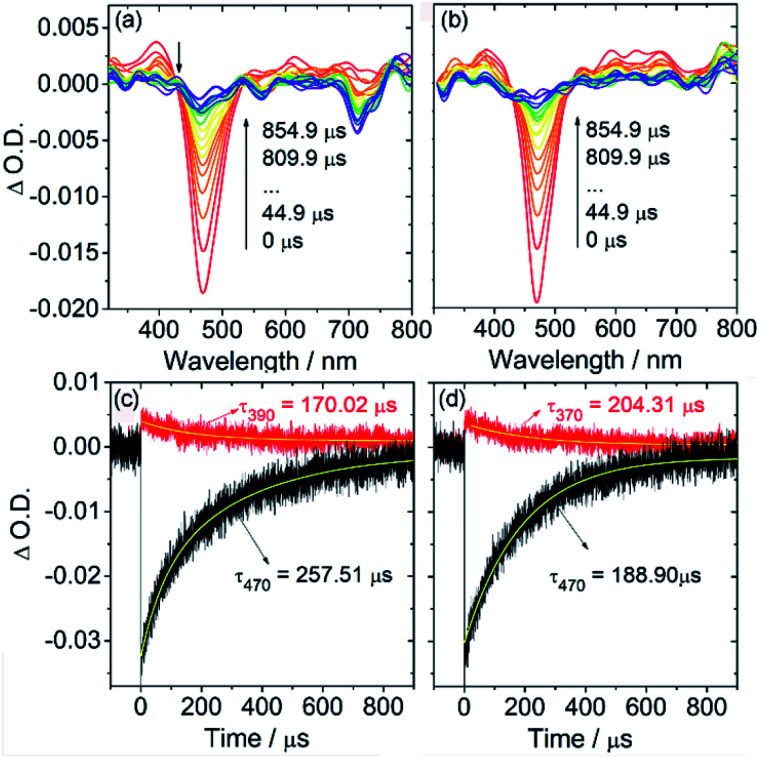
Nanosecond transient absorption spectra of (a) **3a** and (c) the decay trace; (b) **3d** and (d) the decay trace. *c* = 1.0 × 10^−5^ M. *λ*_ex_ = 490 nm, in deaerated dichloromethane, 20 °C.

BODIPYs **3a** and **3d** were then selected to study their triplet-state properties with nanosecond transient absorption (ns TA) spectroscopy (Fig. 4). Upon pulsed laser excitation at 490 nm, a ground-state bleaching (GSB) band at *ca.* 470 nm was observed along with excited-state absorption (ESA) bands in the range of 300–800 nm (superimposed with the GSB band). These ESA bands are different from the typical BDP triplet-state ESA bands centered at *ca.* 420 and 675 nm. By monitoring the decay of the GSB signal at 470 nm, the triplet excited states for **3a** and **3d** were found to be long-lived (hundreds of microseconds, 257.5 and 188.9 μs) in deaerated solution (Fig. 4c and d). These triplet state lifetimes are much longer than those of typical heavy atom-containing triplet photosensitizers with comparably long-wavelength absorption (*t*_T_ = 1.7 μs for 2,6-diiodo-bisstyrylBODIPY).^26^ This long-lived triplet state lifetime is essential for their applications in photocatalysis and PDT since the triplet state lifetimes of photosensitizers would greatly affect their intermolecular diffusion-controlled triplet energy transfer or electron transfer efficiency.^18,19^

To enhance the biocompatibility and solubility of the cyclic trimeric BODIPY, classical Cremophor-EL surfactant was used to obtain the self-assembly of micelles of **3b** and **3d** (Fig. S26†). The absorption spectra of **3b**/**3d** micelles in water were similar to their spectra in dichloromethane, suggesting that **3b**/**3d** micelles could disperse in the aqueous phase well. Transmission electron microscopy (TEM) images showed that the diameter of **3d** micelles was about 55 nm (Fig. S27†), which was also confirmed by the dynamic light scattering (DLS) results (Fig. S26†). Furthermore, stabilities of both **3b** and **3d** in water toward pH and storage were evaluated using absorption spectra. There was no significant difference in the absorptions of **3b**/**3d** micelles in solutions with different pH values in the range of 3–10 (Fig. S28†). What's more, although these micelles were stored for 48 h in buffer solutions with different pH values, the absorbance only exhibited negligible declines (Fig. S29 and S30†). These results suggested that similar to classical BODIPY dyes,^27^ these cyclic BODIPY trimers have promising stabilities to cope with different physiological pH conditions in practical applications.

**Fig. 5:**
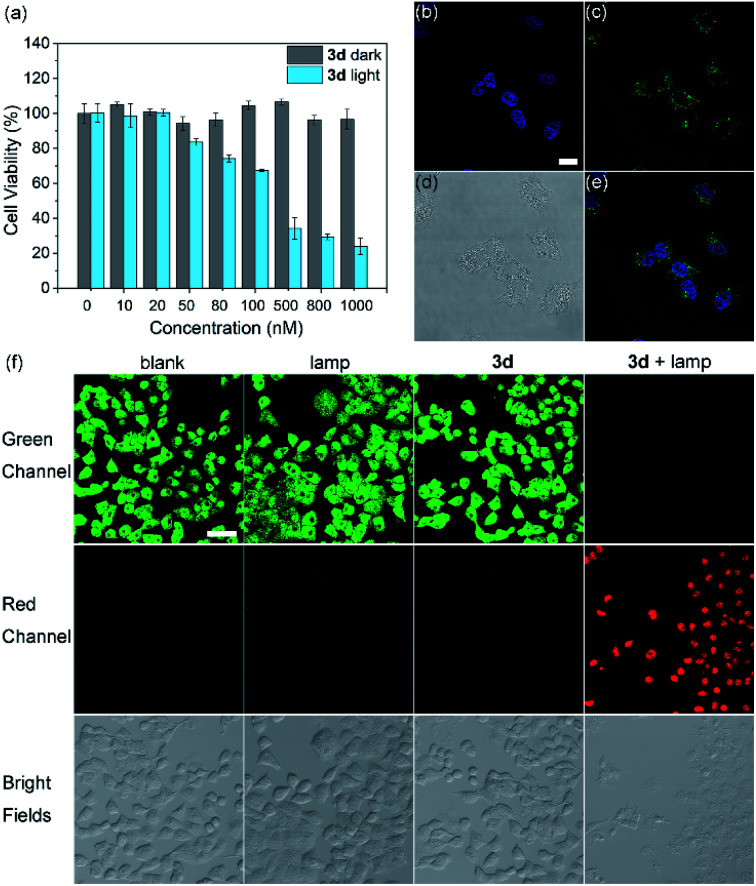
(a) Photocytotoxicity and dark cytotoxicity of **3d** micelles demonstrated by CCK-8 assay. Cell suspensions were seeded in 96-well flat-bottom plates and varying concentrations of the sensitizers were added to each well. Cells were kept either in the dark or under illumination with a cyan LED lamp at a flow rate of 2100 lux for a period of 3 h at 37 °C. (b–e) Confocal fluorescence images of HeLa cells stained with **3d** micelles (10 μM) and DAPI: (b) 4′,6-diamidino-2-phenylindole (DAPI, cell nucleus-specific dye) fluorescence; (c) **3d** micelle fluorescence after incubation for 1 h; (d) bright field image; (e) merged images of parts (b) and (c); scale bar: 20 μm. (f) Confocal fluorescence images of acridine orange (green channel, live cell marker) and propidium iodide (red channel, dead cell marker) co-stained HeLa cells incubated with 0 (lamp only group) or 1 μM **3d** micelles (**3d** only group and **3d** with lamp group) without and with light illumination. Scale bar: 200 μm.

When HeLa cells were incubated with **3d** micelles for 1 h, weak green fluorescence was observed under a confocal fluorescence microscope (Fig. 5c), indicating that **3d** micelles could penetrate the cell membrane. Next, a CCK-8 assay was conducted to determine the dark-toxicity of **3d** micelles to living HeLa cells. The results showed that **3d** possesses no significant cytotoxicity even at a concentration of 30 μM, and the IC_50_ was 68.4 μM (Fig. S31†) indicating the good biocompatibility of **3d** micelles. The photocytotoxicity of the cyclic trimeric BODIPY **3d** micelles was further investigated (Fig. 5a). Even at very low concentrations of **3d** micelles, a significant decrease of cell viability was observed with a remarkable IC_50_ of 162 nM (Fig. S34†). By contrast, there was no statistically significant change when the cells were kept in the dark in the presence of the same concentration of the photosensitizer **3d** micelles (gray, Fig. 5a). Similarly, **3b** micelles were also studied, which also showed an effective IC_50_ value of 246 nM (Fig. S33†). Furthermore, under these conditions, the photocytotoxicity of the reference photosensitizer **RB** was studied, which showed an IC_50_ of 1.2 μM (Fig. S32†). From the above results, the PDT treatment window of **3d** micelles was 162 nM to 74.8 μM. Such a wide range is far superior to those of heavy atom effect type photosensitizers, which suffer from severe dark toxicity resulting from heavy atoms and a short treatment window.

To visualize the PDT effect of **3d** micelles toward cancer cells, the phototoxicity of **3d** micelles was also evaluated using the fluorescence images of live/dead co-stained cells (Fig. 5f). Acridine orange (AO, green channel) tends to combine with living cells and emits green fluorescence, while propidium iodide (PI, red channel) selectively combines with dead cells and glows with red fluorescence because of changes in membrane permeability. In the control groups, the cells which were treated with **3d** micelles or LED lamp illumination alone or without any treatment emitted bright green fluorescence, indicating that the dark cytotoxicity of **3d** micelles was low, and the thermal side effect of LED lamp irradiation could be ignored (blank, lamp only and **3d** only groups in Fig. 5f). In contrast, when **3d** micelles were illuminated with an LED lamp, the cells emitted brilliant red fluorescence and negligible green fluorescence, suggesting that nearly all cells were dead (Fig. 5f and S37,†**3d** + lamp).

## Conclusions

In summary, a new type of cyclic trimeric BODIPY array was reported from easily accessible *meso*-mesityldipyrrinone and arylboronic acids in a one-pot reaction. These cyclic trimeric BODIPYs show a close to orthogonal arrangement of the BODIPY units in their X-ray structures. The unique and rigid structures not only provide strong excitonic coupling of dyes in the singlet excited state, but also promote a long-lived triplet excited state through an efficient ISC process. These BODIPY arrays, as novel heavy-atom-free photosensitizers, showed good reactive oxygen species generation efficiencies (up to 0.72) which were contributed by both ^1^O_2_ and O_2_^−^˙ under light irradiation, and promising *in vitro* PDT results with a large PDT treatment window and high photocytotoxicity of cells at 152.1 nM.

## Data availability

The experimental, crystallographic and computational data are available in the ESI.†

## Author contributions

Zhaoyang Zhu and Xue Zhang contributed equally. Z. Zhu carried out the synthesis, the steady state optical spectroscopy studies and data analysis; X. Zhang performed the nanosecond transient absorption and singlet oxygen measurements; X. Guo performed the EPR spectral measurements and biological studies. Dr Q. Wu carried out the theoretical calculations. Z. Li and Dr C. Yu obtained the X-ray structures and part of the optical spectra. Prof. E. Hao, Prof. L. Jiao and Prof. J. Zhao carried out the data analysis, devised the project and co-wrote the manuscript.

## Conflicts of interest

There are no conflicts to declare.

## Supplementary Material

SC-012-D1SC04893G-s001

SC-012-D1SC04893G-s002
