# Supplementary material for: Orthogonally aligned cyclic BODIPY arrays with long-lived triplet excited states as efficient heavy-atom-free photosensitizers
Source: Chem Sci. 2021 Oct 29;12(44):14944–51. doi: 10.1039/d1sc04893g (PMC8597848; doi:10.1039/d1sc04893g)
Supplement: SC-012-D1SC04893G-s001 [file SC-012-D1SC04893G-s001.pdf]

Electronic Supplementary Material (ESI) for Chemical Science.
This journal is © The Royal Society of Chemistry 2021

Electronic Supporting Information for

Orthogonally Aligned Cyclic BODIPY Arrays with Long-lived Triplet Excited States as Efficient Heavy-Atom-Free Photosensitizers

Zhaoyang Zhu,[a]+ Xue Zhang,[b]+ Xing Guo,[a] Qinghua Wu,[a] Zhongxin Li,[a] Changjiang Yu,[a] Erhong Hao,[a]* Lijuan Jiao,[a]* Jianzhang Zhao[b]*

[a] Key Laboratory of Functional Molecular Solids, Ministry of Education, and School of Chemistry and Materials Science, Anhui Normal University, Wuhu 241002, China

[b] State Key Laboratory of Fine Chemicals, School of Chemical Engineering, Dalian University of Technology, Dalian 116024, China

[+] These authors contributed equally.

***E-mail:** haoehong@ahnu.edu.cn (E. Hao), jiao421@ahnu.edu.cn (L. Jiao), zhaojzh@dlut.edu.cn (J. Zhao)

Table of Contents

1. General Information S1

2. Crystallographic Data S2

3. Synthesis and Characterization S8

4. Photophysical Data S10

5. Photostabilities S13

6. Singlet Oxygen Measurements S14

7. Nanosecond Time-Resolved Transient Absorption Spectra S19

8. Theoretical Calculations S23

9. Biological Studies... .... S37

10. Scanned NMR and HRMS Spectra S47

11. Supporting References S59

1. General Information

Reagents and solvents were used as received from commercial suppliers (Energy Chemicals, Shanghai, China) unless noted otherwise. All reactions were performed in oven-dried or flame-dried glassware unless stated otherwise and were monitored by TLC using 0.25 mm silica gel plates with UV indicator (60F-254). ^{1}H and ^{13}C NMR spectra were recorded on a 400 MHz NMR spectrometer at room temperature. Chemical shifts (δ) are given in ppm relative to $CDCl_3$ (7.26 ppm for ^{1}H and 77 ppm for ^{13}C) or to internal TMS. High-resolution mass spectra (HRMS) were obtained using ESI-TOF and APCI-TOF in positive mode. The EPR spectra were obtained using a Bruker EMX plus6/1 variable-temperature X-band apparatus.

UV-visible absorption and fluorescence emission spectra were recorded on commercial spectrophotometers (Shimadzu UV-2450 and Edinburgh FS5 spectrometers). All measurements were made at 25 °C, using 5 × 10 mm cuvettes. Relative fluorescence quantum efficiencies of BODIPY derivatives were obtained by comparing the areas under the corrected emission spectrum of the test sample in various solvents with fluorescein (Φ = 0.90 in 0.1 N NaOH aqueous solution)[1]. Non-degassed, spectroscopic grade solvents and a 10 mm quartz cuvette were used. Dilute solutions (0.01<A<0.05) were used to minimize the reabsorption effects. Quantum yields were determined using the following equation[2]:

$$\Phi_X = \Phi_S (I_X/I_S) (A_S/A_X) (n_X/n_S)^2$$

Where Φ_S stands for the reported quantum yield of the standard, I stands for the integrated emission spectra, A stands for the absorbance at the excitation wavelength and *n* stands for the refractive index of the solvent being used. X subscript stands for the test sample, and S subscript stands for the standard.

Crystals of compounds **3a**, **3b** and **4** suitable for X-ray analysis were obtained *via* the slow diffusion of petroleum ether into their dichloromethane solutions. The vial containing this solution was placed, loosely capped, to promote the crystallization. A suitable crystal was chosen and mounted on a glass fiber using grease. Data were collected using a diffractometer equipped with a graphite crystal monochromator situated in the incident beam for data collection at room temperature. Cell parameters were retrieved using SMART[3] software and refined using SAINT on all observed reflections. The determination of unit cell parameters and data collections were performed with Mo *Kα* radiation (λ) at 0.71073 Å. Data reduction was performed using the SAINT software,[4] which corrects for Lp and decay. The structure was solved by the direct method using the SHELXS-97[3] program and refined by least

squares method on F^2, SHELXL-2018/3,[5] incorporated in SHELXTL V5.10.[6] CCDC-1915579 (**3a**), CCDC-1915583 (**3d**) and CCDC-1915582 (**4**) contain the supplementary crystallographic data for this paper. These data can be obtained free of charge from The Cambridge Crystallographic Data Centre *via* www.ccdc.cam.ac.uk/data_request/cif.

No unexpected or unusually high safety hazards were encountered.

2. Crystallographic Date

Table S1. Selected geometrical parameters of compound **3a**, **3b** and **4** obtained from crystallography.

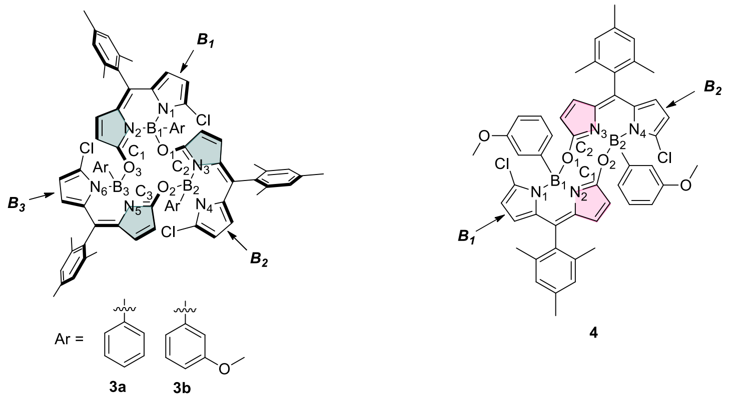

Compounds	3a	3b	4
The B_1-N_1 bond length (Å)	1.5723 (30)	1.5719 (62)	1.5719 (19)
The B_1-N_2 bond length (Å)	1.5517 (33)	1.5663 (53)	1.5694 (23)
The B_2-N_3 bond length (Å)	1.5654 (30)	1.5632 (61)	1.5694 (23)
The B_2-N_4 bond length (Å)	1.5667 (34)	1.5579 (62)	1.5719 (19)
The B_3-N_5 bond length (Å)	1.5628 (35)	1.5497 (60)	/
The B_3-N_6 bond length (Å)	1.5695 (32)	1.5632 (54)	/
The O_1-C_2 bond length (Å)	1.2964 (32)	1.3009 (57)	1.3132 (19)
The O_2-C_1 bond length (Å)	/	/	1.3132 (19)
The O_2-C_3 bond length (Å)	1.3032 (30)	1.2876 (46)	/
The O_3-C_1 bond length (Å)	1.2923 (29)	1.2910 (46)	/
The B_1-O_1 bond length (Å)	1.5113 (30)	1.4829 (55)	1.4800 (26)
The B_2-O_2 bond length (Å)	1.4948 (29)	1.4934 (54)	1.4800 (26)
The B_3-O_3 bond length (Å)	1.4998 (29)	1.4982 (58)	/
Dihedral angles of two dipyrrin cores in B_1 and B_2 (deg)	83.685 (44)	87.291 (72)	75.842 (32)
Dihedral angles of two dipyrrin cores in B_2 and B_3 (deg)	75.982 (34)	79.026 (62)	/
Dihedral angles of two dipyrrin cores in B_1 and B_3 (deg)	84.223 (44)	89.219 (76)	/

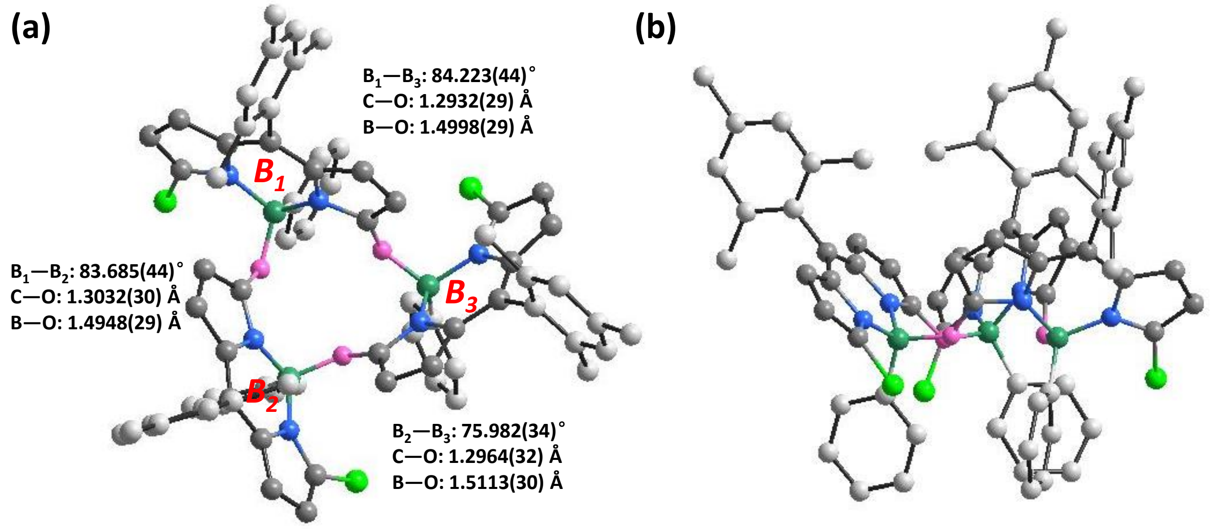

Figure S1. X-ray crystal structure of the trimeric BODIPYs **3a**. (a) top view. The dihedral angles between the three BODIPY units are 76°, 83.7° and 84.2°, respectively; (b) side view. Hydrogen atoms and solvent molecules are omitted for clarity.

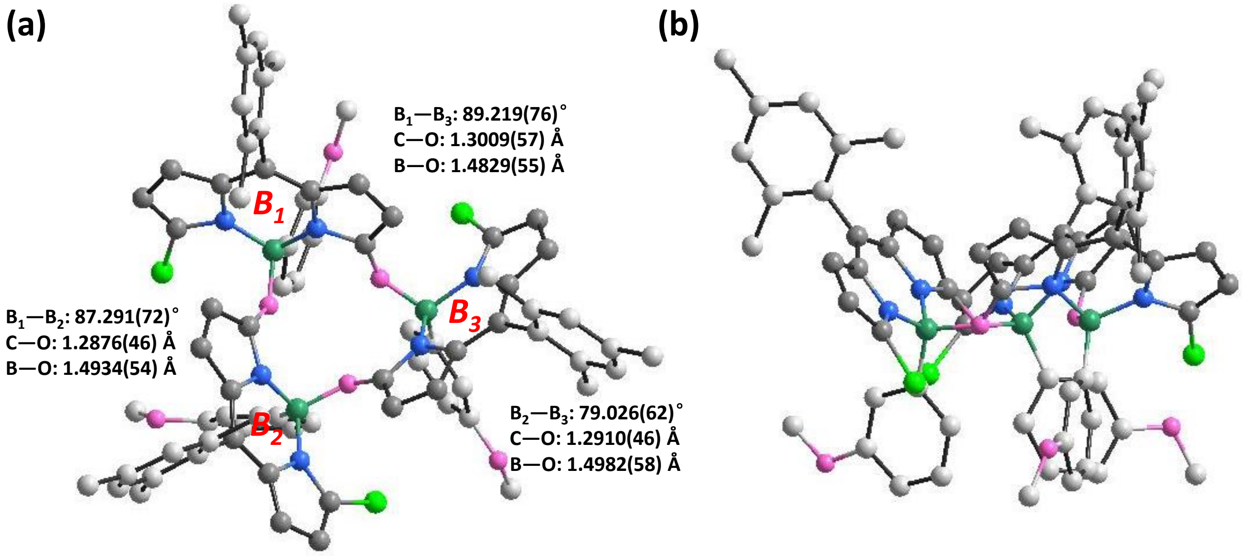

Figure S2. X-ray crystal structure of the trimeric BODIPYs **3b**. (a) top view. The dihedral angles between the three BODIPY units are 79°, 87.3° and 89.2°, dihedral angle between the two BODIPY units is almost 90° (89.2°) in both trimeric. (b) side view. Hydrogen atoms and solvent molecules are omitted for clarity.

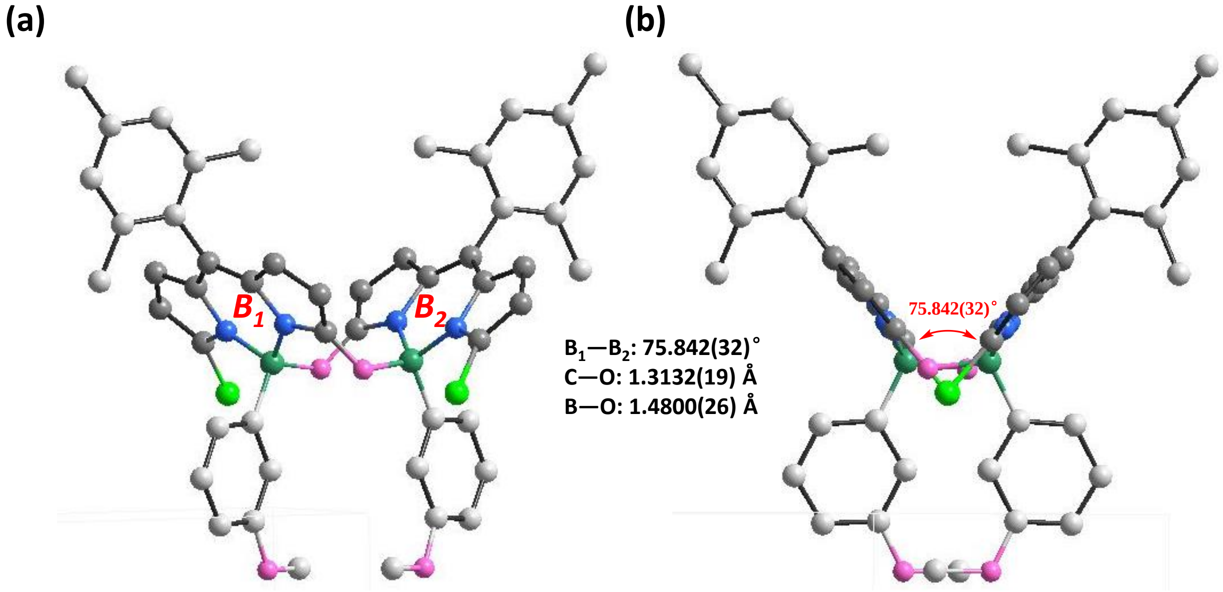

Figure S3. X-ray crystal structure of the dimeric BODIPYs **4**. (a) The dihedral angles between the two BODIPY units are 75.8°; (b) side view. Hydrogen atoms and solvent molecules are omitted for clarity.

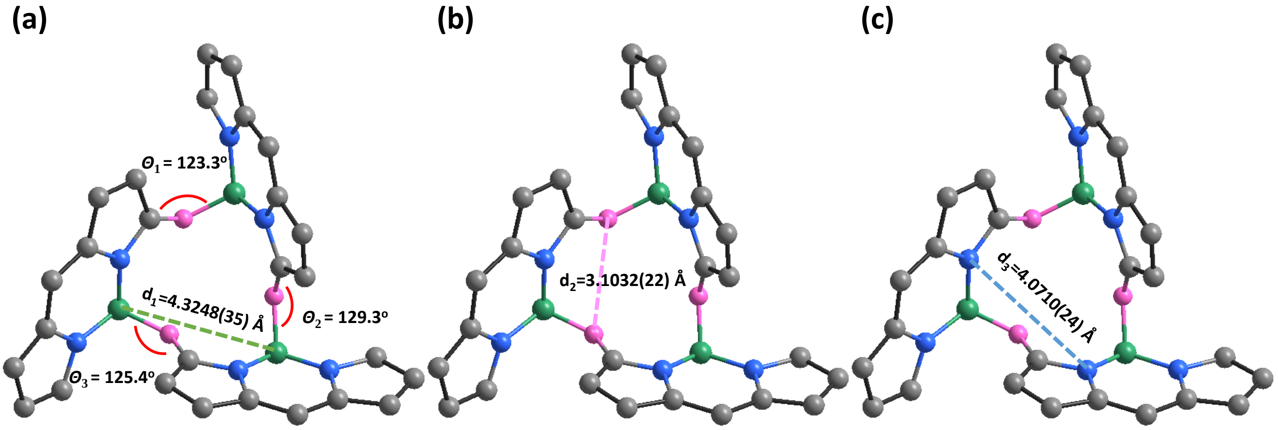

Figure S4. X-ray crystal structure of **3a** showing. (a) Minimum distance between two B-atoms of different BODIPY units. (b) Minimum distance between two O-atoms of different BODIPY units. (c) Minimum distance between two N-atoms of different BODIPY units.

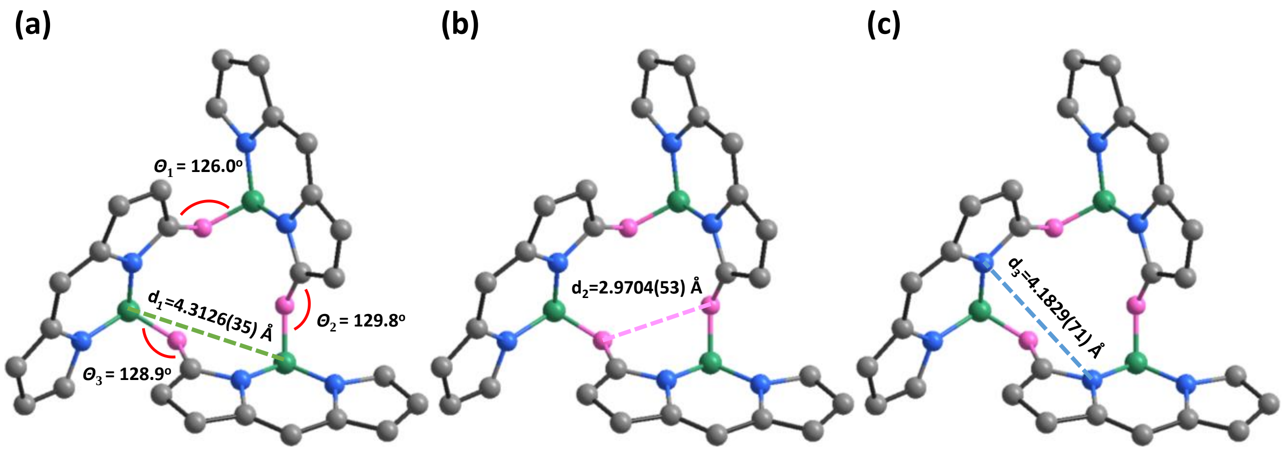

Figure S5. X-ray crystal structure of **3b** showing. (a) Minimum distance between two B-atoms of different BODIPY units. (b) Minimum distance between two O-atoms of different BODIPY units. (c) Minimum distance between two N-atoms of different BODIPY units.

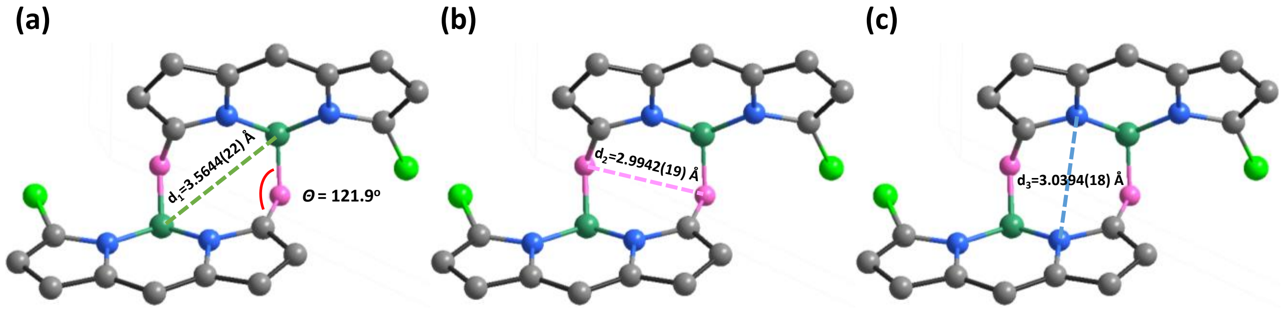

Figure S6. X-ray crystal structure of **4** showing. (a) Minimum distance between two B-atoms of different BODIPY units. (b) Minimum distance between two O-atoms of different BODIPY units. (c) Minimum distance between two N-atoms of different BODIPY units.

Table S2. Crystallographic data of **3a** and **3b**.

**Compounds**	**3a**	**3b**
CCDC	1915579	1915583
Empirical formula	$C_{72}H_{60}B_3Cl_3N_6O_3$	$C_{75}H_{66}B_3Cl_3N_6O_6$
Formula weight	1194.41	1286.12
Temperature/K	273.15	298.15
Crystal system	triclinic	monoclinic
Space group	P-1	$P2_1/n$
a/Å	12.2187(5)	15.79(3)
b/Å	13.6971(5)	18.68(3)
c/Å	20.7232(8)	25.84(5)
α/°	102.574(2)	90
β/°	90.075(2)	93.04(16)
γ/°	90.745(2)	90
Volume/$Å^3$	3384.8(2)	7612(24)
Z	44	4
ρcalcg/cm^3	1.590	1.122
μ/mm^{-1}	0.932	0.172
F(000)	1592.0	3608.0
Crystal size/mm^3	0.17 × 0.20 × 0.21	0.24 × 0.23 × 0.21
Radiation	MoKα (λ = 0.71073)	MoKα (λ = 0.71073)
2Θ range for data collection/°	5.556 to 55.124	5.706 to 55.52
Index ranges	-15 ≤ h ≤ 15, -17 ≤ k ≤ 17, -26 ≤ l ≤ 26	-20 ≤ h ≤ 20, -24 ≤ k ≤ 24, -33 ≤ l ≤ 33
Reflections collected	140792	307537
Independent reflections	15543 [R_{int} = 0.0638, R_{sigma} = 0.0356]	17602 [R_{int} = 0.2293, R_{sigma} = 0.0947]
Data/restraints/parameters	15543/0/803	17602/0/850
Goodness-of-fit on F^2	1.715	1.088
Final R indexes [I>=2σ (I)]	R_1 = 0.1224, wR_2 = 0.3463	R_1 = 0.1040, wR_2 = 0.1890
Final R indexes [all data]	R_1 = 0.1697, wR_2 = 0.3693	R_1 = 0.1956, wR_2 = 0.2238
Largest diff. peak/hole / e $Å^{-3}$	2.99/-0.48	0.56/-0.29

Table S3. Crystallographic data of **4**.

**Compound**	**4**
CCDC	1915582
Empirical formula	$C_{50}H_{44}B_2Cl_2N_4O_4$
Formula weight	857.41
Temperature/K	300.02
Crystal system	monoclinic
Space group	C2/c
a/Å	24.9180(13)
b/Å	13.8086(6)
c/Å	14.4975(5)
α/°	90
β/°	114.175(3)
γ/°	90
Volume/Å^3	4550.9(4)
Z	4
ρ_{calc}g/cm^3	1.251
μ/mm^{-1}	0.192
F(000)	1792.0
Crystal size/mm^3	0.24 × 0.23 × 0.21
Radiation	MoKα (λ = 0.71073)
2Θ range for data collection/°	5.9 to 55.074
Index ranges	-32 ≤ h ≤ 32, -17 ≤ k ≤ 17, -18 ≤ l ≤ 18
Reflections collected	93718
Independent reflections	5232 [R_{int} = 0.0465, R_{sigma} = 0.0170]
Data/restraints/parameters	5232/0/284
Goodness-of-fit on F^2	1.036
Final R indexes [I>=2σ (I)]	R_1 = 0.0477, wR_2 = 0.1181
Final R indexes [all data]	R_1 = 0.0658, wR_2 = 0.1307
Largest diff. peak/hole / e Å^{-3}	0.26/-0.32

3. Synthesis and characterization

Compound **1** were synthesized according to literature.[7-8]

Synthesis of compound 2: To compound **1** (994 mg, 3 mmol) in 40 mL DMF was added 0.9 mL TFA (12 mmol). The reaction mixture was left stirred at room temperature for 36 h before pouring into 50 mL water and extracted with CH_2Cl_2 (3 × 30 mL). Organic layers were combined and dried with anhydrous Na_2SO_4. Solvent was removed under vacuum. The crude product was further purified by column chromatography on silica gel using a mixture of ethyl acetate and hexane as the eluent to give the desired **2** as yellow solid in 66% yield (620 mg). ^{1}H NMR (400 MHz, $CDCl_3$) δ 10.69 (s, 1H), 9.99 (s, 1H), 6.93 (s, 2H), 6.57 (d, J = 5.0 Hz, 1H), 6.07 (d, J = 5.5 Hz, 3H), 2.35 (s, 3H), 2.08 (s, 6H); ^{13}C NMR (101 MHz, $CDCl_3$) δ 174.3, 138.2, 138.0, 137.8, 132.3, 130.4, 128.3, 127.9, 120.9, 120.7, 119.7, 116.6, 116.5, 109.3, 21.1, 19.8. HRMS (APCI) m/z calcd. for $C_{18}H_{18}N_2O$ $[M+H]^+$: 313.1102, found 313.1092.

Using compound 3a as an example to show the general procedure for the synthesis of cyclic trimeric BODIPYs 3: To compound **2** (125 mg, 0.4 mmol) in 5 mL toluene was added phenylboronic acid (146 mg, 1.2 mmol), followed by DIPEA (66 μL, 0.4 mmol). The reaction mixture was left refluxing at 110 °C for 3 h. Upon the completion of the reaction, the reaction mixture was cooled down to room temperature. The reaction mixture was poured into 50 mL water and extracted with CH_2Cl_2 (3 × 30 mL). Organic layers were combined and dried with anhydrous Na_2SO_4. Solvent was removed under vacuum. The crude product was further purified by column chromatography on silica gel using a mixture of ethyl acetate and hexane as the eluent to give the desired **3a** as an orange solid in 66% yield (105 mg). ^{1}H NMR (400 MHz, $CDCl_3$) δ 7.32 – 7.25 (m, 3H), 7.22 (t, J = 7.5 Hz, 6H), 6.93 – 6.83 (m, 12H), 6.54 (d, J = 5.0 Hz, 3H), 6.17 (d, J = 3.9 Hz, 3H), 5.99 (d, J = 3.9 Hz, 3H), 4.85 (d, J = 5.0 Hz, 3H), 2.32 (s, 9H), 2.19 (s, 9H), 2.01 (s, 9H); ^{13}C NMR (101 MHz, $CDCl_3$) δ 170.2, 138.3, 138.3, 137.5, 136.5, 134.1, 133.8, 133.6, 133.3, 130.5, 130.2, 129.3, 128.2, 128.0, 126.4, 126.3, 120.5, 113.9 112.2, 21.1, 20.0, 20.0. HRMS (ESI) m/z calcd for $C_{72}H_{60}B_3Cl_3N_6O_3Na^+$ $[M+Na]^+$: 1219.3980, found 1219.3995.

Compound **3b** was obtained as an orange solid in 68% yield (116 mg) with **4** as a less polar orange solid in 12% yield (21 mg) from the reaction of compound **2** (125 mg, 0.4 mmol) and 3-methoylphenylboronic acid (182 mg, 1.2 mmol) in the presence of DIPEA (66 μL, 0.4 mmol). The ratio of the yields for these two products can be tuned by simple reducing the amount of DIPEA (33 μL, 0.2 mmol) added into the system. In this case, the yield for **3d** was dropped to 44% yield (75 mg), while the yield for was increased to **4** 28% (48 mg). Compound **3b**: ^{1}H NMR (400 MHz, $CDCl_3$) δ

7.19 (t, J = 7.7 Hz, 3H), 6.92 – 6.84 (m, 9H), 6.59 (d, J = 1.1 Hz, 3H), 6.55 – 6.53 (m, 3H), 6.46 (d, J = 7.3 Hz, 3H), 6.17 (d, J = 3.9 Hz, 3H), 6.00 (d, J = 3.9 Hz, 3H), 4.85 (d, J = 5.0 Hz, 3H), 3.70 (s, 9H), 2.32 (s, 9H), 2.19 (s, 9H), 2.01 (s, 9H); ^{13}C NMR (101 MHz, $CDCl_3$) δ 170.2, 158.3, 138.3, 137.4, 136.5, 134.0, 133.7, 133.0, 130.4, 130.1, 129.3, 128.2, 128.0, 127.9, 127.5, 120.6, 116.6, 116.6, 114.0, 112.3, 111.8, 54.8, 21.1, 19.9(7), 19.9(5). HRMS (ESI) m/z calcd for $C_{75}H_{66}B_3Cl_3N_6O_6Na^+$ $[M+Na]^+$: 1307.4281, found 1307.4303.

Compound **4**: ^{1}H NMR (400 MHz, $CDCl_3$) δ 7.21 – 7.18 (m, 4H), 7.09 (d, J = 7.3 Hz, 2H), 6.96 (s, 2H), 6.88 (s, 2H), 6.83 – 6.80 (m, 2H), 6.61 (d, J = 4.7 Hz, 2H), 6.28 (d, J = 4.0 Hz, 2H), 6.15 (d, J = 4.0 Hz, 2H), 5.85 (d, J = 4.7 Hz, 2H), 3.78 (s, 6H), 2.34 (s, 6H), 2.27 (s, 6H), 1.75 (s, 6H); ^{13}C NMR (101 MHz, $CDCl_3$). δ 171.9, 158.7, 138.5, 138.3, 136.8, 136.5, 135.7, 134.3, 133.1, 132.0, 130.0, 129.0, 128.2, 128.10, 128.0, 124.5, 122.9, 116.9, 114.9, 114.5, 113.1, 55.0, 21.1, 20.0, 19.0. HRMS (ESI) m/z calcd for $C_{50}H_{45}B_2Cl_2N_4O_4^+$ $[M+H]^+$: 857.2999, found 857.3004.

Compound **3c** was obtained as an orange solid in 49% yield (85 mg) from the reaction of compound **2** (125 mg, 0.4 mmol) and 3,5-difluorophenylboronic acid (190 mg, 1.2 mmol) in the presence of DIPEA (66 µL, 0.4 mmol). ^{1}H NMR (400 MHz, $CDCl_3$) δ 6.92 (d, J = 17.8 Hz, 6H), 6.80 (s, 3H), 6.59 (d, J = 5.0 Hz, 3H), 6.36 (d, J = 8.8 Hz, 6H), 6.25 (d, J = 3.9 Hz, 3H), 6.08 (d, J = 3.9 Hz, 3H), 4.85 (d, J = 5.0 Hz, 3H), 2.33 (s, 9H), 2.18 (s, 9H), 2.00 (s, 9H); ^{13}C NMR (101 MHz, $CDCl_3$) δ 169.7, 163.7, 138.6, 137.3, 136.3, 134.9, 134.6, 130.3, 130.2, 128.7, 128.4, 128.1, 122.2, 121.7, 114.7, 114.5, 111.7, 102.2, 21.1, 20.0, 19.9(9). HRMS (ESI) m/z calcd for $C_{72}H_{55}B_3Cl_3F_6N_6O_3^+$ $[M+H]^+$: 1303.3579, found 1303.3583.

Compound 3d was obtained as an orange solid in 57% yield (102 mg) from the reaction of compound **2** (125 mg, 0.4 mmol) and 2-naphthaleneboronic acid (206 mg, 1.2 mmol). ^{1}H NMR (400 MHz, $CDCl_3$) δ 7.83 (d, J = 8.1 Hz, 3H), 7.44 – 7.42 (m, 6H), 7.25 (d, J = 5.3 Hz, 6H), 7.13 (s, 3H), 6.93 (d, J = 13.7 Hz, 9H), 6.62 (d, J = 5.0 Hz, 3H), 6.18 (d, J = 3.9 Hz, 3H), 5.95 (d, J = 3.9 Hz, 3H), 4.95 (d, J = 5.0 Hz, 3H), 2.33 (s, 9H), 2.24 (s, 9H), 2.06 (s, 9H); ^{13}C NMR (101 MHz, $CDCl_3$) δ 170.4, 138.4, 138.3, 137.5, 136.5, 136.5, 134.2, 134.0, 133.1, 132.9, 132.8, 132.6, 130.6, 130.2, 129.3, 129.2, 128.9, 128.3, 128.3, 128.0, 127.3, 125.4, 124.7, 124.7, 124.4, 124.3, 120.8, 114.0, 112.2, 21.1, 20.0. HRMS (ESI) m/z calcd for $C_{84}H_{66}B_3Cl_3N_6O_3Na^+$ $[M+Na]^+$: 1369.4455, found 1369.4454.

4. Photophysical Data

Table S4. Photophysical properties of **3a-d** and **4** in different solvents at room temperature.

dyes	solvents	λ_{abs}^{max}(nm)	λ_{em}^{max}(nm)	$lg\varepsilon_{max}^{c}$	Φ_F^d	Stokes-shift (cm^{-1})
**3a**	CH_2Cl_2	$467^a/493^b$	523	4.94	0.0009	1164
	Hexane	470/497	520	4.93	0.0010	890
	Toluene	471/498	523	4.94	0.0013	960
	THF	467/494	520	4.94	0.0002	1012
	MeCN	463	518	4.94	0.0005	2293
**3b**	CH_2Cl_2	468/494	523	4.91	0.0011	2247
	Hexane	470/497	521	4.98	0.0010	2083
	Toluene	472/498	525	5.00	0.0003	2139
	THF	469/495	520	5.01	0.0002	2091
	MeCN	464	519	4.95	0.0015	2284
**3c**	CH_2Cl_2	471/496	526	4.82	0.0011	2220
	Hexane	472/499	520	4.89	0.0010	1956
	Toluene	474/500	525	4.91	0.0007	2049
	THF	470/496	526	4.92	0.0008	2265
	MeCN	466	517	4.86	0.0016	2117
**3d**	CH_2Cl_2	468/494	521	5.16	0.0003	1049
	Hexane	471/497	519	5.15	0.0014	853
	Toluene	473/500	523	5.15	0.0011	880
	THF	469/495	520	5.15	0.0011	971
	MeCN	464	516	5.15	0.0004	2172
**4**	CH_2Cl_2	472/497	515	4.49	0.0021	1769
	Hexane	474/498	517	4.51	0.0018	1755
	Toluene	473/496	515	4.42	0.0009	1724
	THF	471/496	517	4.52	0.0011	1889
	MeCN	470/493	516	4.46	0.0016	1897

[a]maximum absorption peak; [b]shoulder peak; [c]molar absorption coefficients are corresponding to the maximum absorption peak; [d]fluorescence quantum yields were obtained by using Fluorescein (Φ_F = 0.90 in 0.1 M NaOH) as reference. The standard errors are less than 10% based on three measurements.

Overlaid absorption and fluorescence emission spectra of dyes 3 and 4 in different solvents at room temperature.

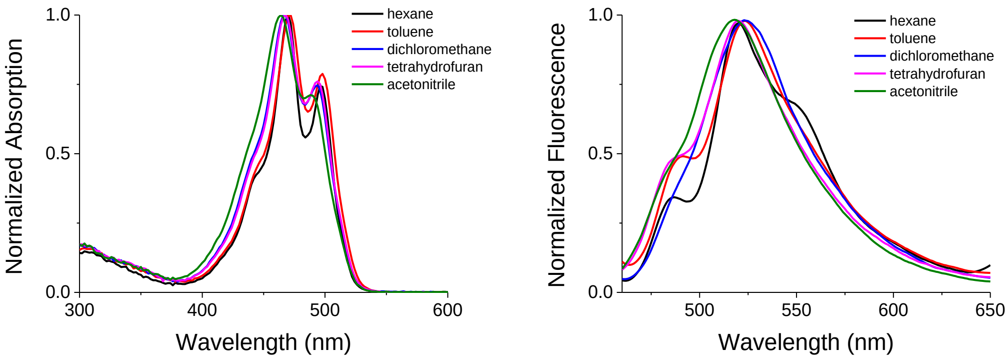

Figure S7. Normalized absorption (left) and emission (right) spectra of **3a** recorded in different solvents, excited at 450 nm.

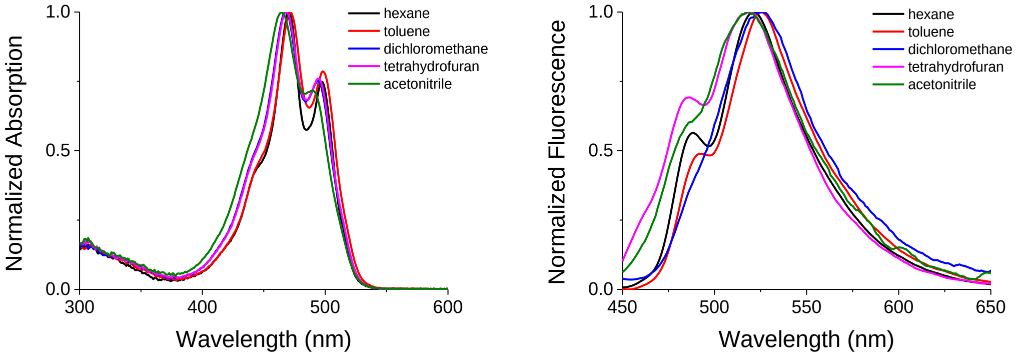

Figure S8. Normalized absorption (left) and emission (right) spectra of **3b** recorded in different solvents, excited at 450 nm.

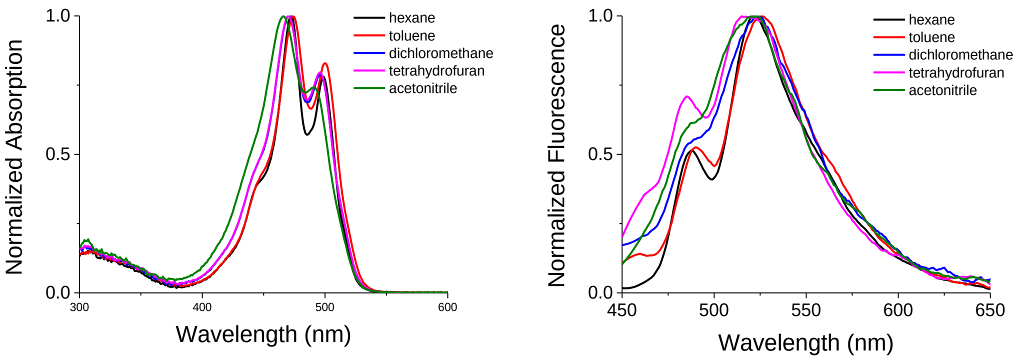

Figure S9. Normalized absorption (left) and emission (right) spectra of **3c** recorded in different solvents, excited at 450 nm.

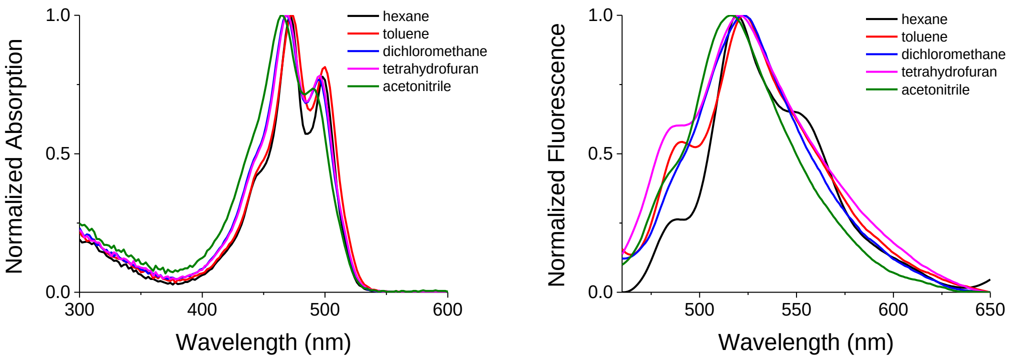

Figure S10. Normalized absorption (left) and emission (right) spectra of **3d** recorded in different solvents, excited at 450 nm.

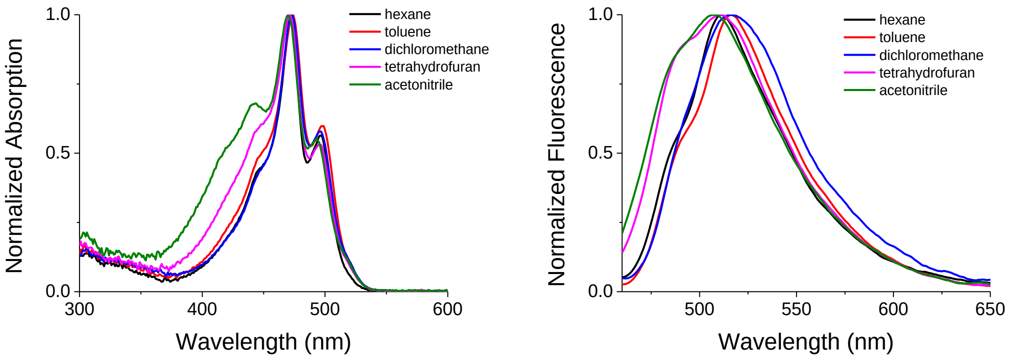

Figure S11. Normalized absorption (left) and emission (right) spectra of **4** recorded in different solvents, excited at 450 nm.

5. Photostabilities

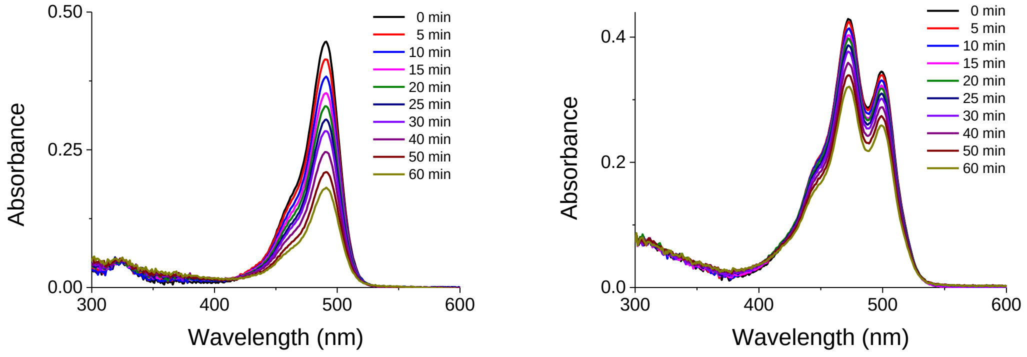

Figure S12. Absorbance changes of Fluorescein in 0.1 M NaOH (left) and trimeric BODIPYs **3d** in toluene (right).

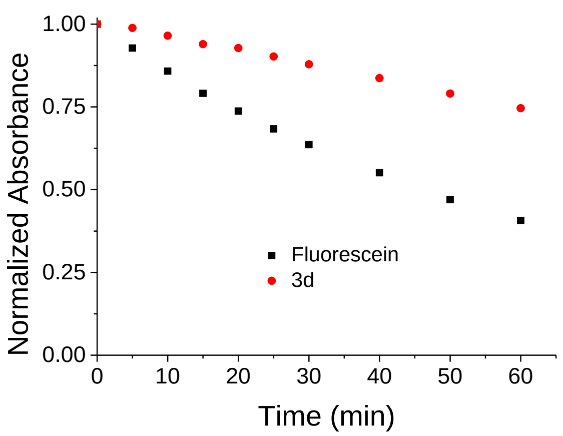

Figure S13. Absorbance changes (at 491 nm) of Fluorescein (2×10^{-5} mol/L) in 0.1 M NaOH and our trimeric BODIPYs **3d** (2×10^{-5} mol/L) in toluene under strong continuous irradiation with a 50 W white LED lamp over 1 h.

6. Singlet Oxygen Measurements

Table S5. Singlet Oxygen Quantum Yields (Φ_Δ) of **3a-d** in Different Solvents [a]

dyes	*n*-Hexane [b]	Toluene [c]	THF [d]	DCM [e]	ACN [f]
**3a**	3.8	31.0	18.2	34.5	10.8
**3b**	– [g]	27.8	47.1	71.7	– [g]
**3c**	– [g]	28.3	25.7	34.0	0.8
**3d**	6.4	36.1	27.8	41.2	12.1

[a] In percentage, λ_{ex} = 490 nm. **RB** was used as standard compound, Φ_Δ = 80% in Methanol, [b] n-Hexane, $E_T(30)$ = 30.9; [c] Toluene, $E_T(30)$ = 33.9; [d] Tetrahydrofuran, $E_T(30)$ = 37.4; [e] Dichloromethane, $E_T(30)$ = 41.1; [f] Acetonitrile, $E_T(30)$ =46.0; [g] Not observed.

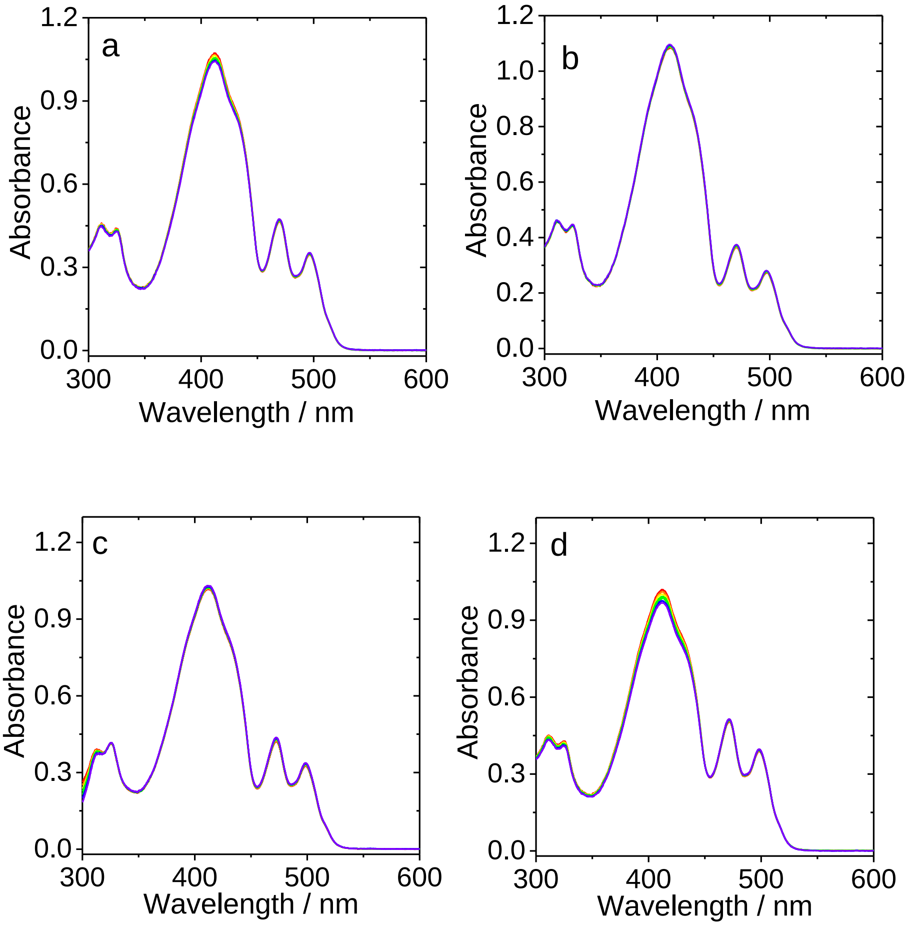

Figure S14. Changes in the absorption spectrum of **DPBF** upon irradiation in the presence of (a) **3a**, (b) **3b**, (c) **3c** and (d) **3d** in n-Hexane (recorded at 30 s intervals).

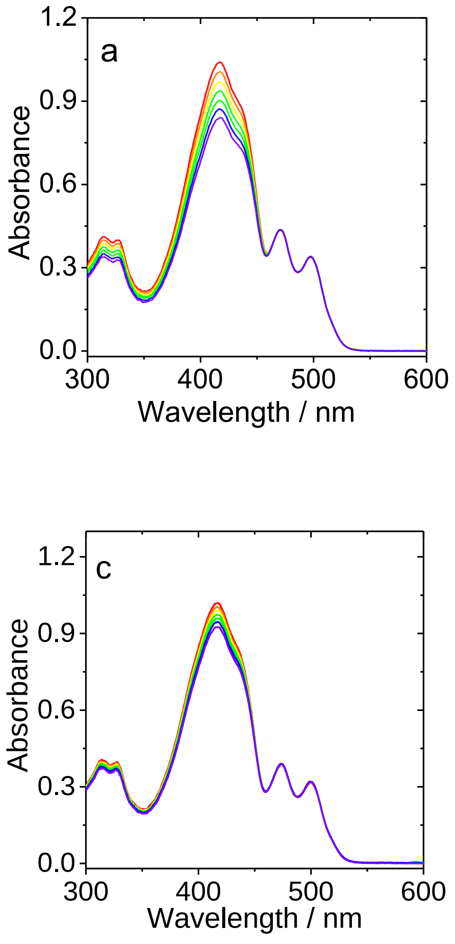

Figure S15. Changes in the absorption spectrum of **DPBF** upon irradiation in the presence of (a) **3a**, (b) **3b**, (c) **3c** and (d) **3d** in toluene (recorded at 30 s intervals).

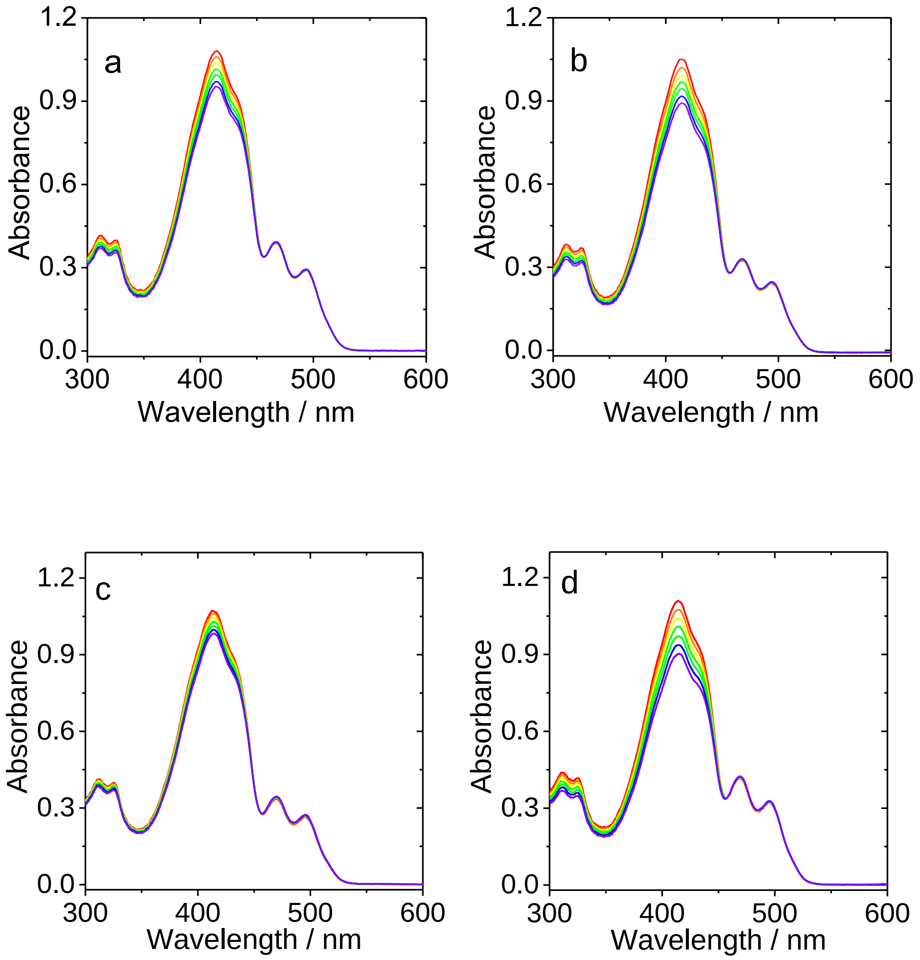

Figure S16. Changes in the absorption spectrum of **DPBF** upon irradiation in the presence of (a) **3a**, (b) **3b**, (c) **3c** and (d) **3d** in THF (recorded at 30 s intervals).

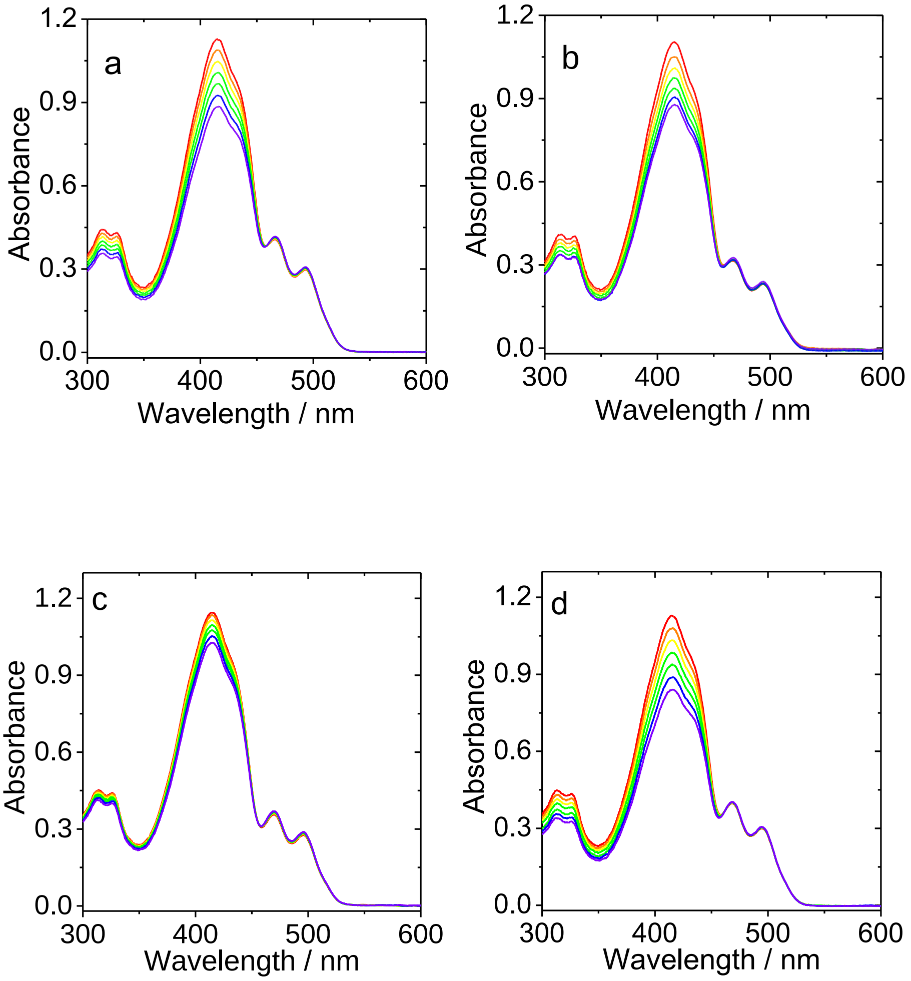

Figure S17. Changes in the absorption spectrum of **DPBF** upon irradiation in the presence of (a) **3a**, (b) **3b**, (c) **3c** and (d) **3d** in DCM (recorded at 30 s intervals).

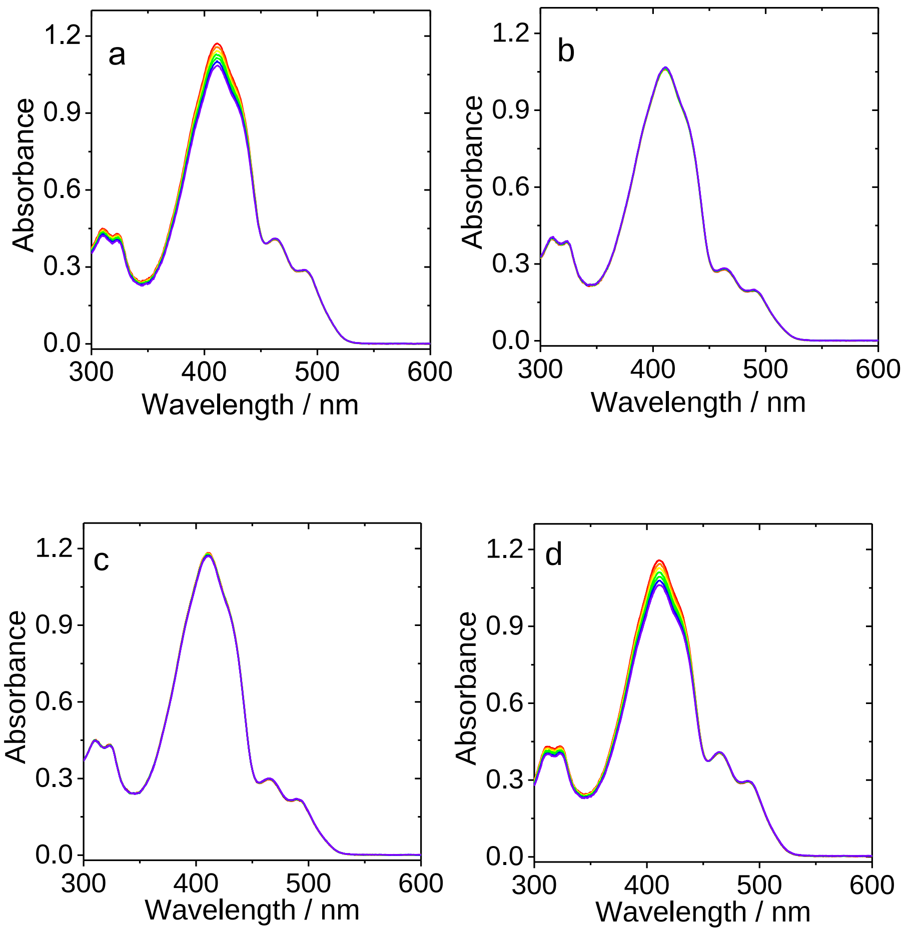

Figure S18. Changes in the absorption spectrum of **DPBF** upon irradiation in the presence of (a) **3a**, (b) **3b**, (c) **3c** and (d) **3d** in Acetonitrile (recorded at 30 s intervals).

7. Nanosecond Time-Resolved Transient Absorption Spectra

Table S6. Singlet oxygen quantum yields and triplet lifetime of **3a** and **3d** in different solvents.

Dyes	solvents	Φ_Δ[a]	τ_T[b]
**3a**	Hexane	0.04	-[c]
	Toluene	0.31	195.8
	DCM	0.35	257.5
	THF	0.18	237.3
	Acetonitrile	0.11	-
**3d**	Hexane	0.06	-
	Toluene	0.36	179.9
	DCM	0.41	188.9
	THF	0.28	271.6
	Acetonitrile	0.12	-

[a]Singlet oxygen quantum yield with **RB** (Φ_Δ = 0.80 in Methanol) as the standard; determination error = ±0.03. [b]Triplet state lifetime, in μs. [c] Not determined.

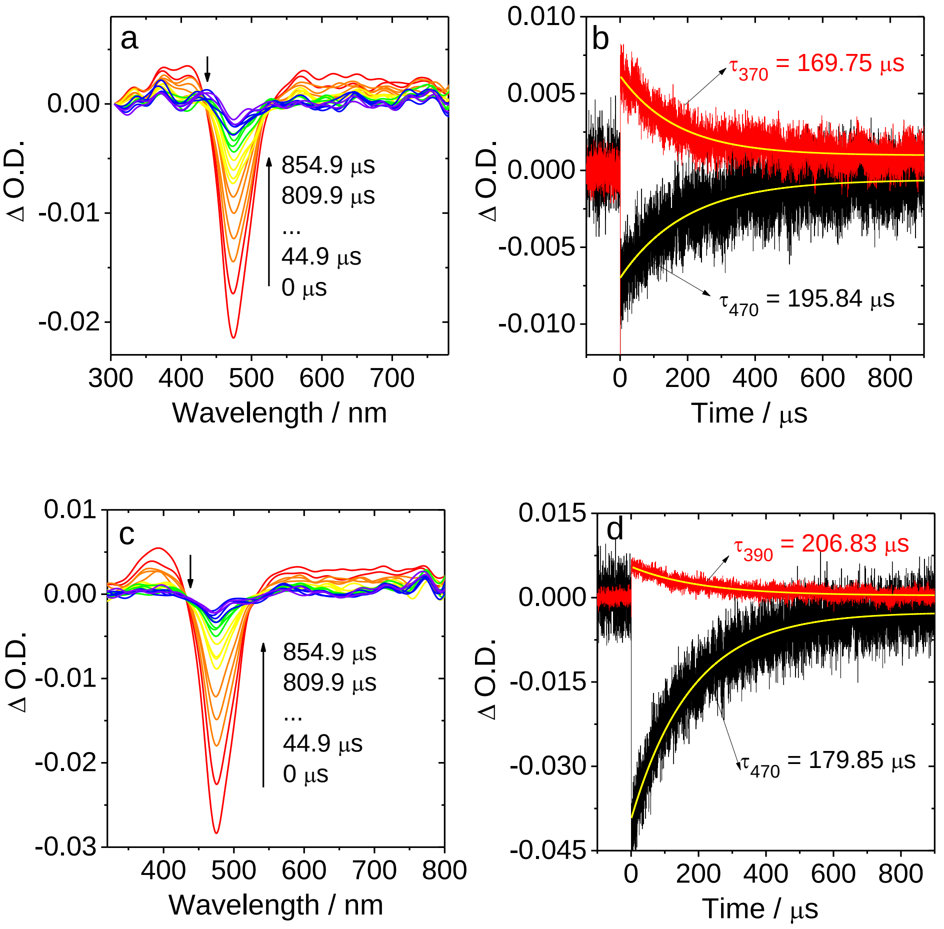

Figure S19. Nanosecond transient absorption spectra of **3a** (a) and the decay trace (b); **3d** (c) and the decay trace (d). $c = 1.0 \times 10^{-5}$ M. $\lambda_{ex} = 490$ nm, in deaerated toluene, 20 °C.

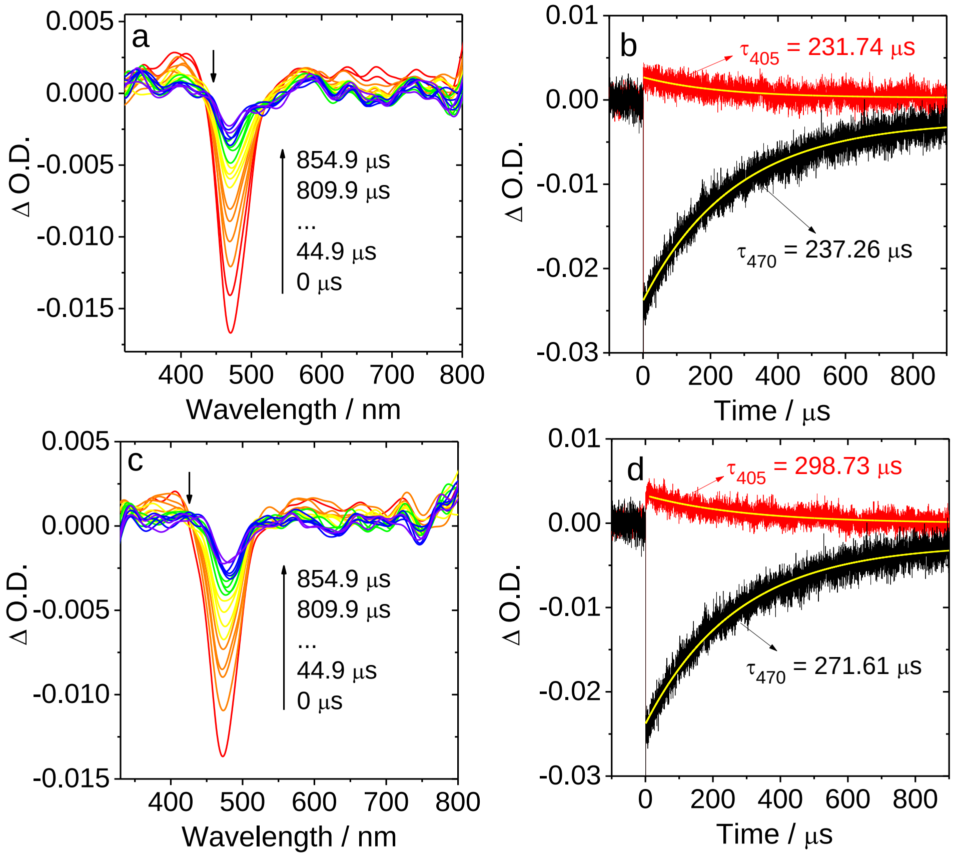

Figure S20. Nanosecond transient absorption spectra of **3a** (a) and the decay trace (b); **3d** (c) and the decay trace (d). $c = 1.0 \times 10^{-5}$ M. λ_{ex} = 490 nm, in deaerated THF, 20 °C.

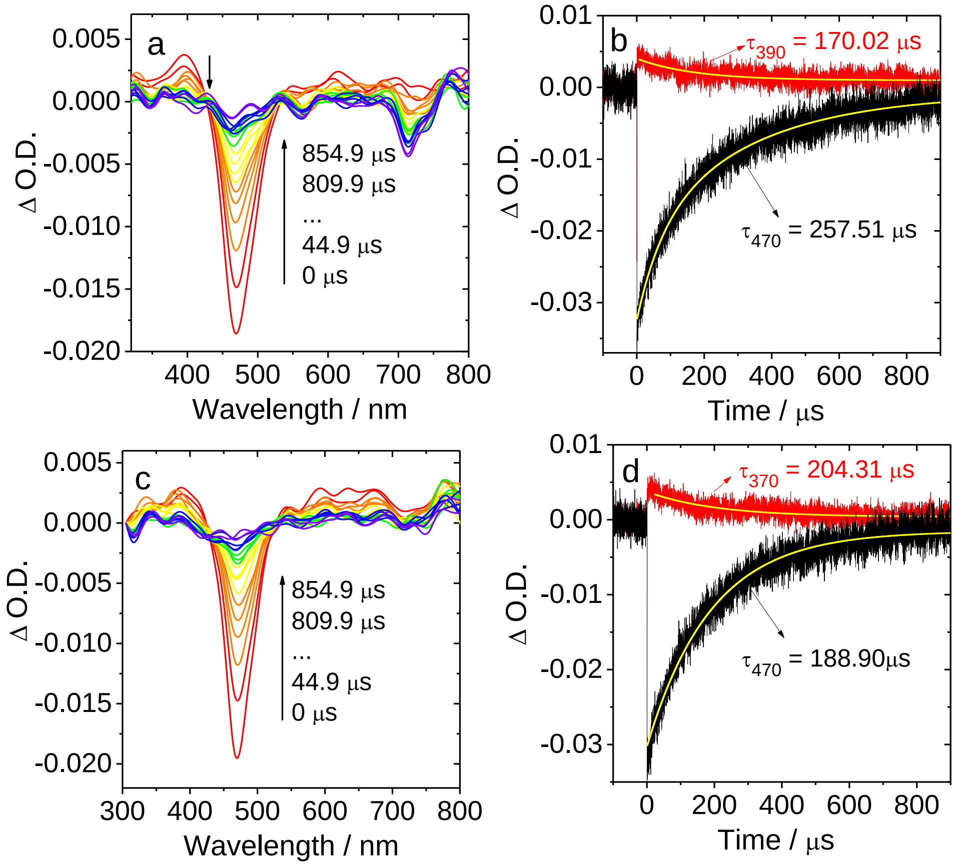

Figure S21. Nanosecond transient absorption spectra of **3a** (a) and the decay trace (b); **3d** (c) and the decay trace (d). $c = 1.0 \times 10^{-5}$ M. $\lambda_{ex} = 490$ nm, in deaerated DCM, 20 °C.

8. Theoretical Calculations

The ground state geometry was optimized by using DFT method at B3LYP/6-31G(d) level. The same method was used for vibrational analysis to verify that the optimized structures correspond to local minima on the energy surface. TD-DFT computations were used the optimized ground state geometries under the B3LYP/6-31G (d, p) theoretical level. The calculated molecules in dichloromethane were done using the Self-Consistent Reaction Field (SCRF) method and Polarizable Continuum Model (PCM). The geometries of the lowest singlet (S1) excited states were also optimized at the TD-B3LYP/6-31G(d) level of theory. All of the calculations were carried out by the methods implemented in Gaussian 09 package.[9a] SOCs were computed using the quadratic-response TDDFT approach,[9b] i.e. QR-TD-DFT, as implemented in the Dalton program[9c] at their S1 optimized geometries. The SOC operator makes use of a semi-empirical effective single-electron approximation.[9d] For the latter calculations the B3LYP functional in combination to the 6-31G(d) basis set was used.

8.1 Theoretical calculations of 3

In order to calculate the spin-orbit coupling (SOC) properties, both *meso*-mesityl group and the boron substituted aryl group were removed from the parent cyclic trimeric BODIPY for simplicity, which named compound **3** here.

Table S7. Calculated electronic transition energies and oscillator strength of **3**.

Energy State	**3** (eV)	λ (nm)	f^{a}
S1	2.48	500.81	0.0003
S2	2.49	497.96	0.0006
S3	2.49	497.74	0.0006
S4	2.53	489.87	0.0027
S5	2.53	489.70	0.0028
S6	2.55	487.16	0
S7	2.86	433.82	0.1132
S8	2.96	418.97	0.9752
S9	2.96	418.91	0.9731
S10	3.87	320.36	0.0018
T1	1.53	811.42	
T2	1.53	810.95	
T3	1.53	810.81	
T4	2.48	500.81	
T5	2.49	498.55	
T6	2.49	498.37	
T7	2.53	490.83	
T8	2.53	490.68	
T9	2.54	488.13	
T10	3.11	398.79	

[a]Oscillator strength

Table S8. Calculated SOC values of **3**.

	dye
$\langle S_1 \vert \hat{H}_{SO} \vert T_1 \rangle^a$	(0.11; -0.69; -0.25)
$\langle S_1 \vert \hat{H}_{SO} \vert T_2 \rangle$	(0.62; -0.11; -0.54)
$\langle S_1 \vert \hat{H}_{SO} \vert T_3 \rangle$	(-0.25; 0.22; 1.54)
$\langle S_1 \vert \hat{H}_{SO} \vert T_4 \rangle$	(0.08; -0.01; 0.18)

[a]Values are shown as (x component; y component; z component) and were obtained at the QR-TD-DFT/6-31G* level of theory at the S1 optimized geometry.

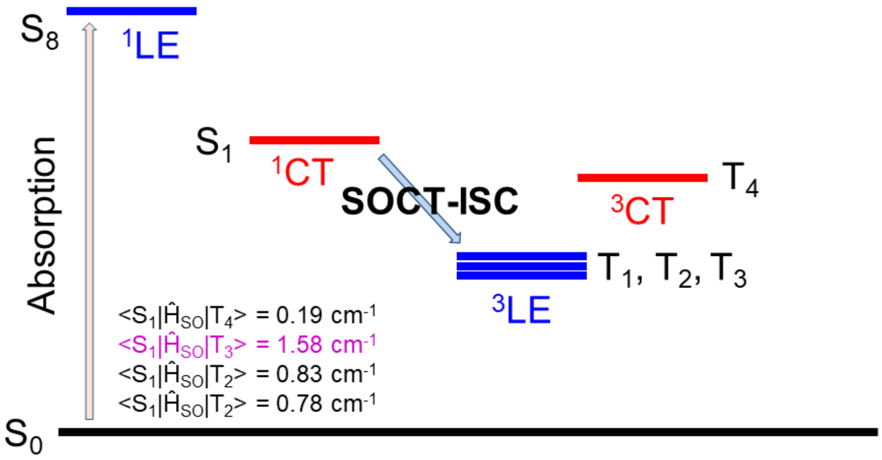

Figure S22. Schematic of different ISC mechanisms for **3**. The calculated SOC values are shown.

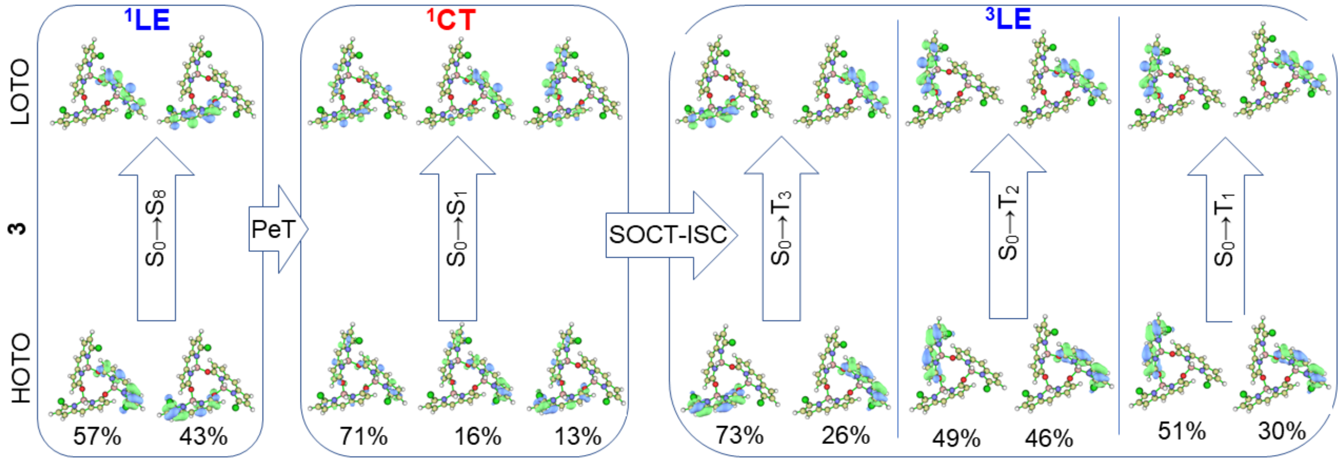

Figure S23. Calculated natural transition orbitals (NTOs) and ISC process of **3**.

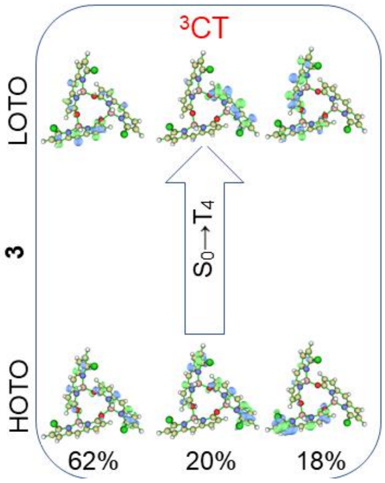

Figure S24. Calculated natural transition orbitals (NTOs) for the T_4 state of **3**.

Optimized S_0 state Geometry of 3.

Cl	3.88657900	-2.71032900	2.41912500
Cl	0.42249100	4.71366000	2.41275600
O	1.30917300	-1.23988700	0.70647700
O	0.41782600	1.74971500	0.70496100
O	-1.72624600	-0.51451400	0.70735400
N	3.81877200	-1.01942300	0.31492400
N	2.27024500	0.83702200	-0.28833500
N	-1.02402700	3.81518500	0.31738700
N	-1.86462000	1.54760000	-0.28265000
N	-2.79221400	-2.79593700	0.31825100
N	-0.40917400	-2.38788800	-0.28436400
C	4.47159800	-2.02002400	0.95178100
C	5.63025500	-2.39310600	0.26228500
H	6.31943000	-3.16746300	0.56605900
C	5.69372300	-1.56373600	-0.86062400
H	6.46217300	-1.55185300	-1.62272200
C	4.57515000	-0.71310100	-0.81982500
C	4.17547900	0.32926700	-1.68090900
C	3.05793800	1.08140000	-1.42363800
C	2.48607000	2.18715200	-2.12738900
H	2.88693100	2.61035900	-3.03976200
C	1.38103800	2.60298000	-1.43169200
H	0.71912600	3.42301300	-1.66318200
C	1.26736400	1.74444200	-0.27404500
C	-0.47744600	4.87920200	0.95172700
C	-0.73574400	6.07027400	0.26494100
H	-0.40619900	7.05346500	0.56767900
C	-1.49359300	5.71300400	-0.85349500
H	-1.89149700	6.37403700	-1.61253900
C	-1.67301600	4.31924100	-0.81308400
C	-2.38156900	3.45361200	-1.67118300
C	-2.47432000	2.10944100	-1.41480800
C	-3.15060000	1.06287900	-2.11649200
H	-3.72124600	1.19980800	-3.02635100
C	-2.95680700	-0.10289100	-1.42263100
H	-3.33834100	-1.08538600	-1.65379500
C	-2.15075700	0.22568900	-0.26831300
C	-3.98616900	-2.85664500	0.95386100
C	-4.89068800	-3.67067600	0.26377700
H	-5.90691800	-3.87681600	0.56653300
C	-4.20444500	-4.14325500	-0.85811800
C	-2.90689600	-3.60388500	-0.81635300
C	-1.80426500	-3.78202600	-1.67668300

C	-0.59294400	-3.19248600	-1.41934200
C	0.65062000	-3.25307500	-2.12294700
H	0.81557100	-3.81242200	-3.03518500
C	1.56444200	-2.50477000	-1.42803900
H	2.60558100	-2.34292800	-1.66042800
C	0.87874100	-1.97494100	-0.27071300
B	2.49127600	-0.30893500	0.74057500
B	-0.97806900	2.31041800	0.74355000
B	-1.51199100	-2.00380200	0.74372000
H	-4.57978900	-4.81388200	-1.62022600
Cl	-4.29006800	-2.00117400	2.41938400
H	-1.92083800	-4.40713100	-2.55638500
H	4.77506800	0.54188200	-2.56039600
H	-2.86922900	3.86771600	-2.54809600
H	-1.35938300	2.17128900	1.86916300
H	-1.19954800	-2.26510400	1.86880500
H	2.56217000	0.09412100	1.86497800

SCF done: -3049.30212153 Hartree

No imaginary Frequency.

Optimized S_1 state Geometry of **3**.

Cl	3.10626300	-3.70062200	2.30670900
Cl	1.57084500	4.38523900	2.47025500
O	0.98261700	-1.62745800	0.70467700
O	0.77406700	1.52914800	0.66847000
O	-1.83161200	-0.05760700	0.70033800
N	3.46905100	-1.95134900	0.32072200
N	2.39112100	0.21784800	-0.26621800
N	-0.07104600	3.94099800	0.36477500
N	-1.49474800	2.00616300	-0.23375200
N	-3.38244500	-2.05598000	0.29425500
N	-1.00659700	-2.16311500	-0.40033200
C	3.85715300	-3.08283400	0.91309800
C	4.96844500	-3.68505400	0.23335200
H	5.44997900	-4.60419700	0.53144100
C	5.25649000	-2.86155700	-0.82091400
H	6.03500200	-2.97686800	-1.56169700
C	4.32572100	-1.76966700	-0.77168300
C	4.20644600	-0.66160600	-1.59950900
C	3.24632900	0.31091000	-1.34087200
C	2.98412200	1.54540800	-2.05576000
H	3.51965100	1.86543700	-2.93873500
C	1.98240400	2.18638300	-1.40538500
H	1.52914600	3.13714700	-1.63933400

C	1.61925000	1.34315000	-0.25829000
C	0.74929400	4.81618800	1.01043500
C	0.82679100	6.02878400	0.33366200
H	1.41271200	6.88457900	0.63606400
C	-0.00426500	5.89827200	-0.79163700
H	-0.20446700	6.64900100	-1.54466700
C	-0.55790700	4.61300600	-0.76292900
C	-1.48783400	3.97810500	-1.62464800
C	-1.93907200	2.71451100	-1.36480100
C	-2.85631700	1.86818600	-2.07256500
H	-3.37267400	2.15243900	-2.98054500
C	-2.95266900	0.68323900	-1.39738100
H	-3.55183600	-0.17992400	-1.64304300
C	-2.08688800	0.78133600	-0.23863900
C	-4.53685300	-1.91320900	1.02691900
C	-5.63387000	-2.37666300	0.32659200
H	-6.65569800	-2.37844400	0.67954300
C	-5.14044900	-2.84165100	-0.91469100
C	-3.75074600	-2.64580200	-0.92245700
C	-2.76303900	-2.95237700	-1.88932400
C	-1.40635000	-2.72641200	-1.62388200
C	-0.24558000	-2.99993200	-2.37219200
H	-0.22964500	-3.45047900	-3.35582300
C	0.86721700	-2.61096100	-1.59672300
H	1.90712500	-2.71265600	-1.86638200
C	0.36750500	-2.10329800	-0.39676700
B	2.26732000	-0.98100000	0.75272100
B	-0.45283400	2.50692200	0.76525700
B	-1.95934600	-1.62291700	0.66533600
H	-5.71393700	-3.28961200	-1.71557200
Cl	-4.53138100	-1.21340100	2.62296300
H	-3.06059500	-3.40268700	-2.82876000
H	4.87359200	-0.54768200	-2.44558300
H	-1.84313800	4.51158600	-2.49961200
H	-0.77762500	2.41351000	1.91260400
H	-1.65980800	-1.92940500	1.78875000
H	2.47923800	-0.60078400	1.87395000

SCF done: -3049.24410123 Hartree

No imaginary Frequency.

8.2 Theoretical calculations of 3a-3c (*meso-mesityl groups were omitted for simplicity*)

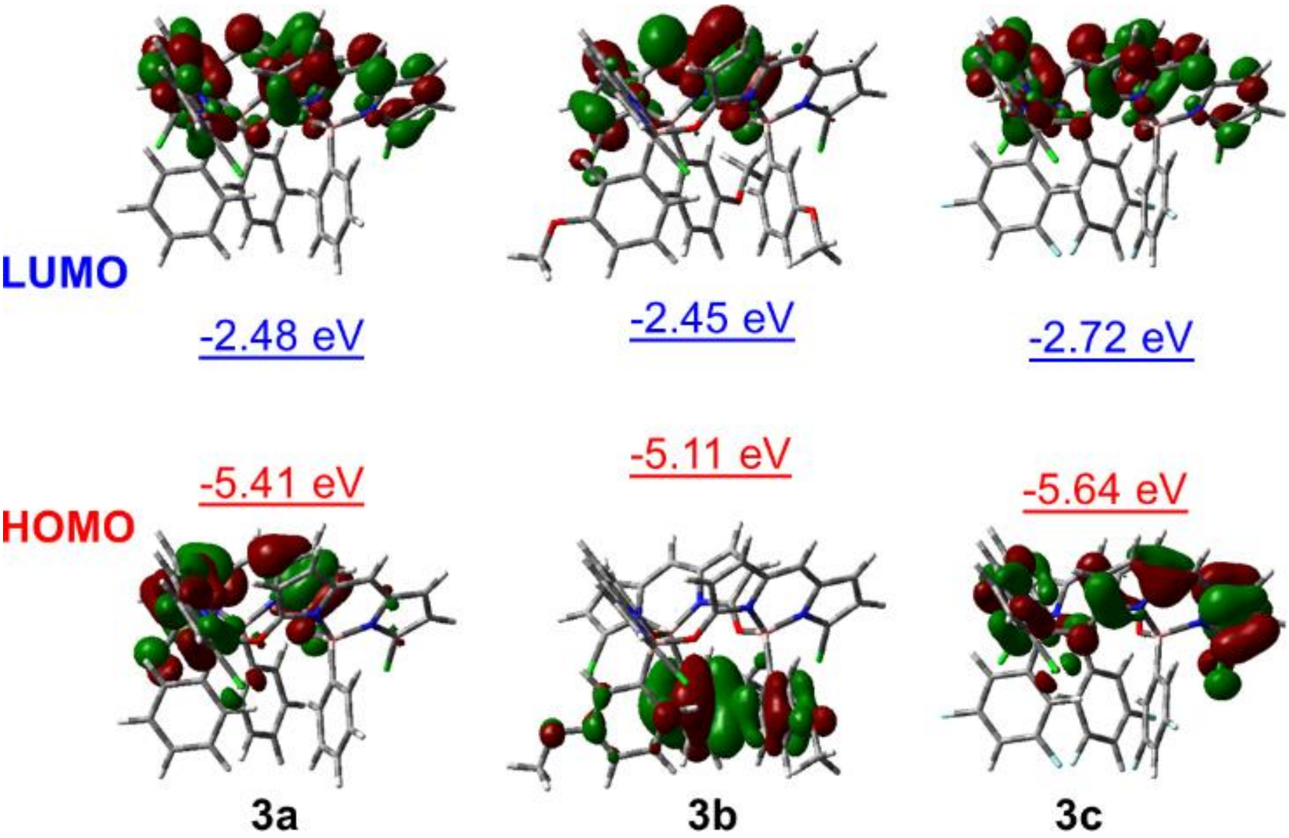

Figure S25. Pictorial presentation of LUMO, HOMO and their energy levels for **3a-3c**. Mesityl groups were omitted for clarity.

Table S9. Selected electronic excitation energies (eV) and oscillator strengths (*f*), configurations of the low-lying **singlet excited states** of oligomers **3a-3c** calculated by TDDFT//B3LYP/6-31G(d,p), based on the optimized ground state geometries.

	**Electronic transition**	**TD//B3LYP/6-31G(d, p)**			
		**Energy/ eV** [a]	***f*** [b]	**Composition** [c]	**CI** [d]
**3a**	S0→S1	2.4751 eV 500.92 nm	0.0006	HOMO → LUMO	0.4911
				HOMO → LUMO +1	0.2426
				HOMO → LUMO +1	0.2917
				HOMO -2 → LUMO +1	0.2078
	S0→S2	2.4766 eV 500.63 nm	0.0006	HOMO -1 → LUMO	0.4526
				HOMO -1 → LUMO +1	0.2934
				HOMO → LUMO +1	0.2555
				HOMO -2 → LUMO +1	0.2457
	S0→S3	2.4780 eV 500.33 nm	0.0002	HOMO -2 → LUMO	0.3827
				HOMO -1 → LUMO +2	0.3543
				HOMO -1 → LUMO +1	0.2376
				HOMO → LUMO +2	0.2354
**3b**	S0→S1	2.4621 eV 503.57 nm	0.0007	HOMO → LUMO	0.4645
				HOMO -1 → LUMO	0.3155
				HOMO → LUMO +1	0.2308
				HOMO -2 → LUMO	0.2358
	S0→S2	2.4703 eV 501.90 nm	0.0009	HOMO → LUMO +2	0.3682
				HOMO → LUMO	0.3459
				HOMO -1 → LUMO	0.3278
				HOMO -2 → LUMO	0.2110
	S0→S3	2.4819 eV 499.55 nm	0.0004	HOMO -1 → LUMO +1	0.3846
				HOMO -2 → LUMO +1	0.3104
				HOMO -1 → LUMO +1	0.2333
				HOMO -2 → LUMO	0.2841
**3c**	S0→S1	2.4727 eV 501.40 nm	0.0008	HOMO → LUMO	0.5026
				HOMO → LUMO +2	0.2752
				HOMO -2 → LUMO +2	0.2623
	S0→S2	2.4740 eV 501.14 nm	0.0009	HOMO -1 → LUMO	0.5083
				HOMO -2 → LUMO +1	0.2937
				HOMO -1 → LUMO +1	0.2289
				HOMO -1 → LUMO +2	0.1690
	S0→S3	2.4753 eV 500.88 nm	0.0001	HOMO -2 → LUMO	0.4652
				HOMO -1 → LUMO +1	0.3685
				HOMO → LUMO +2	0.3171

[a] Only the selected low-lying excited states are presented. [b] Oscillator strength. [c] Only the main configurations are presented. [d] The CI coefficients are in absolute values.

Optimized Geometries of the Compounds

3a, optimized S_0 state Geometry.

Cl	-4.67807300	-1.37169300	1.10965800
Cl	3.53435000	-3.36162700	1.11653100
O	-1.74224900	-0.39579800	-0.22479400
O	1.21258200	-1.31313500	-0.22167500
O	0.52818900	1.70196700	-0.22593800
N	-3.04898600	-2.52118100	-0.73208600
N	-0.58502200	-2.36194300	-1.21264700
N	3.70726200	-1.38177300	-0.73249700
N	2.33433100	0.66946400	-1.21697900
N	-0.65592500	3.89736000	-0.73532100
N	-1.75189100	1.68501100	-1.21559600
C	-1.39344800	-2.40073800	1.30436000
C	-1.46638000	-1.55568900	2.42197600
H	-1.69276900	-0.50432200	2.28121800
C	-1.24650000	-2.03899200	3.71452100
H	-1.31248300	-1.35738600	4.55939100
C	-0.94247400	-3.38546200	3.91976700
H	-0.76995000	-3.76318800	4.92489100
C	-0.86142500	-4.24513700	2.82194000
H	-0.62358700	-5.29640200	2.96827700
C	-1.08688800	-3.75347100	1.53574300
H	-1.02071300	-4.44156100	0.69403100
C	-4.30150400	-2.38984300	-0.22707000
C	-5.22963500	-3.14092000	-0.95735200
H	-6.28874800	-3.19515200	-0.75170000
C	-4.50579000	-3.77268000	-1.96873000
H	-4.88654100	-4.44037300	-2.73071700
C	-3.16201200	-3.38516600	-1.82628600
C	-2.03747500	-3.70035900	-2.60921500
C	-0.79606600	-3.19307200	-2.32474900
C	0.44882700	-3.35869400	-3.00191400
H	0.59037900	-3.95408900	-3.89497300
C	1.39676800	-2.64505600	-2.31778300
H	2.44591900	-2.55395800	-2.54673900
C	0.73213300	-2.03935600	-1.18732800
C	2.77800300	-0.00204300	1.30066000
C	3.78950800	0.94901100	1.52351600
H	4.34368500	1.35282500	0.67725600
C	4.10679900	1.39613300	2.80661300
H	4.89306000	2.13470000	2.94607200
C	3.41350400	0.89349700	3.91003900
H	3.65718700	1.23679000	4.91276900
C	2.40623100	-0.05216600	3.71330300
H	1.85729300	-0.45249200	4.56252400
C	2.09396300	-0.49080700	2.42384900
H	1.30303600	-1.22076600	2.29035300
C	4.22338000	-2.52910800	-0.22415600
C	5.33858500	-2.95670100	-0.95375600
H	5.91766500	-3.84455000	-0.74557500
C	5.52073000	-2.01657700	-1.96807400
H	6.28909000	-2.01260700	-2.73032400
C	4.51088600	-1.04870800	-1.82795500
C	4.21907200	0.08016900	-2.61377200
C	3.15797300	0.90050800	-2.33056700

C	2.67688200	2.05921200	-3.01000300
H	3.12055400	2.47793500	-3.90437100
C	1.58502200	2.52363700	-2.32585900
H	0.98041000	3.38555700	-2.55575400
C	1.39510100	1.64753400	-1.19323500
C	-1.38642300	2.40725700	1.30065800
C	-2.71341600	2.81558300	1.52409700
H	-3.33919400	3.09629600	0.67797800
C	-3.25837300	2.87057600	2.80738700
H	-4.28930300	3.18799200	2.94721500
C	-2.47791200	2.51760400	3.91057500
H	-2.89626400	2.56026600	4.91350200
C	-1.15787200	2.11007700	3.71344300
H	-0.53810400	1.83143400	4.56257500
C	-0.62277200	2.05469100	2.42375800
H	0.40239200	1.72683800	2.29055600
C	0.08436800	4.91590400	-0.22981900
C	-0.10148900	6.09560200	-0.95965800
H	0.38153100	7.03967000	-0.75377100
C	-1.01073400	5.78533200	-1.97106700
C	-1.34774900	4.42793900	-1.82911200
C	-2.18324900	3.61212100	-2.61235800
C	-2.36480700	2.28326900	-2.32840200
C	-3.13029900	1.28792100	-3.00600500
H	-3.71613500	1.46311300	-3.89941900
C	-2.98696500	0.11013000	-2.32165900
H	-3.43303600	-0.84392700	-2.55036900
C	-2.13055100	0.38284300	-1.19089600
B	-1.69075800	-1.89754000	-0.19310800
B	2.48752400	-0.51709600	-0.19412700
B	-0.79739900	2.40998800	-0.19529000
H	-1.39811000	6.44933600	-2.73292800
Cl	1.15478500	4.73111100	1.10639400
H	-2.16409600	-4.35114500	-3.46874200
H	4.84538500	0.29469000	-3.47403600
H	-2.68317600	4.04759300	-3.47188600

SCF done: -3742.47315436 Hartree
No imaginary Frequency.

3b, optimized S_0 state Geometry.

Cl	-4.87556200	0.60990000	0.48003000
Cl	2.90433400	3.86600500	0.99172100
Cl	1.84310000	-4.49856800	0.88127000
O	0.61947100	-1.68178800	-0.51533800
O	1.19162300	1.35567900	-0.46040400
O	-1.71702100	0.34035200	-0.65697000
N	-0.83568200	2.26094900	-1.57597100
N	-1.37878200	-1.91632200	-1.64072200
N	-3.72674000	-1.07877600	-1.30657500
N	2.49506400	-0.30473800	-1.38469300
N	1.01954700	3.84507800	-0.96343600
N	2.90780200	-2.73602500	-0.88754500
C	-1.09386800	3.06868000	-2.69509000
C	-0.04684200	-2.14675100	-1.53101900
C	-1.70331900	1.21910000	-1.61458700
C	-2.30140100	-1.64921800	0.82369500
C	-1.83059200	-2.58247400	-2.79115000

C	2.02051600	0.96582600	-1.38274900
O	5.41658400	0.06674300	3.09998600
C	0.66141800	4.62134000	-2.07050300
O	-2.97425700	-4.90982600	2.32957900
C	-4.10360500	-1.81530000	-2.43410700
C	-2.52825300	1.32469700	-2.79599400
H	-3.30259800	0.63119600	-3.08028700
C	2.47732700	-1.11420700	1.13326400
C	3.84634200	-2.74246200	-1.92476000
C	-0.38234600	4.21781200	-2.92245200
C	3.40828800	-0.41441200	-2.44556300
C	-0.37534900	2.75409300	0.98003100
C	-2.15044200	2.47017600	-3.44441500
H	-2.56387600	2.87600300	-4.35896200
C	2.60806700	1.69925700	-2.48012600
H	2.40691600	2.73058900	-2.71933800
C	0.39361000	-2.95613900	-2.64313100
H	1.40457600	-3.28989700	-2.81029700
C	-0.71362700	-3.22501000	-3.40337400
H	-0.76328500	-3.81885100	-4.30723000
C	4.07440000	-1.58696300	-2.69280600
C	-3.14978000	-2.54734400	-3.16294000
C	-4.85232000	-0.46154900	-0.86756300
C	3.46620400	0.84302700	-3.11761800
H	4.09066600	1.05484700	-3.97632400
C	-2.59720600	-3.00419700	1.01190700
H	-2.77428800	-3.66401600	0.16536100
C	3.77848800	-0.65747400	1.42296500
H	4.48323300	-0.51706200	0.60895600
C	2.89244100	-3.99756700	-0.38810400
C	-5.47792200	-1.63020400	-2.66498000
H	-6.04301800	-2.08245100	-3.46970200
C	4.39730200	-4.03093700	-2.03795600
H	5.15075900	-4.32774900	-2.75607700
C	2.07458600	4.47642900	-0.38916600
C	-2.08516700	-0.85908300	1.96627300
H	-1.84397400	0.19185800	1.85751800
C	0.13915700	2.13962400	2.13533900
H	0.97262000	1.45180200	2.05396400
C	-2.67906000	-3.56958600	2.29059500
C	2.41809300	5.63944700	-1.08728000
H	3.22504300	6.30872900	-0.82676300
C	-5.95343200	-0.77070500	-1.67438100
H	-6.95694700	-0.39765400	-1.53048700
C	1.52016600	5.73107800	-2.15110000
H	1.47456300	6.50319200	-2.90819300
C	4.17184700	-0.38314200	2.73529900
C	3.79231100	-4.82803000	-1.06639100
H	3.95645000	-5.87453700	-0.85471700
B	2.09922200	-1.46069400	-0.39123400
B	0.25984100	2.53568200	-0.48142800
C	1.58440700	-1.28746500	2.19590400
H	0.57411900	-1.62767900	2.00126600
B	-2.27196400	-1.05624500	-0.67162400
C	-2.46597200	-2.77172000	3.41938100
H	-2.52591500	-3.18436800	4.42053700
C	-0.41840300	2.39941100	3.38489700
H	-0.00641500	1.91637100	4.26786700

C	-1.46089200	3.62465400	1.13077000
H	-1.90282700	4.12677100	0.27303300
C	-2.17008200	-1.41657800	3.23988000
H	-2.00109100	-0.79011500	4.11270600
C	3.26511200	-0.56642000	3.78867600
H	3.59116900	-0.34927500	4.80161300
O	-3.08854300	4.75616200	2.38745400
C	1.98024200	-1.01736200	3.51096800
H	1.27282500	-1.15895200	4.32473700
C	-1.50353500	3.27043400	3.52673200
H	-1.92235100	3.45714600	4.50950600
C	-2.02727100	3.88527600	2.38493500
C	6.37035200	0.28935200	2.07914500
H	6.60640600	-0.63431800	1.53237100
H	7.27154100	0.64823800	2.58153700
H	6.02976800	1.04904900	1.36197300
C	-3.05826300	-5.53409100	3.59665500
H	-3.29042100	-6.58353900	3.40186700
H	-3.85539000	-5.09770600	4.21467400
H	-2.10857800	-5.47075300	4.14580300
C	-3.70041200	5.05355700	3.62815000
H	-4.11835600	4.15481300	4.10267400
H	-4.51110800	5.75108800	3.40583800
H	-2.99746300	5.52919200	4.32628700
H	-0.61576900	4.82859300	-3.78888700
H	-3.46909400	-3.08516100	-4.05006900
H	4.78560100	-1.63486000	-3.51146700

SCF done: -4086.03986328 Hartree
No imaginary Frequency.

3c, optimized S_0 state Geometry.

Cl	-1.61386800	4.59308800	0.71706200
Cl	4.78911600	-0.89719500	0.71232200
Cl	-3.16492300	-3.70330500	0.73581500
O	-1.24694700	-1.28074200	-0.62552700
O	1.73068400	-0.43885600	-0.63190900
O	-0.48927600	1.70977400	-0.63095800
N	1.59050200	1.84066100	-1.61477900
N	-2.39970300	0.45460200	-1.60793800
N	-2.68595500	2.90352400	-1.11857200
N	0.79522300	-2.30198900	-1.61260400
N	3.85734200	0.87058600	-1.12581000
N	-1.17733600	-3.77883100	-1.11327900
C	2.15658400	2.48963000	-2.72441300
C	-2.00485900	-0.84301300	-1.58840100
C	0.26817300	2.14396800	-1.59576700
C	-2.44708800	1.24816800	0.91175900
C	-3.24994300	0.62098700	-2.71333600
C	1.72199700	-1.31120000	-1.59721500
C	4.35242000	1.59512600	-2.21633700
C	-3.56470000	2.97128000	-2.20606500
C	-0.04949300	2.98360100	-2.72480400
H	-1.02690300	3.37439600	-2.95643100
C	0.14554200	-2.74026800	0.91077800
C	-0.80355800	-4.56999000	-2.20582300
C	3.49548800	2.38446200	-3.00207700
C	1.06835300	-3.11778000	-2.72291400

C	2.30592000	1.49360800	0.90567600
C	1.12150000	3.19607700	-3.40467800
H	1.26563300	3.79252100	-4.29653100
C	2.60378600	-1.45842300	-2.72892000
H	3.43240800	-0.80986400	-2.96230800
C	-2.58001400	-1.53832900	-2.71359000
H	-2.43363400	-2.58105600	-2.94378900
C	-3.35083900	-0.62968700	-3.39106700
H	-3.94458300	-0.80309900	-4.27951400
C	0.30510900	-4.22371300	-2.99692700
C	-3.82583400	1.83452100	-2.98998900
C	-2.61932000	4.16132000	-0.61162500
C	2.19729100	-2.57812800	-3.40688100
H	2.63833700	-3.00334000	-4.29940900
C	-3.76742200	0.83311100	1.15762900
H	-4.48522500	0.70886800	0.35182100
C	1.18010100	-3.66063400	1.15123000
H	1.65277100	-4.21144200	0.34295500
C	-2.29525600	-4.35347000	-0.59973700
C	-4.02636700	4.29216700	-2.34080200
H	-4.72051300	4.63732300	-3.09597600
C	-1.71603100	-5.63120100	-2.33722000
H	-1.67250800	-6.40234500	-3.09531300
C	4.91502500	0.18687500	-0.61881500
C	-1.57972200	1.38460100	2.00380300
H	-0.55152000	1.69260600	1.86415200
C	1.99654200	0.67524300	2.00062400
H	1.75154200	-0.37012700	1.86438800
C	-4.17828600	0.56516400	2.45673600
C	6.08481000	0.44295700	-1.34296600
H	7.05397900	0.01390200	-1.13445800
C	-3.42700100	5.04743500	-1.33326000
H	-3.53793100	6.10140300	-1.12426600
C	5.72743300	1.33630200	-2.35246200
H	6.37151600	1.76326100	-3.11018100
C	1.62666600	-3.87672100	2.44843000
C	-2.66243500	-5.49437700	-1.32215700
H	-3.51744900	-6.11918300	-1.10900100
B	-0.38407000	-2.50911900	-0.59410000
B	2.36336800	0.92188300	-0.60067100
C	-0.41238900	-2.06711100	2.00626300
H	-1.20514000	-1.34318800	1.86994300
B	-1.98421600	1.58242300	-0.59551800
C	-3.33123400	0.69044400	3.55202900
H	-3.66587800	0.47016900	4.55870700
C	1.98182700	1.20848100	3.28453700
C	2.60283500	2.84600100	1.14817100
H	2.84954500	3.52920300	0.34047700
C	-2.03107600	1.10457400	3.28885200
C	1.08928100	-3.21488700	3.54657100
H	1.45439900	-3.38854800	4.55172400
C	0.06495600	-2.31148400	3.28874200
C	2.26894100	2.54318100	3.54422000
H	2.24689700	2.94482000	4.55021400
C	2.57792900	3.33833500	2.44643100
H	3.90669700	2.91024200	-3.85795200
H	-4.49022800	1.92851800	-3.84309000
H	0.55031300	-4.84346800	-3.85359100

F	2.86335000	4.64396600	2.65879600
F	1.67924700	0.40227500	4.32266700
F	-0.48412000	-1.64915000	4.32772800
F	2.62684200	-4.76359600	2.65867900
F	-1.17784400	1.24044800	4.32459500
F	-5.45230700	0.16316800	2.67232900

SCF done: -4337.87995786 Hartree

No imaginary Frequency.

9. Biological Studies

Cell Culture

The HeLa cells were cultured in a Roswell Park Memorial Institute 1640 medium (RPMI-1640, Gibco, America) with 10% fetal bovine serum (FBS, Lonsera, Shanghai, China) at 37 °C with 5% CO_2.

Preparation of 3b and 3d micelles

To a tube containing 2 mL THF was added 50 μL photosensitizer **3b** or **3d** solution (1 mM in THF) and 100 μL Cremophor EL solution (7.5 mg/mL in THF). The mixture was sonicated for 30 min. THF was removed under reduced pressure, the remaining oil was dissolved in 1640 complete medium (5 mL) filtered through a 0.22 μM membrane filter. The obtained **3b** or **3d** micelles (10 μM, 5 mL) were then stored at 4°C for further experiments.

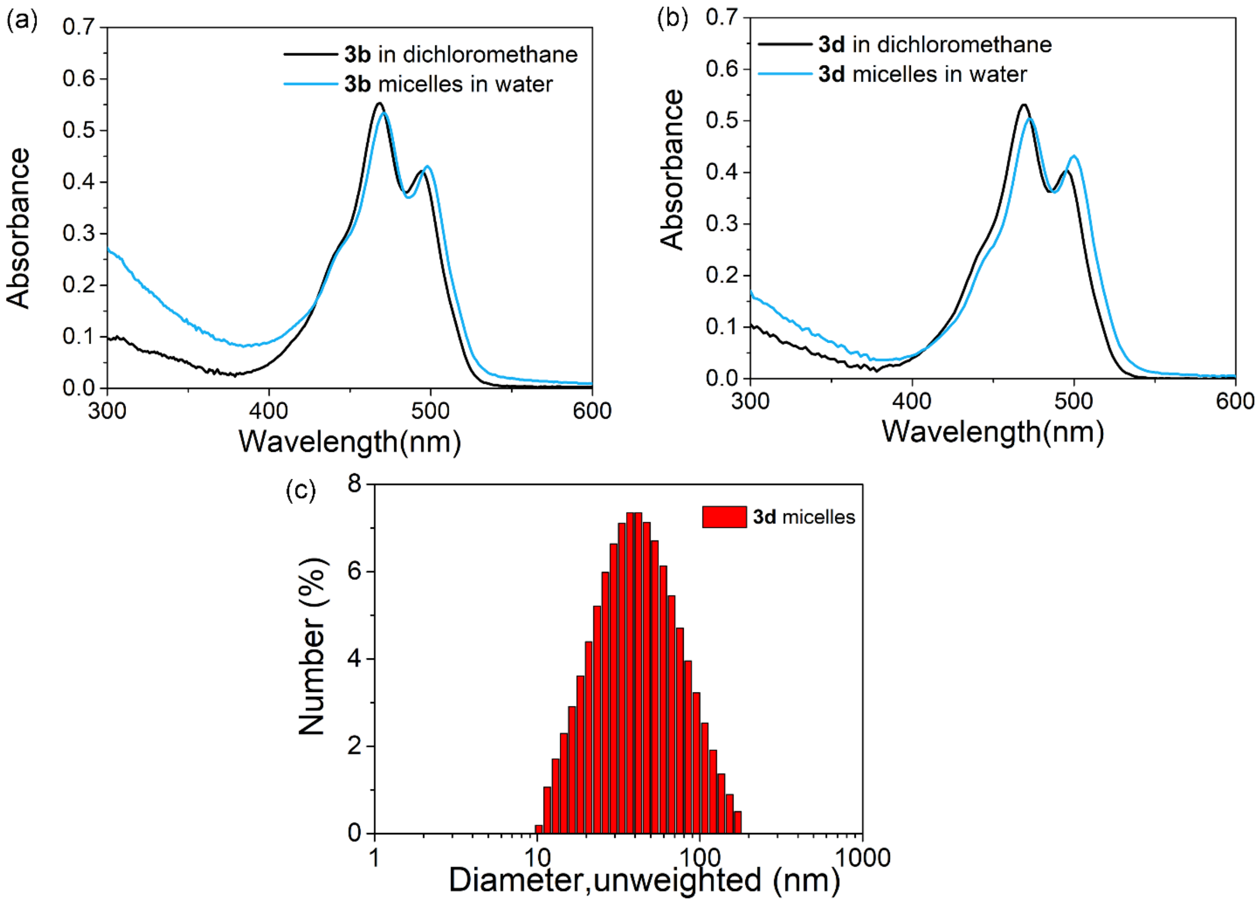

Figure S26. UV-vis spectra of **3b** (a) and **3d** (b) micelles (10 μM) in different solvents, and the hydrodynamic diameter of **3d** micelles in water via DLS (c).

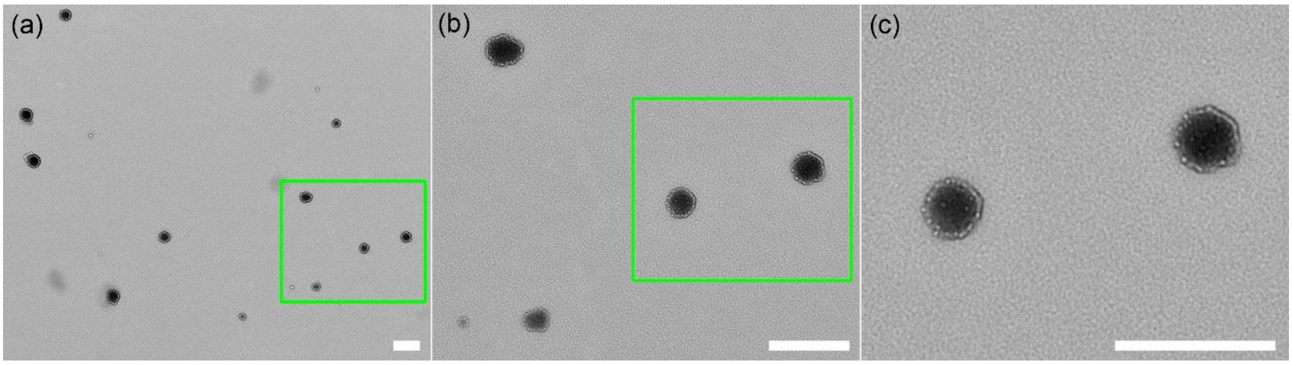

Figure S27. (a) TEM images of **3d** micelles (5 μM). (b) Enlarged images of a. (c) Enlarged images of b. Scale bars = 200 nm.

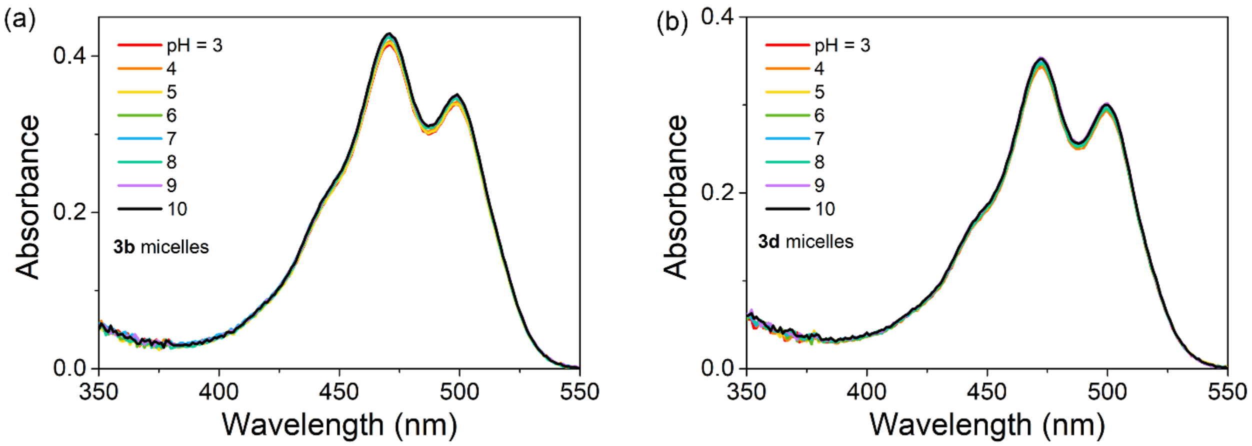

Figure S28. Absorption spectra of **3b** (a) and **3d** (b) micelles (5 μM) recorded at different pH values (3.0, 4.0, 5.0, 6.0, 7.0, 8.0, 9.0, 10.0).

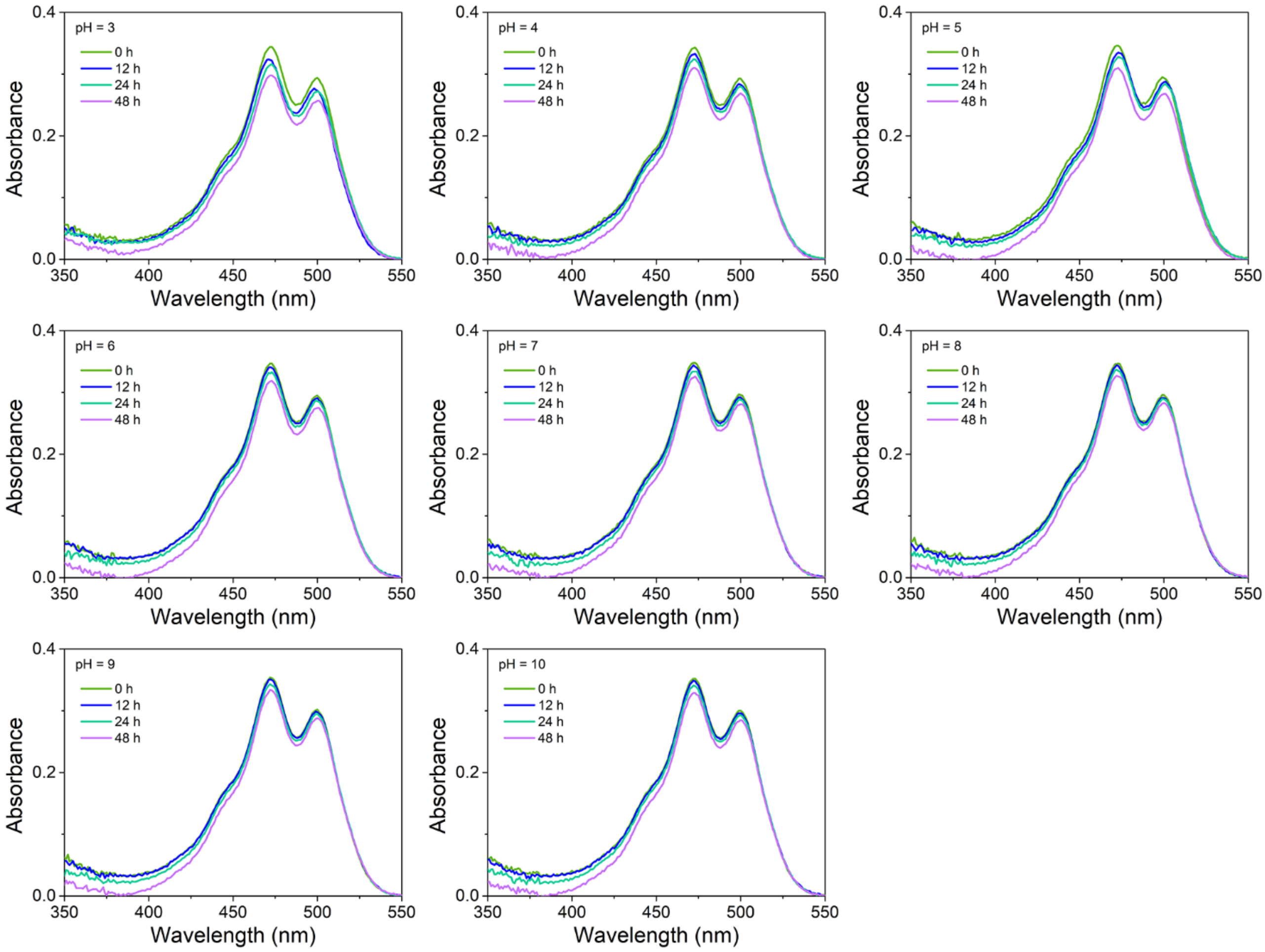

Figure S29. Absorption spectra of **3b** micelles recorded at different pH values (3.0, 4.0, 5.0, 6.0, 7.0, 8.0, 9.0, 10.0) and different time intervals (0, 12, 24, 48 h).

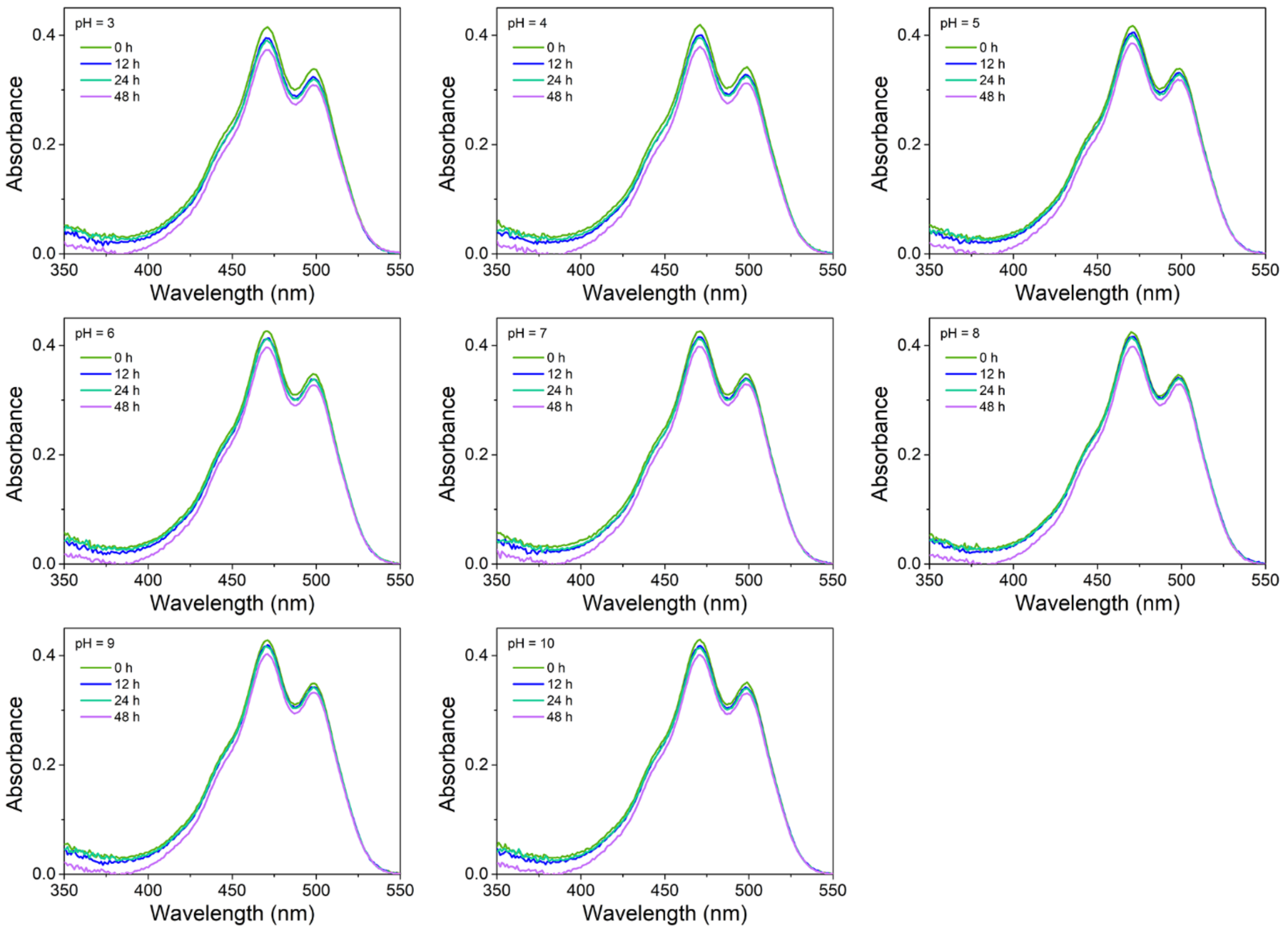

Figure S30. Absorption spectra of **3d** micelles recorded at different pH values (3.0, 4.0, 5.0, 6.0, 7.0, 8.0, 9.0, 10.0) and different time intervals (0, 12, 24, 48 h).

Darkcytotoxicity of 3d determined by the CCK-8 method.

The HeLa cells were plated at 5000 cells per well in a 96-well plate in a 1640 medium and were allowed to grow for 24 h. A gradient concentration of **3d** micelles from 0 to 100 μM in a fresh medium was added as a replacement, and the cells were incubated for 24 h. The working solutions were then removed, and the cells were washed with PBS buffer for two times. A total of 10 μL of CCK-8 (Cell Counting Kit-8, BIOMIKY) was added into each well, and the cells were further incubated at 37 °C for 1 h in a 5% CO_2 humidified atmosphere. The plate was shaken for 5 min, and the absorbance was measured at 450 nm using a microplate reader (Multiskan Sky).

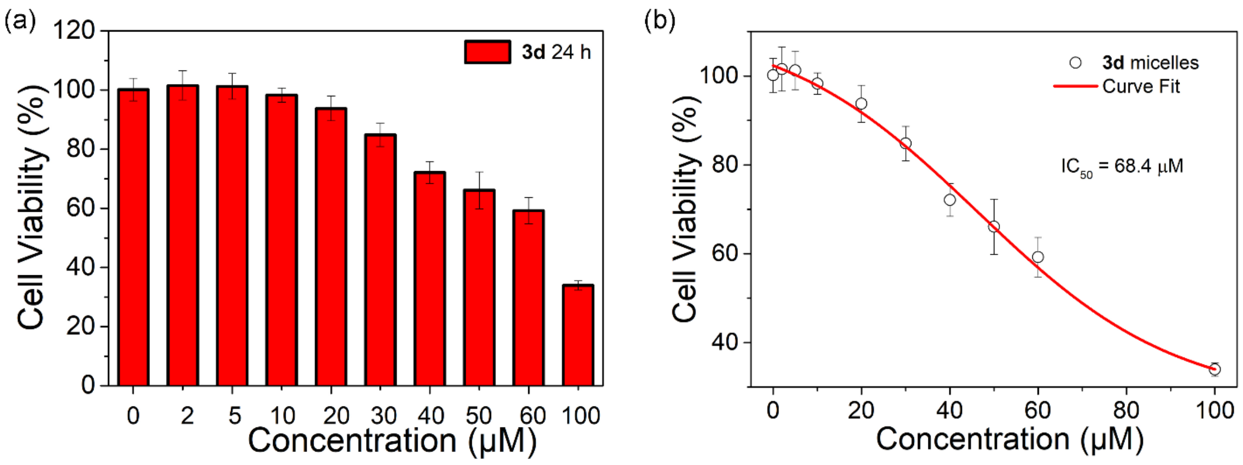

Figure S31. (a) Viability of HeLa cells treated with 0, 2, 5, 10, 20, 30, 40, 50, 60 and 100 μM **3d** micelles for 24 h. (b) Curve fitting between the cell viability and the concentration of **3d** micelles. IC_{50} = 68.4 μM.

Photocytotoxicity determined by the CCK-8 method.

The HeLa cells were plated at 5000 cells per well in a 96-well plate in a 1640 medium and were allowed to grow for 24 h. A gradient concentration of **3b** or **3d** micelles from 0 to 1 μM in a fresh medium was added as a replacement, and the cells were incubated for 12 h. The experimental group of the cells were illuminated with a LED light source (2100 lux, distance between light source and cells: 14 cm) for 20 min in every 40 min for a repeated 9 times (final total illumination time was exactly 3 h) at room temperature. This 6 h period of light - dark cycles was followed by a 6 h incubation solely in the dark (total 24 h) in the incubator. The control groups of the cells were incubated in the dark, for the duration of 24 h under identical environmental conditions except illumination. After incubation 24 h, the working solutions were then removed, and the cells were washed with PBS buffer for two times. A total of 10 μL of CCK-8 (Cell Counting Kit-8, BIOMIKY) was added into each well, and the cells were further incubated at 37 °C for 50 min in a 5% CO_2 humidified atmosphere. The plate was shaken for 5 min, and the absorbance was measured at 450 nm using a microplate reader (Multiskan Sky). The photocytotoxicity of a reference photosensitizer Rose Bengal (**RB)** was studied using the above method for comparation (Figure S29).

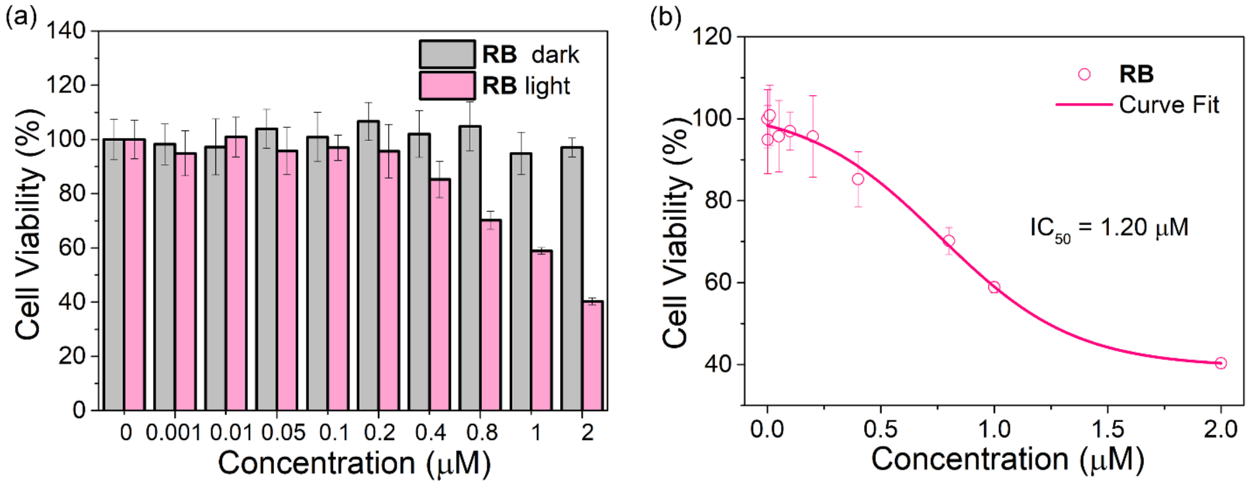

Figure S32. (a) Cell suspensions were seeded in 96-well flat-bottom plates and varying concentrations of the sensitizers were added to each well. Cells were kept either in the dark, or under illumination with a green LED lamp for a period of 3 h at 37 °C. (b) Curve fitting between the cell viability and the concentration of **RB**. IC_{50} = 1.2 μM.

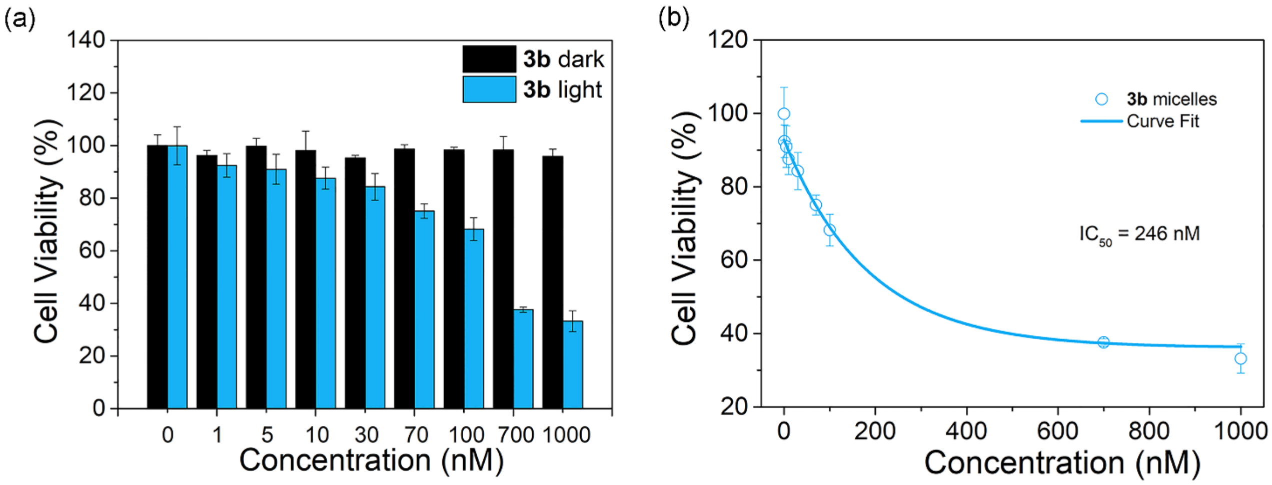

Figure S33. (a) Cell suspensions were seeded in 96-well flat-bottom plates and varying concentrations of the sensitizers were added to each well. Cells were kept either in the dark, or under illumination with a cyan LED lamp at 2100 lux flow rate for a period of 3 h at 37 °C. (b) Curve fitting between the cell viability and the concentration of **3b** micelles. IC_{50} = 246 nM.

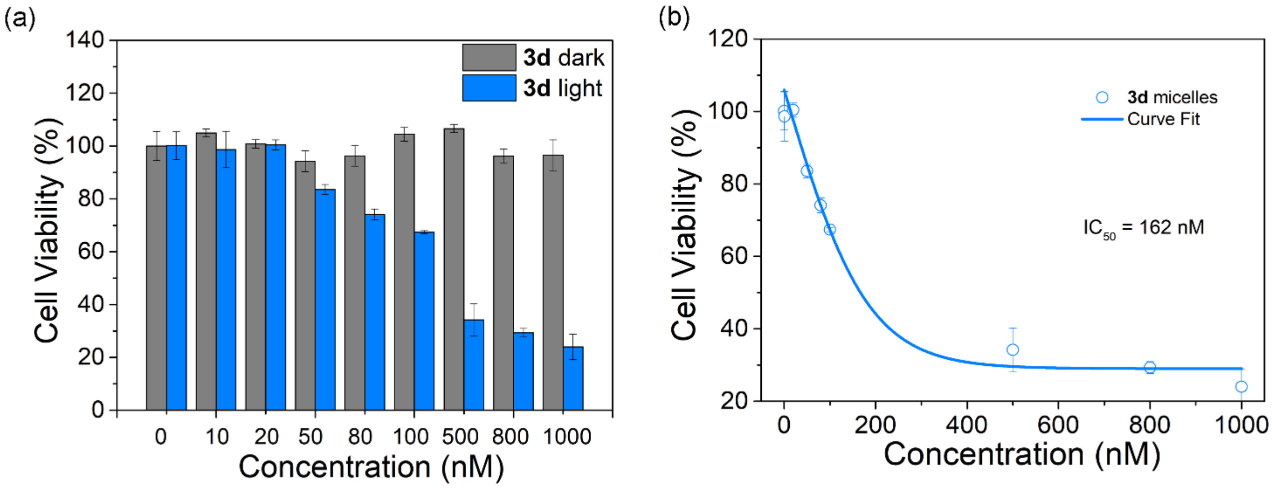

Figure S34. (a) Cell suspensions were seeded in 96-well flat-bottom plates and varying concentrations of the sensitizers were added to each well. Cells were kept either in the dark, or under illumination with a cyan LED lamp at 2100 lux flow rate for a period of 3 h at 37 °C. (b) Curve fitting between the cell viability and the concentration of **3d** micelles. IC_{50} = 162 nM.

Cell Incubation and Imaging

A total of 30000 HeLa cells were seeded into a glass bottom dish with the same procedure above. A solution of **3b** or **3d** micelles solution in RPMI-1640 medium (10 µM) was added to the above cells and incubated for another 1 h at 37 °C with 5% CO_2. The working solutions were then removed, and the cells were then washed with PBS two times and fixed by 4% formaldehyde for 20 min. The organelle tracer DAPI (0.08 µg/mL) was added subsequently and incubated for 20 min to stain the nucleus. The above solution in dish was removed, and the cells were washed with PBS buffer two times before imaging using a confocal fluorescence microscope (Leica Microsystems SP8 MP).

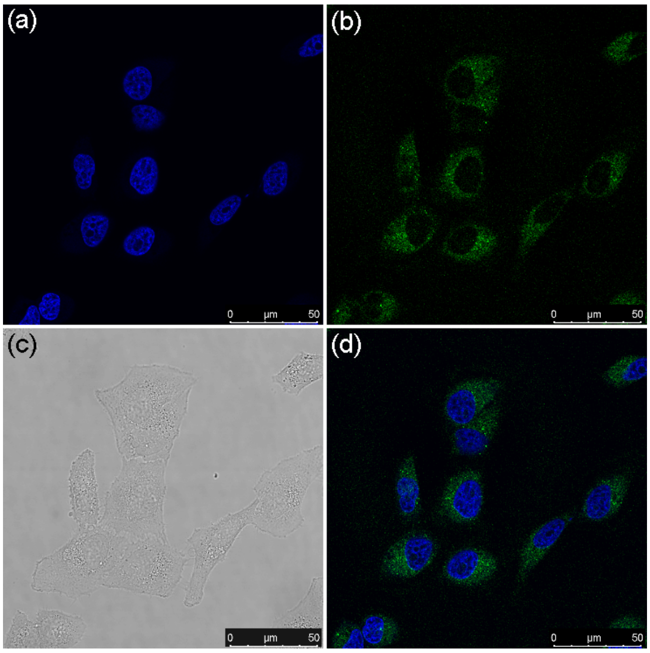

Figure S35. Fluorescence images using **3b** micelles in HeLa cells. The cells were incubated with **3b** micelles (10.0 µM) for 1 h at 37 °C. Images for **3b** were then recorded using excitation wavelengths of 488 nm, and recording over the 500-700 nm spectral regions. (a) DAPI fluorescence, (b) **3b** fluorescence (c) bright field and (d) merged images of parts b and c. Scale bar: 50 µM.

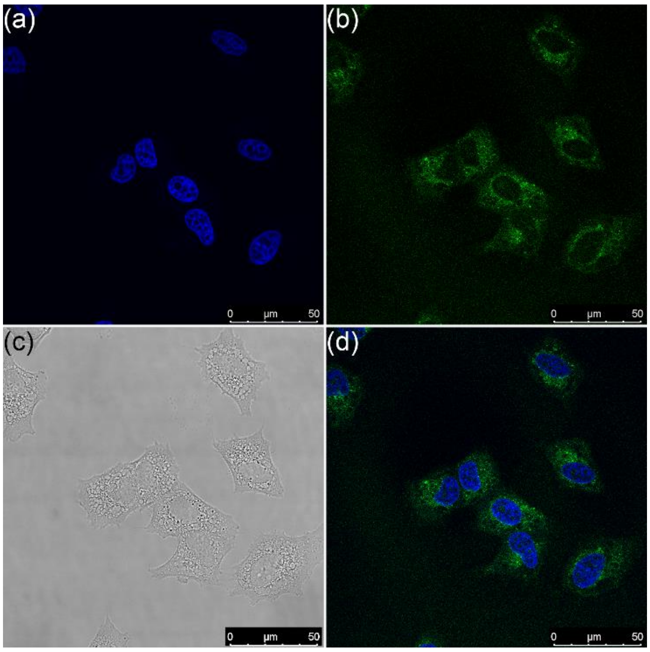

Figure S36. Fluorescence images using **3d** micelles in HeLa cells. The cells were incubated with **3d** micelles (10.0 μM) for 1 h at 37 °C. Images for **3d** were then recorded using excitation wavelengths of 488 nm, and recording over the 500-700 nm spectral regions. (a) DAPI fluorescence, (b) **3d** fluorescence (c) bright field and (d) merged images of parts b and c. Scale bar: 50 μM.

Live–dead cell staining

Live–dead cell staining analysis was also performed to evaluate cell viability. [22] Briefly, a total of 30000 HeLa cells were seeded into a glass bottom dish and were cultured in culture media (RPMI-1640, supplemented with 10% FBS) at 37 °C in an atmosphere of 5% CO_2 and 95% humidified atmosphere for 12 h. Cells in control-1 wells were incubated in the incubator for 24 h. Cells in control-2 wells were incubated in the incubator for 12 h without **3b** or **3d** micelles (1 μM), then illuminated under a LED lamp (220 lux, distance between light source and cells: 11 cm) for 20 min and replaced in incubator for another 20 min to avoid heat effect for a total of 9 times repetitions (total 6 h, final total illumination time was exactly 3 h) at room temperature. Then cells were further incubated for 6 h in incubator (total 24 h). Cells in control-3 wells were treated with **3b** or **3d** micelles (at a final concentration of 1 μM) and were kept in the dark in the same condition for 24 hours. Cells in control-4 were incubated **3b** or **3d** micelles for 12 h then illuminated for 3h that process is same as control-2 wells. Then cells were further incubated for 6 h in incubator (total 24 h). All the control groups cells were then washed with AO staining buffer for once and replaced with AO-PI mixture in the dark at room temperature. After 15 min, these plates were taken pictures immediately. AO was excited at 488 nm, and collected from 500-540 nm; PI was excited at 552 nm, and collected from 650-800 nm.

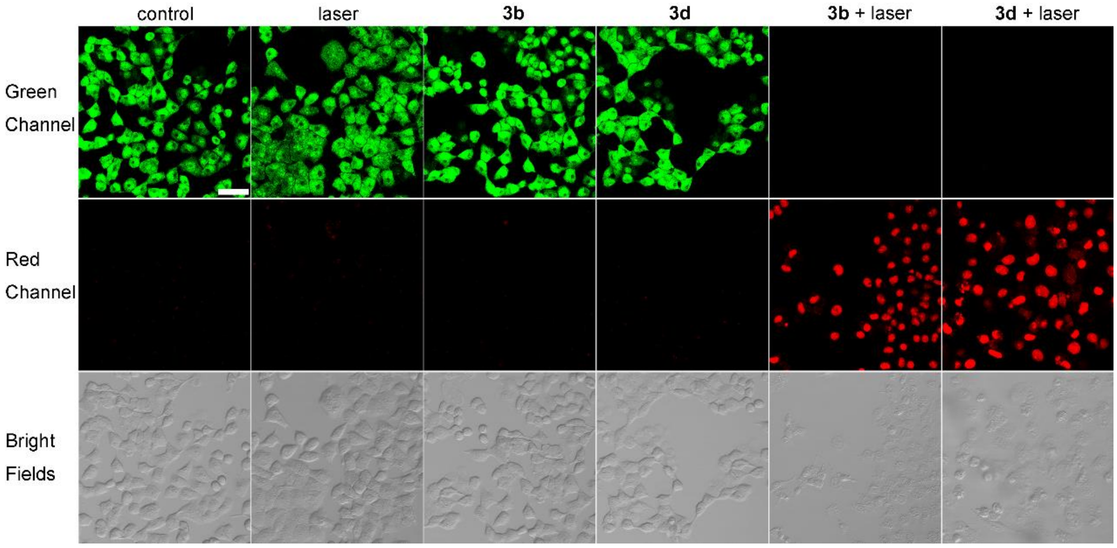

Figure S37. Fluorescence images of AO (green) and PI (red) costaining HeLa cells incubated with 1 μM of **3b** or **3d** micelles without and with LED irradiation (480 nm, 2100 lux, 3 h). Scale bar = 50 μm.

10. Scanned NMR and HRMS spectra

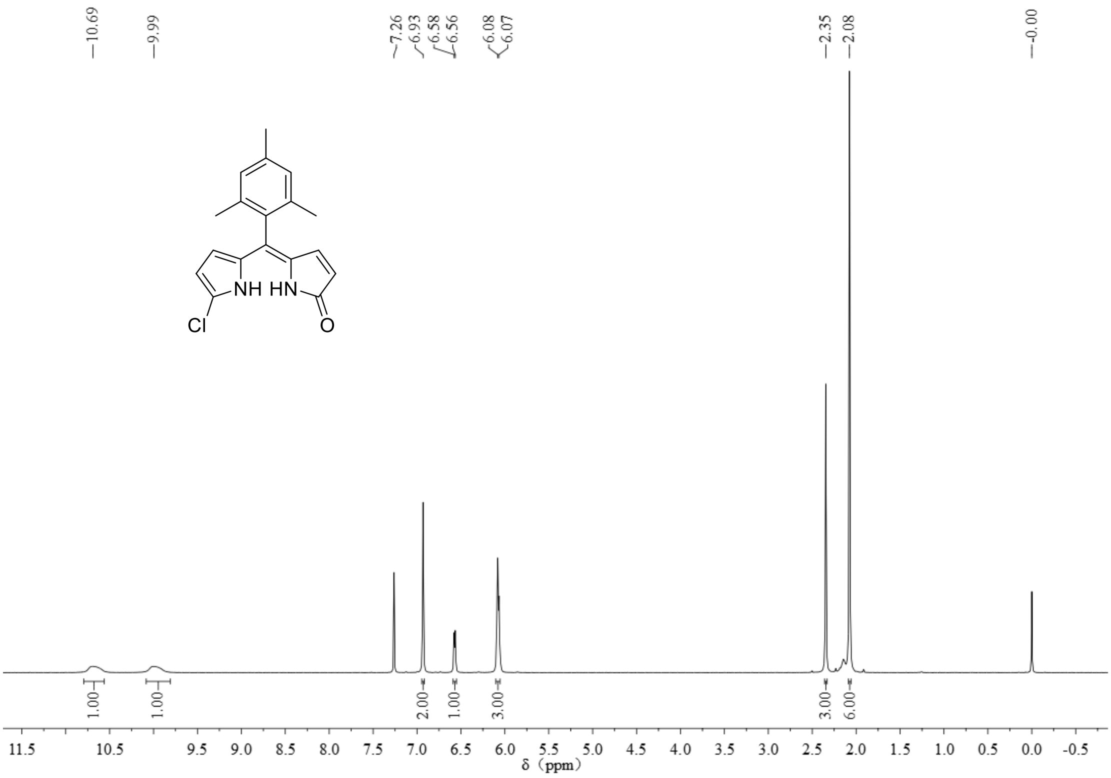

^{1}H NMR spectra of compound **2**

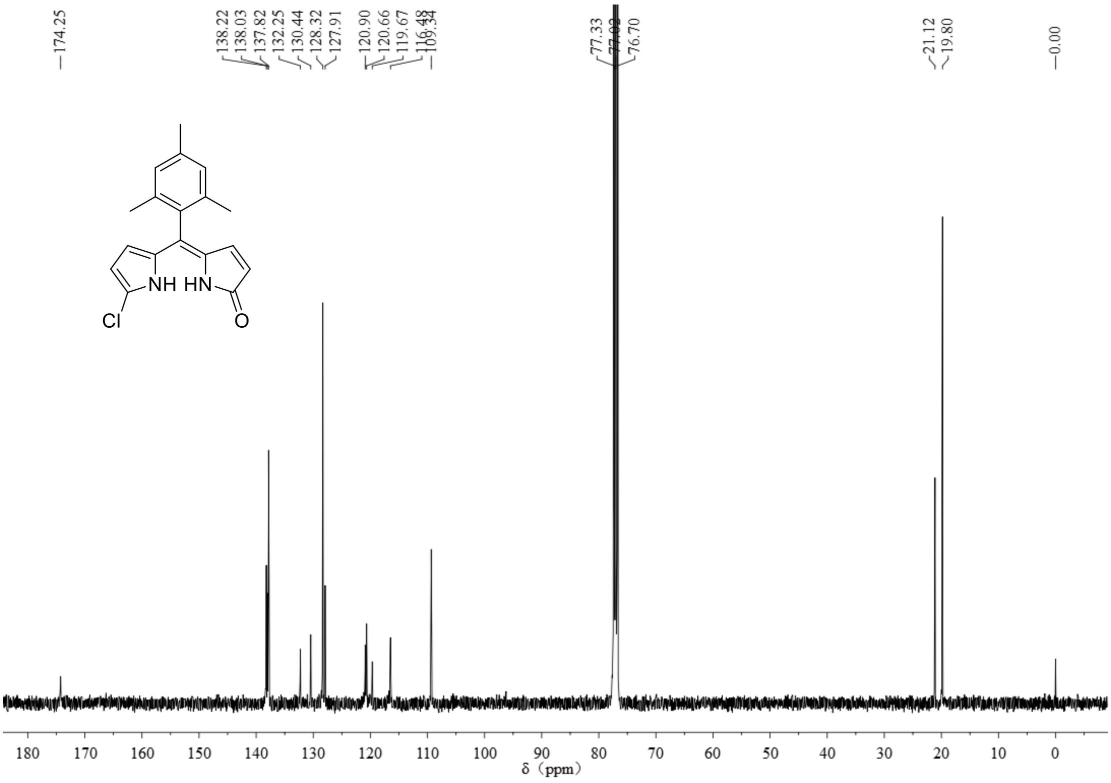

^{13}C NMR spectra of compound **2**

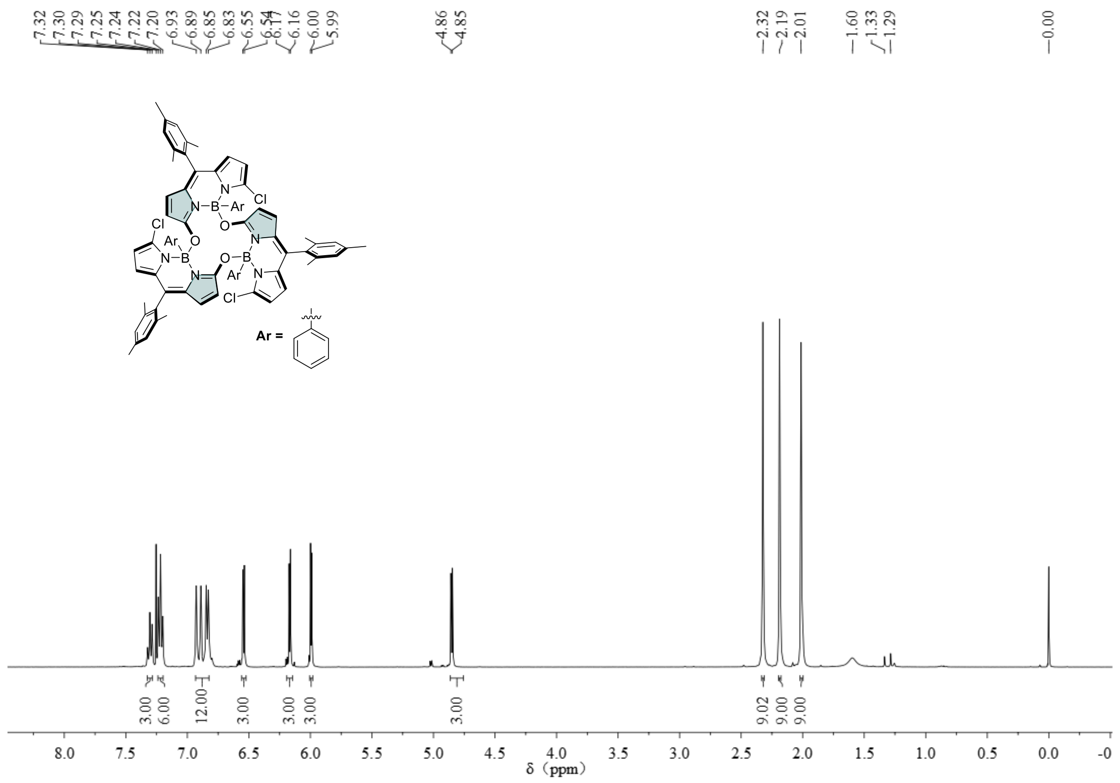

^{1}H NMR spectra of compound **3a**

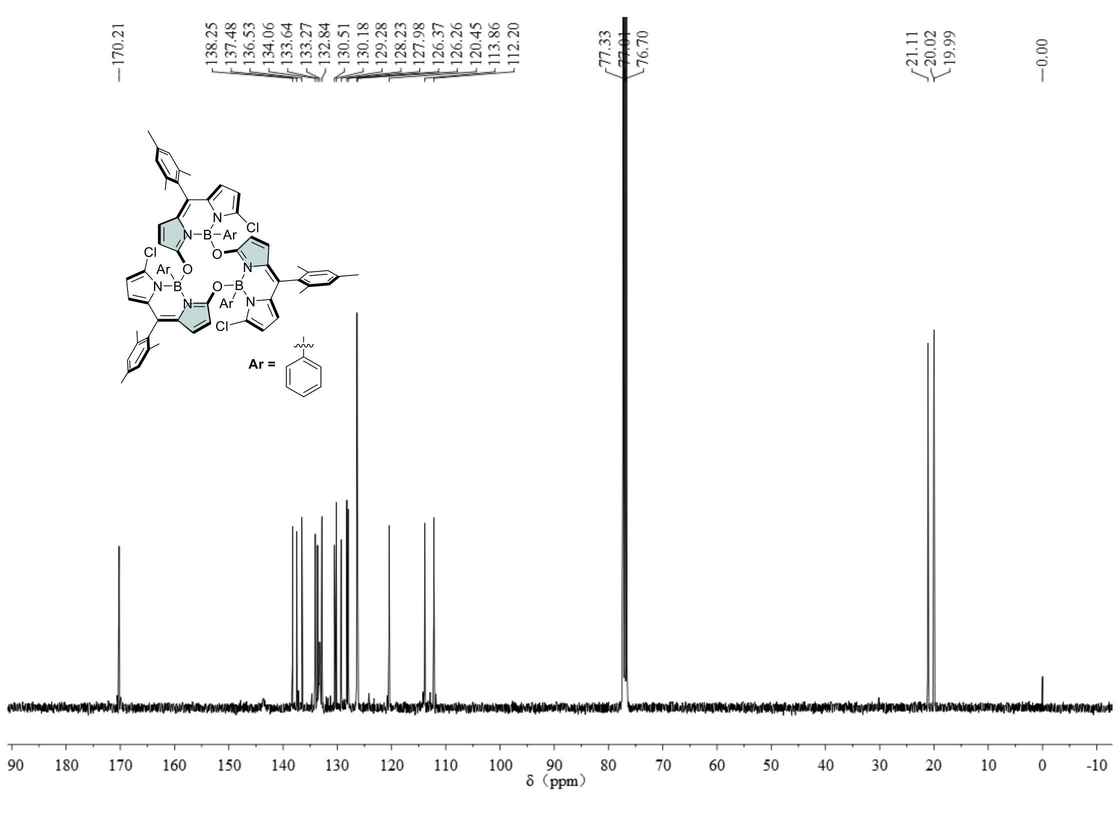

^{13}C NMR spectra of compound **3a**

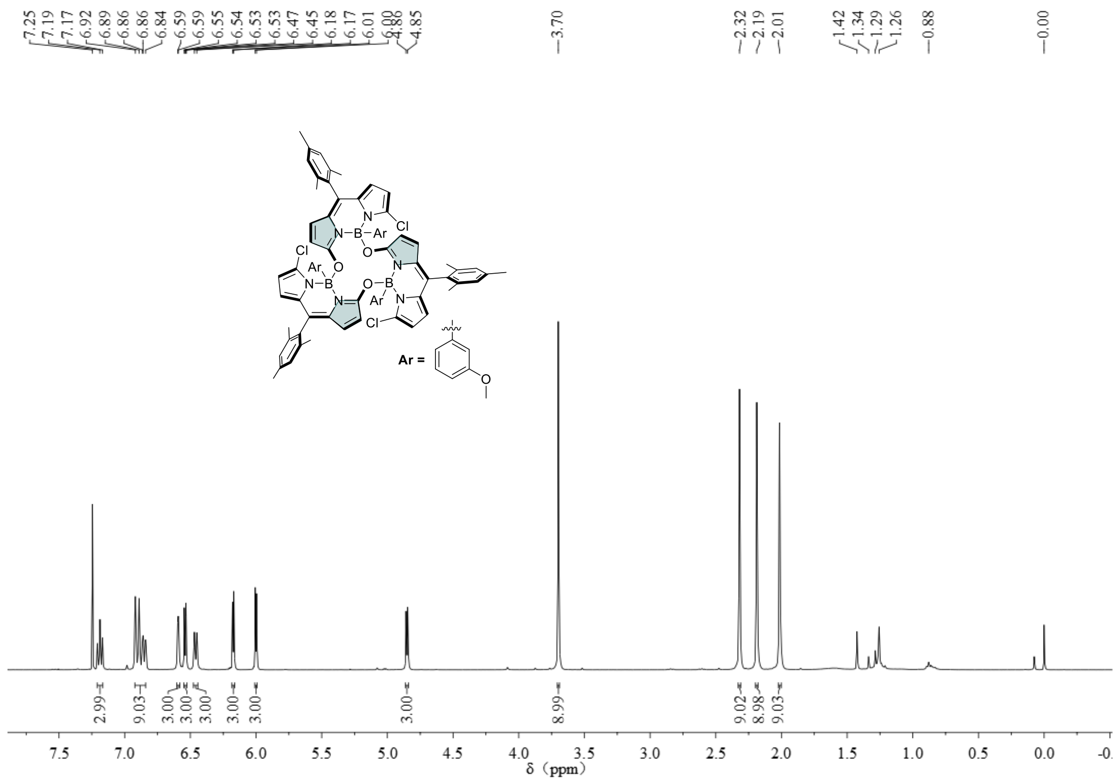

^{1}H NMR spectra of compound **3b**

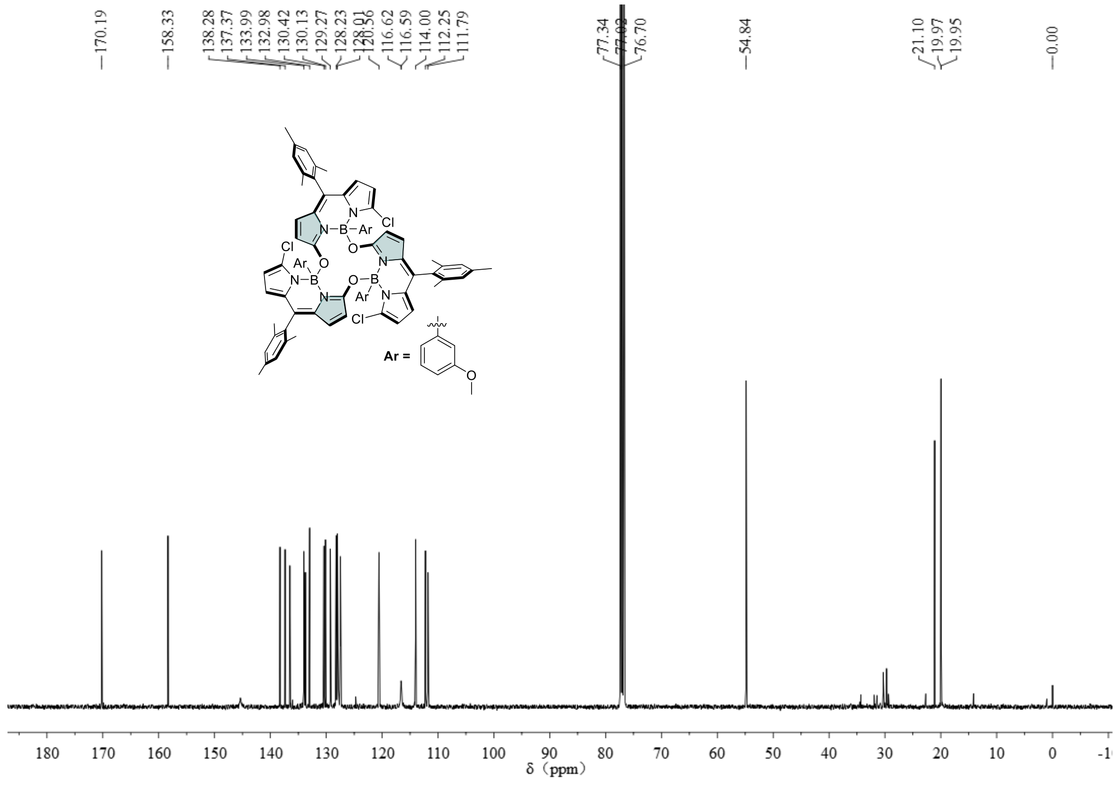

^{13}C NMR spectra of compound **3b**

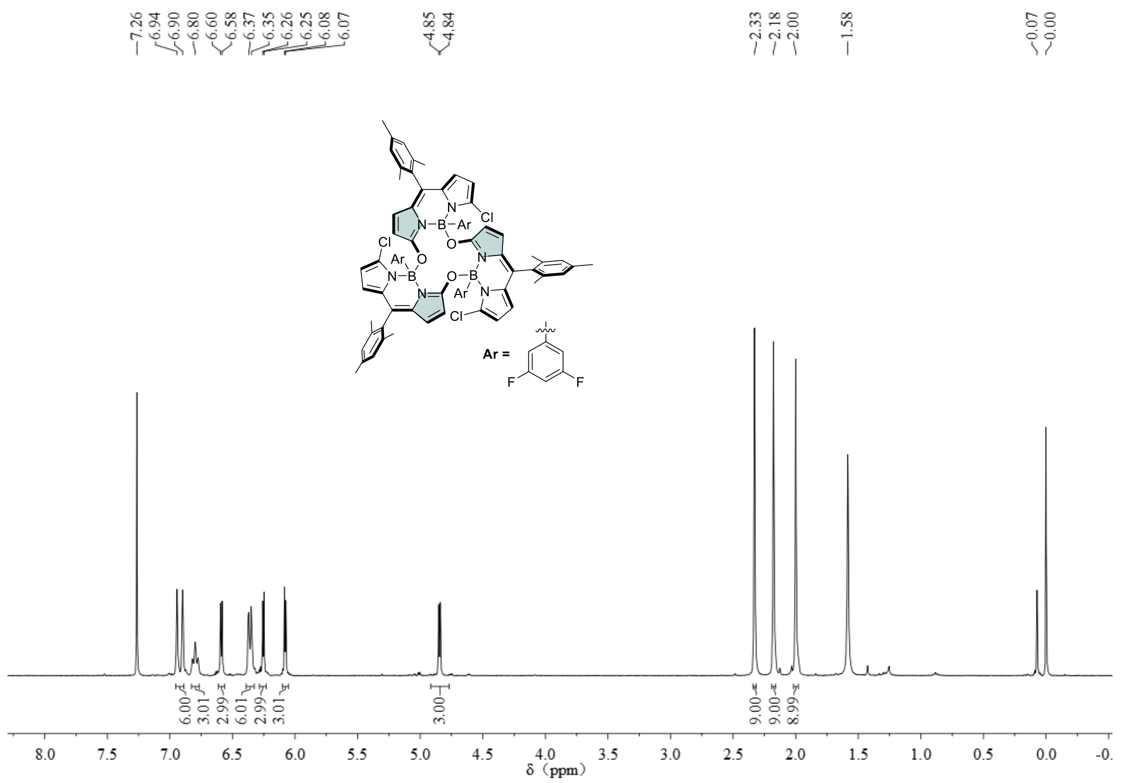

^{1}H NMR spectra of compound **3c**

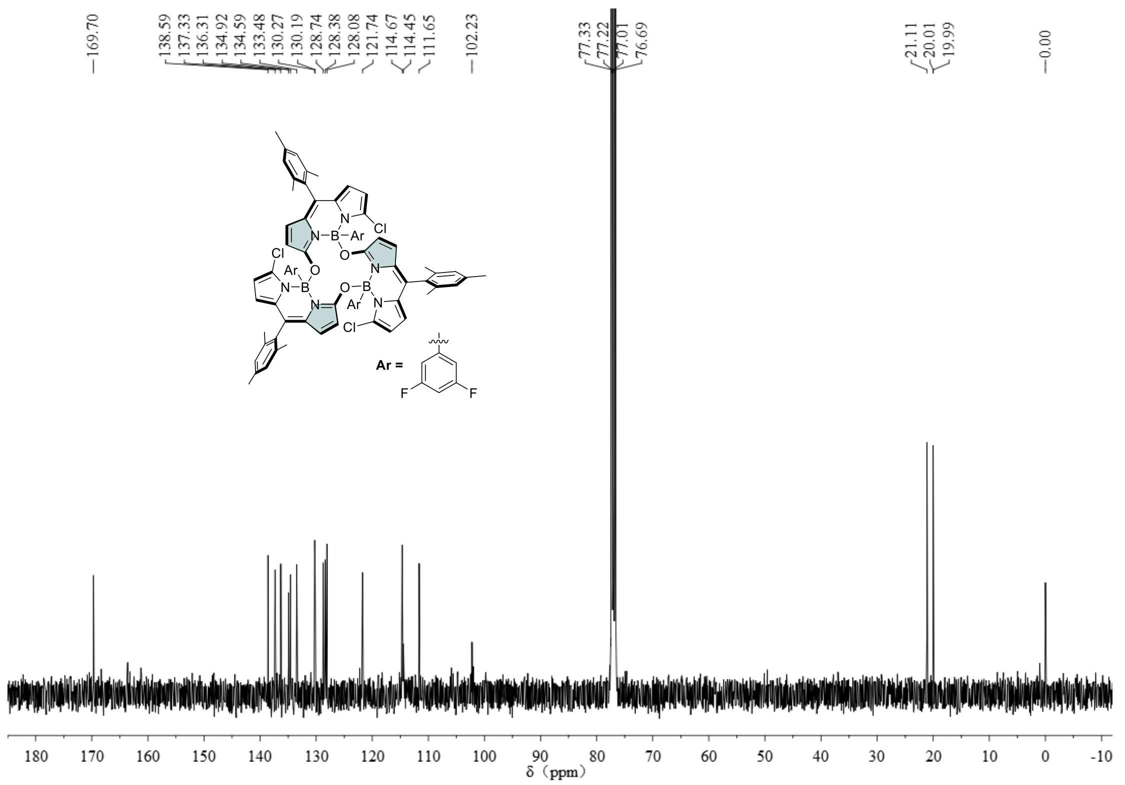

^{13}C NMR spectra of compound **3c**

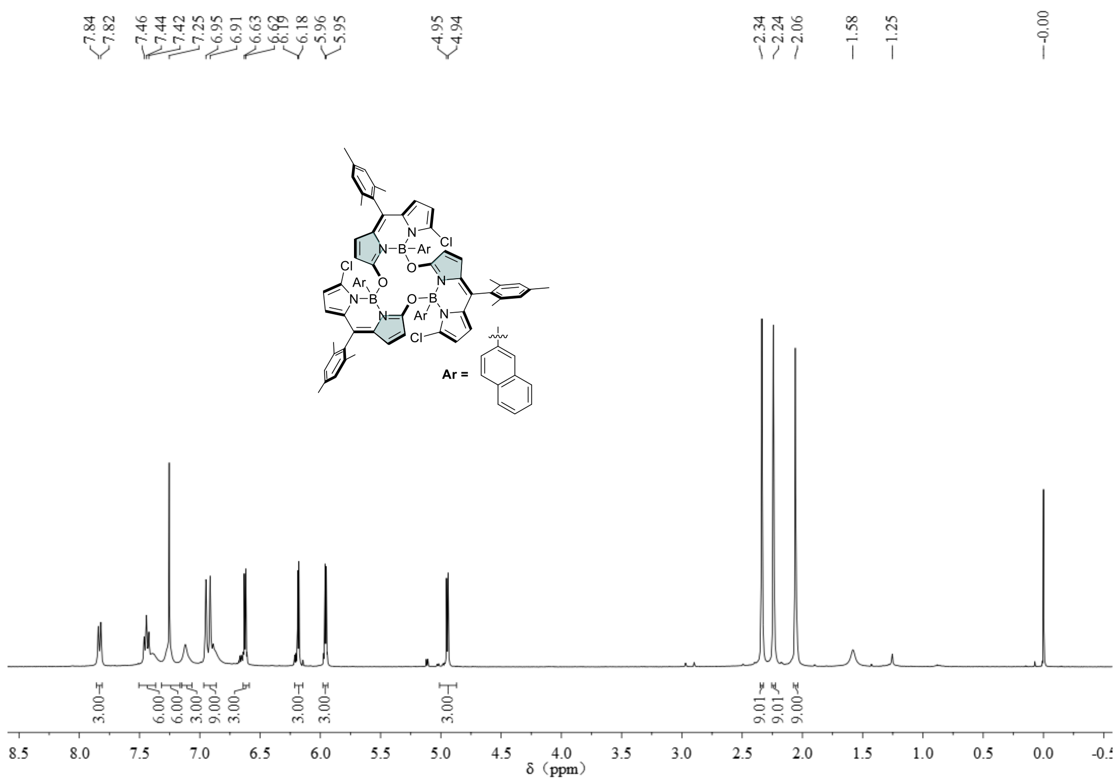

^{1}H NMR spectra of compound **3d**

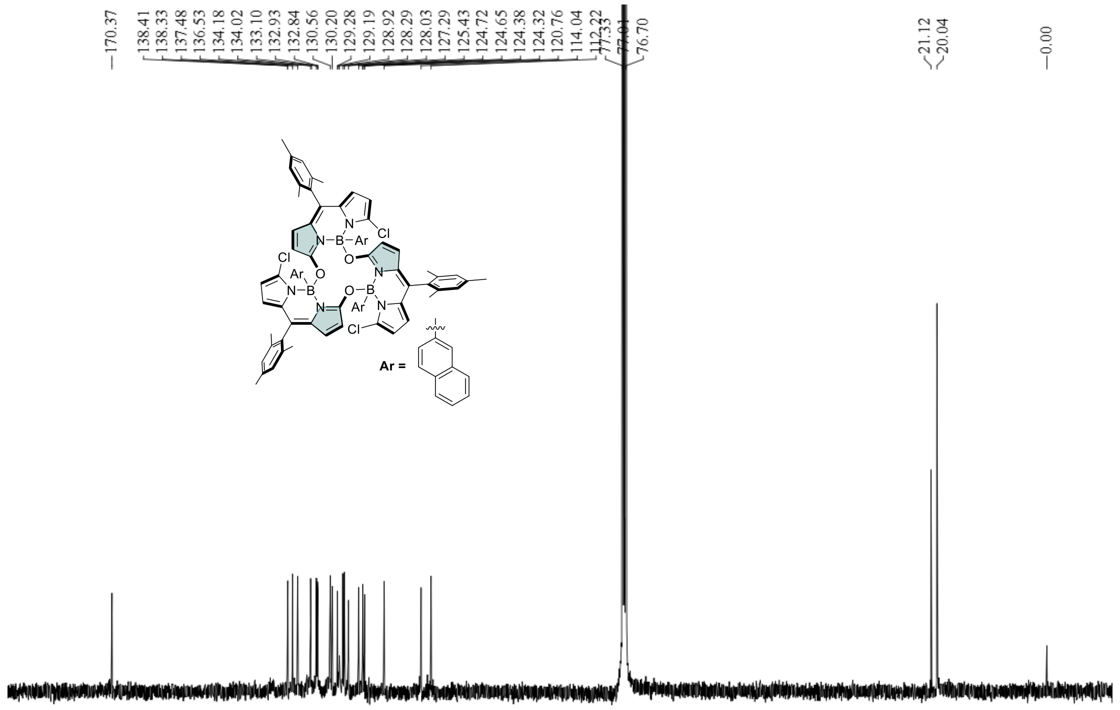

^{13}C NMR spectra of compound **3d**

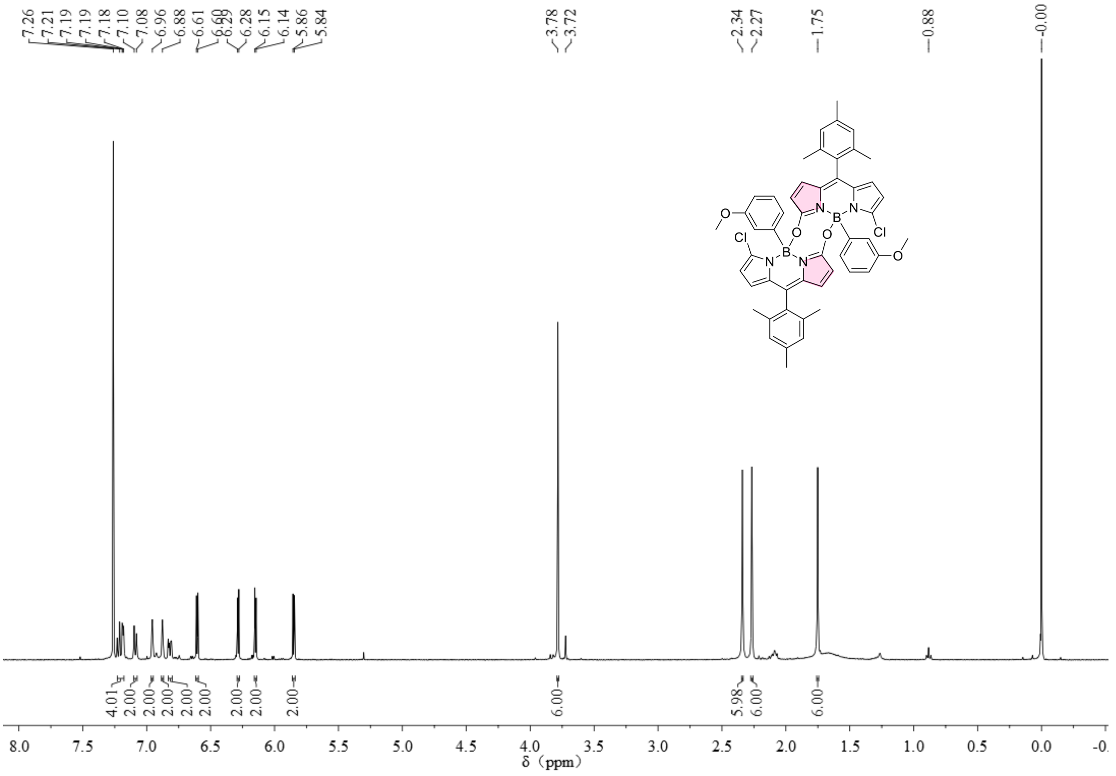

^{1}H NMR spectra of compound **4**

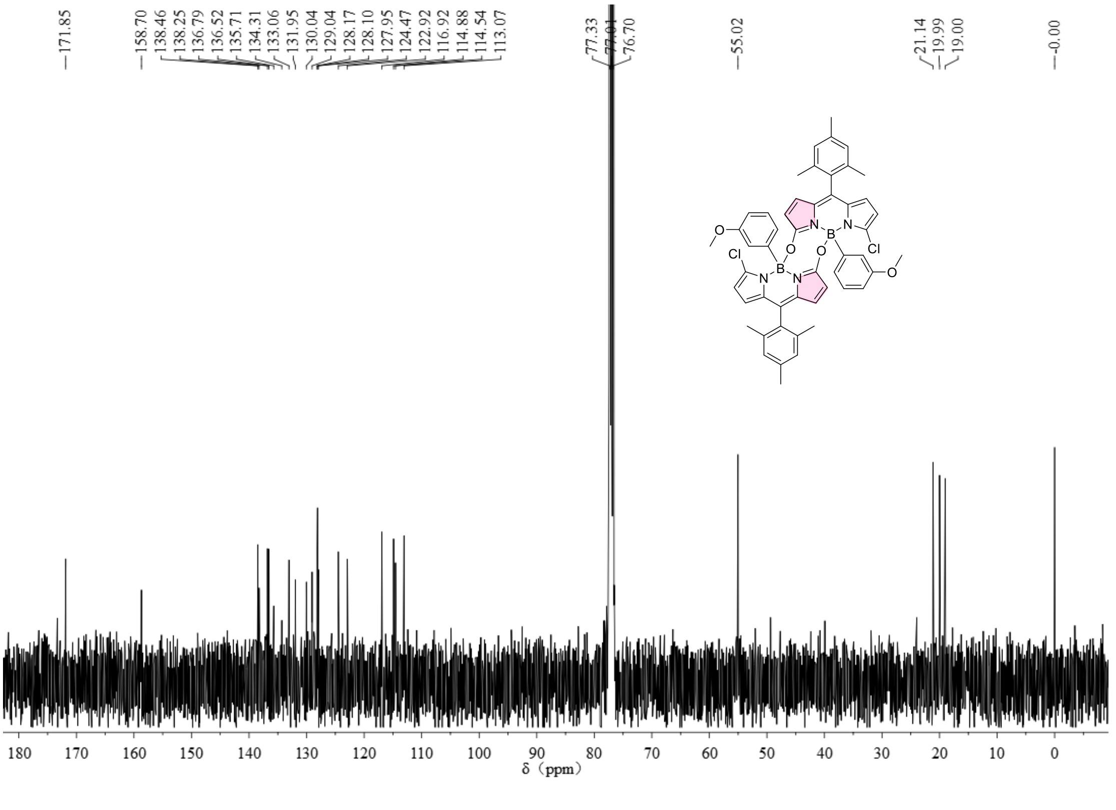

^{13}C NMR spectra of compound **4**

HRMS for 2.

HRMS (APCI) m/z calcd for $C_{18}H_{18}N_2O$ $[M+H]^+$: 313.1102, found 313.1099.

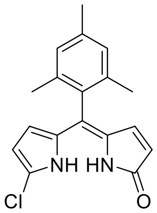

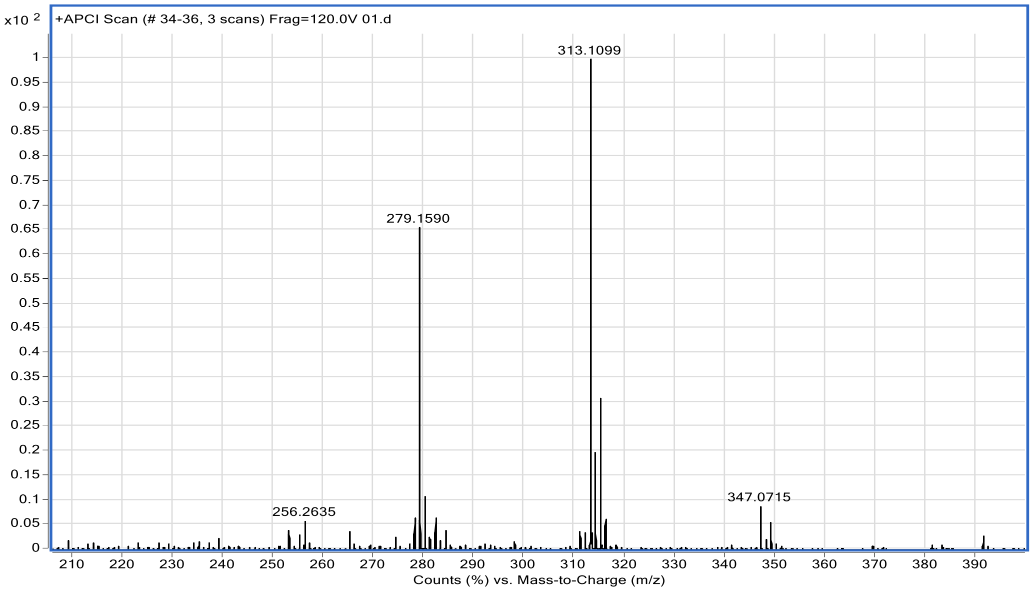

HRMS for 3a.

HRMS (ESI) m/z calcd for $C_{72}H_{60}B_3Cl_3N_6O_3Na^+$ [M+Na]$^+$: 1219.39795, found 1219.39954. ppm = 1.3.

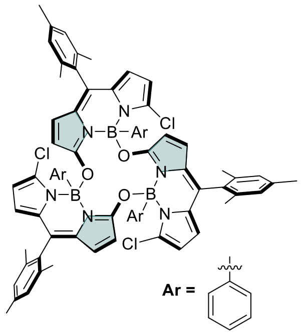

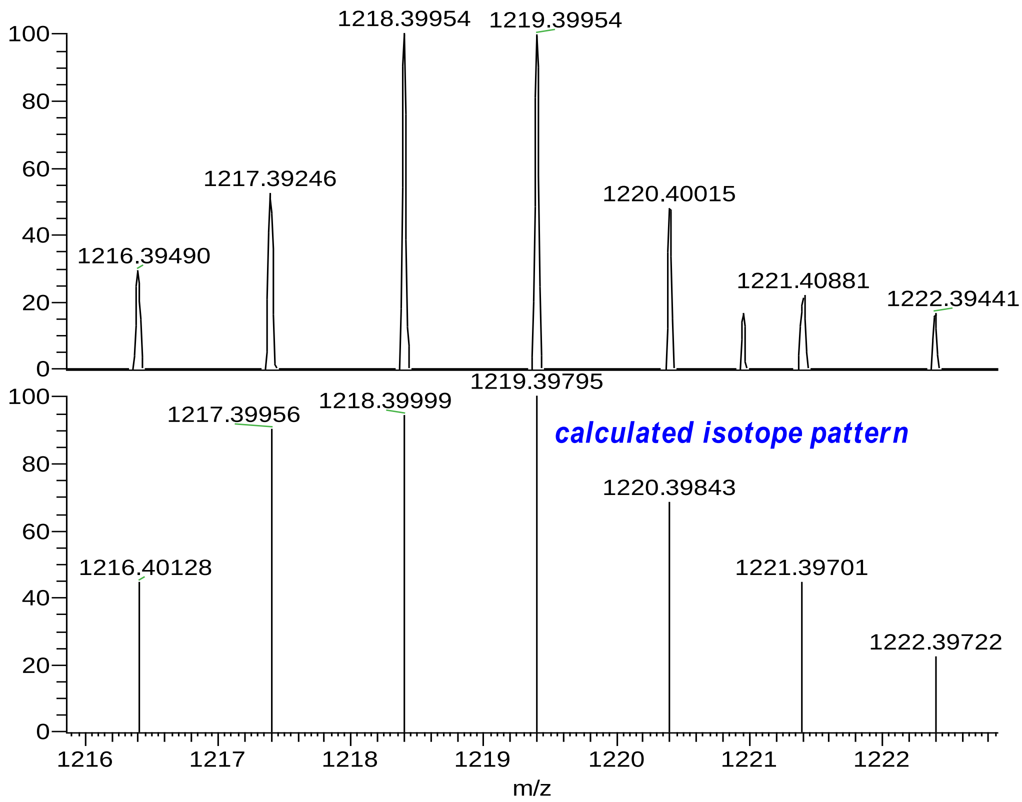

HRMS for 3b. HRMS (ESI) m/z calcd for $C_{75}H_{66}B_3Cl_3N_6O_6Na^+$ $[M+Na]^+$: 1307.42809, found 1307.43030. ppm = 1.7.

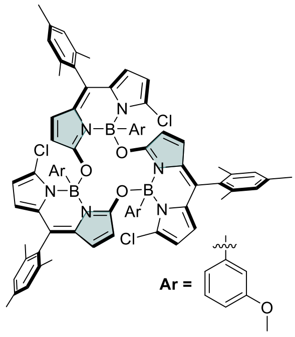

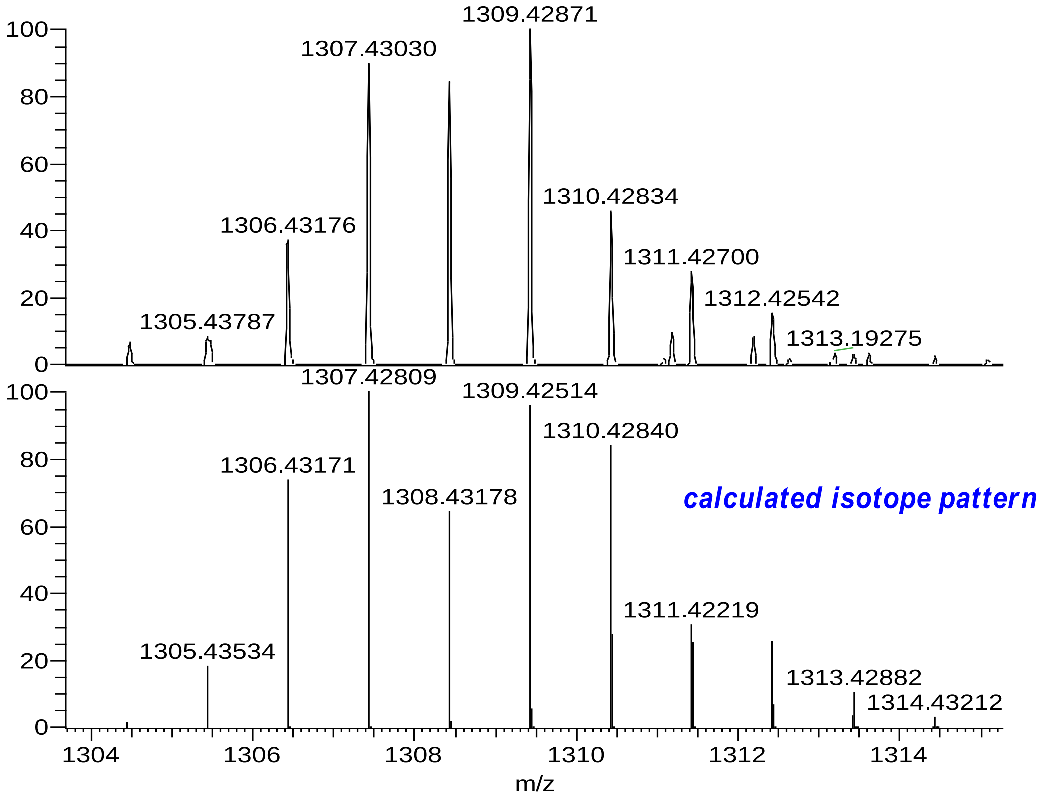

HRMS for 3c.

HRMS (ESI) m/z calcd for $C_{72}H_{55}B_3Cl_3F_6N_6O_3^+$ [M+H]$^+$: 1303.35792, found 1303.35828.

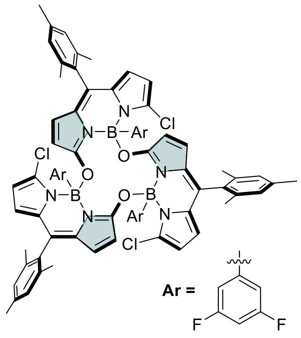

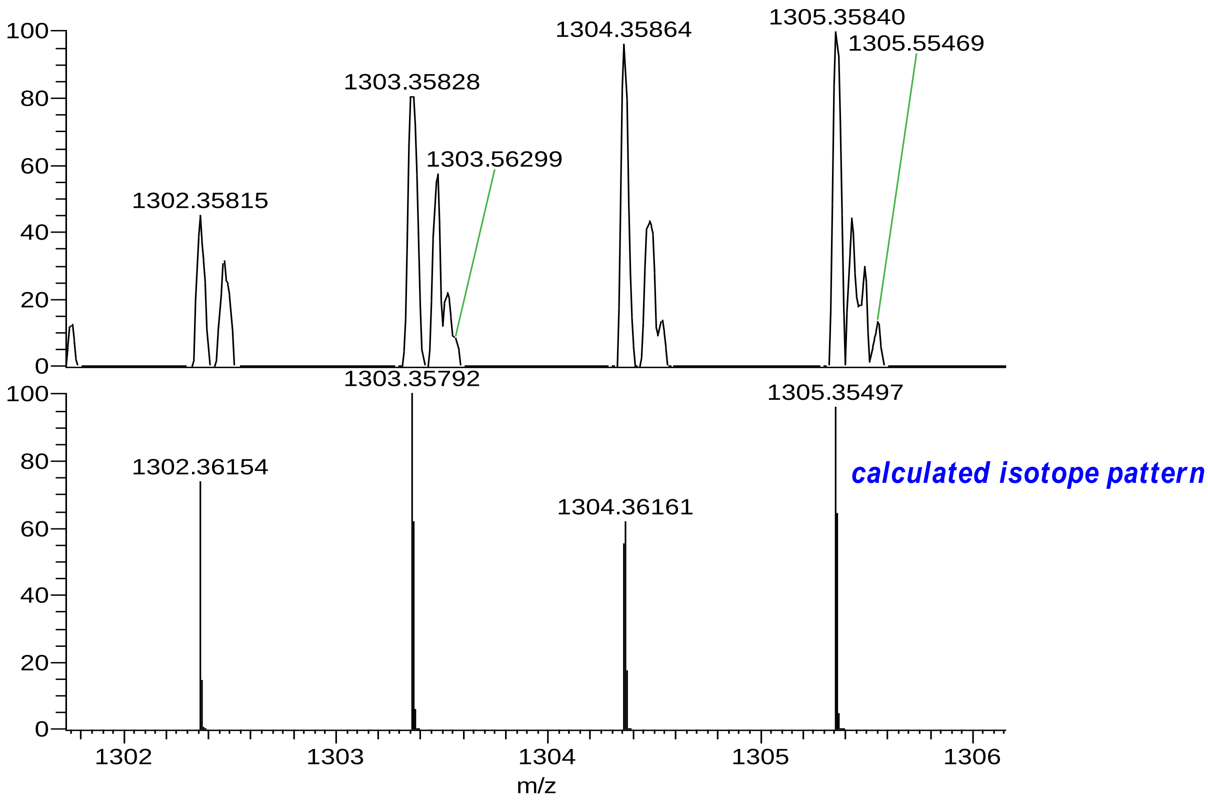

HRMS for 3d.

HRMS (ESI) m/z calcd for $C_{84}H_{66}B_3Cl_3N_6O_3Na^+$ $[M+Na]^+$: 1369.44554, found 1369.44543.

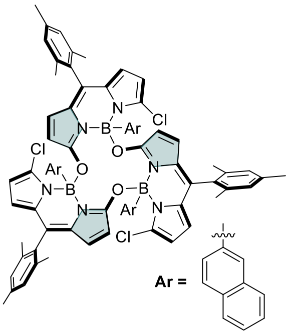

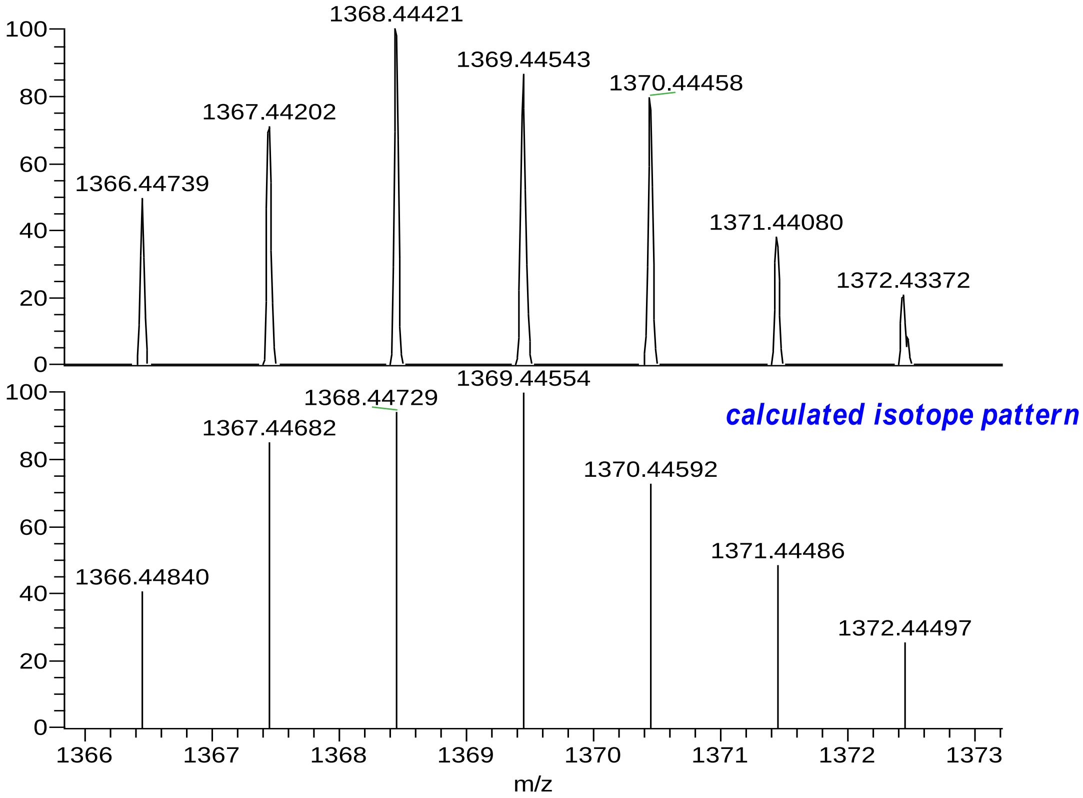

HRMS for 4.

HRMS (ESI) m/z calcd for $C_{50}H_{45}B_2Cl_2N_4O_4^+$ [M+H]$^+$: 857.29985, found 857.30042.

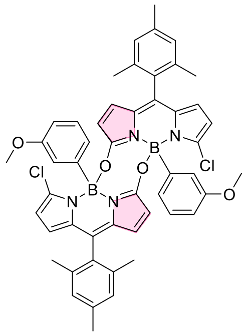

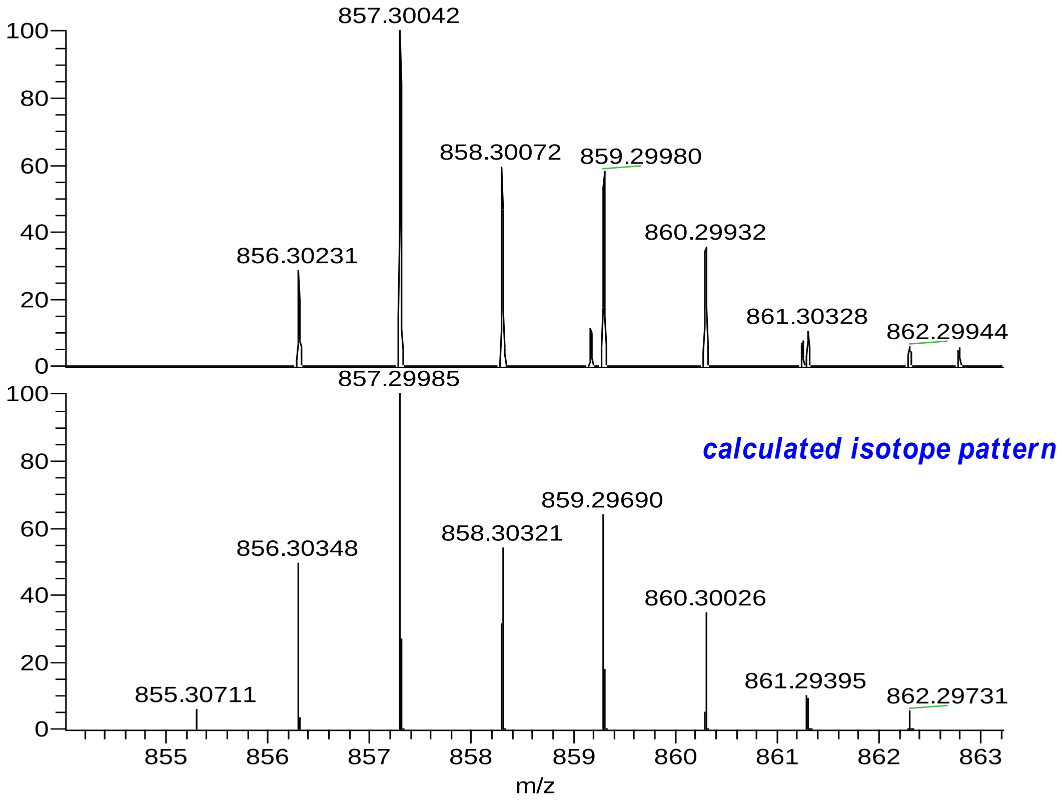

11. Supporting References

1. J. Olmsted, *J. Phys. Chem*., 1979, **83**, 2581.
2. (a) R. C. Benson and H. A. Kues, *Phys. Med. Biol.*, 1978, **23**, 159; (b) J. R. Lakowicz, *Principles of Fluorescence Spectroscopy*, 3rd ed.; Springer: New York, 2006.
3. SAINT V 6.01 (NT) *Software for the CCD Detector System,* Bruker Analytical X-ray Systems, Madison, WI 1999.
4. Sheldrick, G. M. SHELXS-90, *Program for the Solution of Crystal Structure*, University of Göttingen, Germany, 1990.
5. G. M. Sheldrick, *Acta Cryst.,* 2015, **71**, 3.
6. SHELXTL 5.10 (PC/NT-Version), *Program library for Structure Solution and Molecular Graphics*, Bruker Analytical X-ray Systems, Madison, WI **1998**.
7. S. Shimizu, Y. Ito, K. Oniwa, S. Hirokawa, Y. Miura, Q. Matsushita and N. Kobayashi, *Chem. Commum.,* 2012, **48**, 3851.
8. H. Omori, S. Hiroto and H. Shinokubo, *Chem. Commum.,* 2016, **52***,* 3540.
9. Gaussian 09, Revision D.01, M. J. Frisch, G. W. Trucks, H. B. Schlegel, G. E. Scuseria, M. A. Robb, J. R. Cheeseman, G. Scalmani, V. Barone, B. Mennucci, G. A. Petersson, H. Nakatsuji, M. Caricato, X. Li, H. P. Hratchian, A. F. Izmaylov, J. Bloino, G. Zheng, J. L. Sonnenberg, M. Hada, M. Ehara, K. Toyota, R. Fukuda, J. Hasegawa, M. Ishida, T. Nakajima, Y. Honda, O. Kitao, H. Nakai, T. Vreven, J. A. Montgomery, Jr., J. E. Peralta, F. Ogliaro, M. Bearpark, J. J. Heyd, E. Brothers, K. N. Kudin, V. N. Staroverov, T. Keith, R. Kobayashi, J. Normand, K. Raghavachari, A. Rendell, J. C. Burant, S. S. Iyengar, J. Tomasi, M. Cossi, N. Rega, J. M. Millam, M. Klene, J. E. Knox, J. B. Cross, V. Bakken, C. Adamo, J. Jaramillo, R. Gomperts, R. E. Stratmann, O. Yazyev, A. J. Austin, R. Cammi, C. Pomelli, J. W. Ochterski, R. L. Martin, K. Morokuma, V. G. Zakrzewski, G. A. Voth, P. Salvador, J. J. Dannenberg, S. Dapprich, A. D. Daniels, O. Farkas, J. B. Foresman, J. V. Ortiz, J. Cioslowski, and D. J. Fox, Gaussian, Inc., Wallingford CT, 2013; (b) Z. Rinkevicius, I. Tunell, P. Sałek, O. Vahtras, H. Ågren, *J. Chem. Phys.* **2003**, *119*, 34−46; (c) Helgaker, T., Jørgensen, P., Olsen, J., Ruud, K., Andersen, T., Bak, KL., Bakken, V., Christiansen, O., Dahle, P., Dalskov, EK., Enevoldsen, T., Heiberg, H., Hettema, H., Jonsson, D., Kirpekar, S., Kobayashi, R., Koch, H., Mikkelsen, KV., Norman, P., Packer, MJ., Saue, T., Taylor, PR., Vahtras, O., Jensen, HJA., Ågren, H. *Dalton, an Ab Initio Electronic Structure Program*, 1.0. **1997**; (d) S. Koseki, M. Schmidt, M. Gordon, *J. Phys. Chem.* **1990**, *96*, 10678.
